# The collateral activity of RfxCas13d can induce lethality in a RfxCas13d knock-in mouse model

**DOI:** 10.1186/s13059-023-02860-w

**Published:** 2023-02-01

**Authors:** Yunfei Li, Junjie Xu, Xuefei Guo, Zhiwei Li, Lili Cao, Shengde Liu, Ying Guo, Guodong Wang, Yujie Luo, Zeming Zhang, Xuemei Wei, Yingchi Zhao, Tongtong Liu, Xiao Wang, Huawei Xia, Ming Kuang, Qirui Guo, Junhong Li, Luoying Chen, Yibing Wang, Qi Li, Fengchao Wang, Qinghua Liu, Fuping You

**Affiliations:** 1 Department of Immunology, School of Basic Medical Sciences, Beijing Key Laboratory of Tumor Systems Biology, Institute of Systems Biomedicine, Peking University Health Science Center, Beijing, 100191 China; 2 National Institute of Biological Sciences, Tsinghua Institute of Multidisciplinary Biomedical Research, Tsinghua University, Beijing, 102206 China; 3 College of Life Sciences, Beijing Normal University, Beijing, 100875 China; 4 School of Life Sciences, Institute of Life Sciences and Green Development, Hebei University, Baoding, 071002 Hebei China; 5 Beijing Institutes of Life Science, Chinese Academy of Sciences, Beijing, 100101 China; 6 Department of Gastrointestinal Oncology, Key Laboratory of Carcinogenesis and Translational Research (Ministry of Education/Beijing), Peking University Cancer Hospital and Institute, Beijing, 100142 China

**Keywords:** RfxCas13d, Collateral activity, Death of mice, 28s rRNA cleavage

## Abstract

**Background:**

The CRISPR-Cas13 system is an RNA-guided RNA-targeting system and has been widely used in transcriptome engineering with potentially important clinical applications. However, it is still controversial whether Cas13 exhibits collateral activity in mammalian cells.

**Results:**

Here, we find that knocking down gene expression using RfxCas13d in the adult brain neurons caused death of mice, which may result from the collateral activity of RfxCas13d rather than the loss of target gene function or off-target effects. Mechanistically, we show that RfxCas13d exhibits collateral activity in mammalian cells, which is positively correlated with the abundance of target RNA. The collateral activity of RfxCas13d could cleave 28s rRNA into two fragments, leading to translation attenuation and activation of the ZAKα-JNK/p38-immediate early gene pathway.

**Conclusions:**

These findings provide new mechanistic insights into the collateral activity of RfxCas13d in mammalian cells and warn that the biosafety of the CRISPR-Cas13 system needs further evaluation before application to clinical treatments.

**Supplementary Information:**

The online version contains supplementary material available at 10.1186/s13059-023-02860-w.

## Background

Clustered regularly interspaced short palindromic repeats (CRISPR) and accompanying CRISPR-associated (Cas) proteins constitute the adaptive CRISPR-Cas immune system in bacteria and archaea, which protects the bacteria from invaders, including phages and mobile genetic elements. The defense process can be divided into three stages: Adaptation, incorporation of foreign DNA fragments into CRISPR array as spacers; CRISPR RNA (crRNA) biogenesis, CRISPR array is transcribed into a long precursor crRNA (pre-crRNA), and then processed into mature crRNAs; Interference, Cas effector proteins, under the guidance of crRNAs, specifically recognize and cleave foreign genetic elements. The rapid evolutionary arms race between bacteria and mobile genetic elements has greatly enriched CRISPR-Cas systems, which have been harnessed for various research and therapeutic applications. According to the structure and function of Cas effector proteins, CRISPR-Cas systems can be categorized into two classes, which are further subdivided into six types (types I–VI). Class 1 effectors comprise of multiple subunits, including type I, III, and IV, while class 2 effectors are single large proteins, including type II, V, and VI [1]. Due to their simplicity, Class 2 CRISPR-Cas systems have been widely developed as genome editing and transcriptional regulating tools, such as DNA-targeting Cas9 and Cas12, RNA-targeting Cas13.

Cas13 was originally found by mining microbial genome sequencing data using the highly conserved adaption protein Cas1 as the anchor [2]. Protein sequence alignments revealed that Cas13 contains two higher eukaryotes and prokaryotes nucleotide-binding (HEPN) domains and is predicted to possess ribonuclease (RNase) activity [2]. It was confirmed by subsequent experiments that the Cas13-crRNA complex recognizes and cleaves the target RNA via base pairing between the crRNA and the target RNA [3]. Once the Cas13-crRNA complex binds to the target RNA, it forms an active ternary ribonucleoprotein (RNP) complex. This ternary RNP complex undergoes conformational changes which trigger the two HEPN domains to come close proximity to each other and form a composite RNase active site that is exposed on the surface of the complex [4, 5]. This RNase activity not only specifically cleaves the target RNA, but also promiscuously cleaves bystander RNAs (known as collateral activity) [4–6]. Cas13 exhibits obvious collateral activity in bacteria and in vitro, which thus has been ingeniously developed as a molecular diagnostic tool [7–9]. However, this collateral activity has not been detected in mammals. Theoretically, compared with Cas9-mediated gene knockout technology, Cas13 can accurately distinguish different transcripts of the same gene, and then study their function individually. And Cas13-mediated gene silencing does not change genomic DNA, so this gene silencing is reversible and considered safer than Cas9, which has advantages over Cas9 in the treatment of some acquired diseases. Moreover, accumulating evidence over the past decade highlights that noncoding RNAs play essential roles in various cellular processes [10]. Cas13 is more suitable for noncoding RNA research than Cas9.

Currently, there are six subtypes identified in the Cas13 family, including Cas13a, Cas13b, Cas13c, Cas13d, Cas13X, and Cas13Y [2, 7, 11–14]. Since their discovery, Cas13 subtypes, such as LwaCas13a, PspCas13b, RfxCas13d, and Cas13X.1, have been widely used in knockdown experiments in mammalian cells, exhibiting higher efficiency and specificity than traditional RNA interference, and no detected collateral activity [3, 7, 12, 14]. Among these Cas13 subtypes, due to its advantages in efficiency and size, RfxCas13d was applied in mammals via adeno-associated virus (AAV) delivery, with no side effects reported [15–17]. But several research groups have different opinions about the collateral activity of Cas13. Kang group first reported that the collateral activity of LwaCas13a occurred in U87 cells, non-specifically cleaving non-target RNAs, leading to cell death [18]. Later, they and collaborators reported that this phenomenon also existed in HepG2, AT2, B16F10, and GL261 cells [19, 20]. Following their work, Gootenberg and Abudayyeh group reported that collateral activity was detected in U87 cells for LwaCas13a, PspCas13b, and RfxCas13d, and in HepG2 and mES cells for RfxCas13d [21]. Yang group claimed that LwaCas13a, RfxCas13d, and Cas13X.1 exhibited collateral activity when targeting transiently overexpressing mCherry, but not endogenous genes in HEK293T cells [14]. However, these studies did not figure out what effects the collateral activity of Cas13 has on mammalian cells. Therefore, it is still controversial whether the collateral activity of Cas13 exists in mammalian cells. More importantly, the safety of applying Cas13 to treatment needs to be carefully evaluated in animal models.

More recently, several studies reported that Cas13 exhibited collateral effects in mammalian cells, including one that further reported the lethality of Cas13 in mice [22–25]. In line with those recent studies, we found that mice died when using RfxCas13d to knock down genes in brain neurons. RfxCas13d-mediated lethality may result from the collateral activity of RfxCas13d instead of the loss of target gene function or off-target effects. And we confirmed that RfxCas13d exhibited collateral activity in mammalian cells, which is positively correlated with the abundance of target RNA. Furthermore, we found that the collateral activity of RfxCas13d cleaved 28s rRNA into two fragments, leading to translation attenuation and activation of the ZAKα-JNK/p38-immediate early gene (IEG) pathway.

## Results

### Mice died when knocking down Sik3-S in neurons using RfxCas13d

**Fig. 1:**
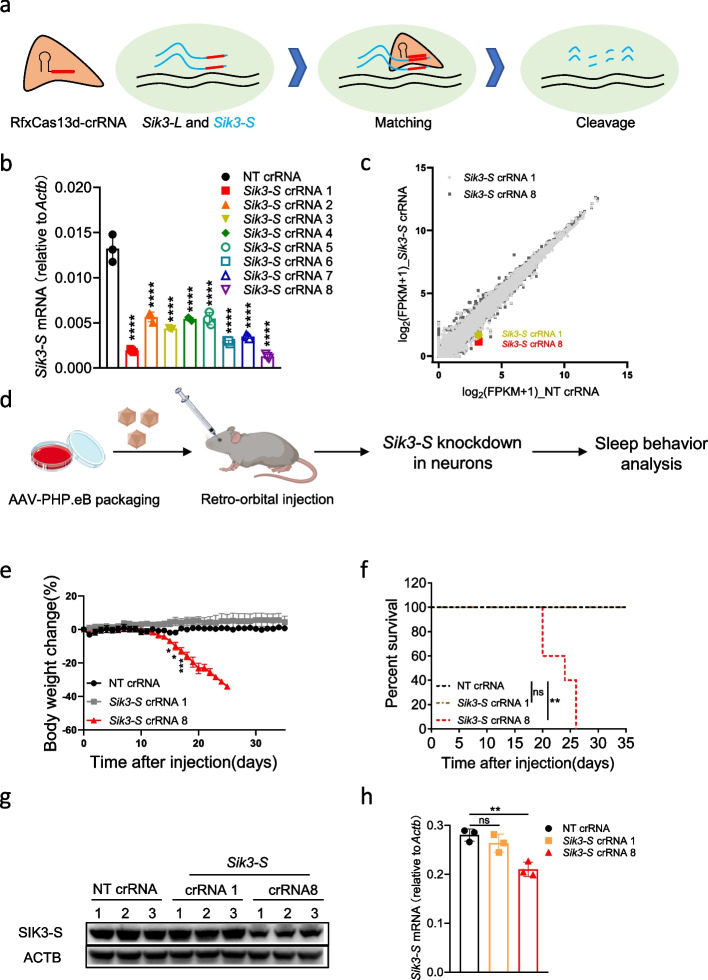
Mice died when knocking down *Sik3-S* in neurons using RfxCas13d. **a** Schematic illustration of RfxCas13d-mediated specifically knockdown of *Sik3-S* not *Sik3-L* mRNA. *Sik3-L*: a transcript encoding a long isoform of SIK3. **b** RT-qPCR to measure the knockdown efficiency of *Sik3-S* crRNAs (*n*=3). **c** Transcriptome analysis in N2a cells 48 h post-transfection of plasmids encoding RfxCas13d and *Sik3-S* crRNAs (*n*=3) or NT crRNA (*n*=3). **d** Workflow of knocking down *Sik3-S* in the adult mouse brain neurons. **e** Body weight change curve of ^LSL^RfxCas13d^fl/fl^ mice after injection of AAV-PHP.eB carrying *Sik3-S* crRNAs (*n*=5) or NT crRNA (*n*=5). **f** Survival curve of ^LSL^RfxCas13d^fl/fl^ mice after injection of AAV-PHP.eB carrying *Sik3-S* crRNAs (*n*=5) or NT crRNA (*n*=5). **g**, **h** Western blot and RT-qPCR to measure SIK-S/*Sik3-S* expression level in brain lysates of ^LSL^RfxCas13d^fl/fl^ mice injected with AAV-PHP.eB carrying *Sik3-S* crRNAs (*n*=3) or NT crRNA (*n*=3) at 20 dpi. **b**, **h** One-way ANOVA with Dunn’s multiple comparisons test. **e** Two-tail unpaired *t* test. **f** Log-rank test. Significance levels are noted as *P < 0.05, **P < 0.01, ***P < 0.001, ****P < 0.0001 or ns (*P* > 0.05). All values are presented as mean ± SEM

A recent study identified a *Sleepy (Sik3*^*Slp/+*^*)* mouse strain, which carries a mutation in the gene encoding salt-inducible kinase 3 (SIK3), a member of the AMP-activated protein kinase (AMPK) family [26]. The *Sleepy (Sik3*^*Slp/+*^*)* mice exhibit over 4 h increase in daily non-rapid eye movement sleep (NREMS) time and constitutively elevated NREMS delta power relative to wild-type (WT) littermates [26]. Interestingly, the *Sik3* gene encodes multiple transcripts due to alternative splicing. And our recent study identified a new *Sik3-S* transcript encoding ~72 kDa short isoform of SIK3 (SIK3-S) [27, 28] (Additional file 1: Fig. S1a). To investigate the role of SIK3-S in sleep regulation, RfxCas13d was leveraged to specifically knock down *Sik3-S* by taking advantage of its ability to distinguish different transcripts of the same gene (Fig. 1a). We designed eight crRNAs targeting *Sik3-S* and examined their knockdown efficiency in mouse neuroblastoma N2a cells through RT-qPCR (Additional file 1: Fig. S1b). Our results showed that, in collaboration with RfxCas13d, all eight crRNAs, especially crRNA 1 and 8, caused significant knockdown of *Sik3-S* expression (Fig. 1b). Transcriptome analysis revealed that *Sik3* was downregulated while most of the other genes remained unchanged when RfxCas13d was co-transfected with *Sik3-S* crRNA 1 or 8, compared with non-targeting (NT) crRNA (Fig. 1c and Additional file 2: Table S1).

To knock down *Sik3-S* expression in the adult brain neurons, we generated the ^LSL^RfxCas13d^fl/fl^ knock-in mice by inserting the CAG-loxP-STOP-loxP-RfxCas13d cassette into the *Rosa26* locus by homologous recombination using CRISPR/Cas9 technology and ensured that they have the expected genotype by genotyping (Additional file 1: Fig. S2a-b). We counted the numbers of different genotypes in six litters of mice (^LSL^RfxCas13d^+/fl^ x ^LSL^RfxCas13d^+/fl^) and found that they obeyed Mendel’s laws of inheritance, indicating that insertion of CAG-loxP-STOP-loxP-RfxCas13d into the *Rosa26* locus did not affect mouse viability (Additional file 1: Fig. S2c-d). Moreover, we confirmed that the expression of RfxCas13d can be released by hSYN (human synapsin 1 gene promoter)-driven Cre and no other possible RfxCas13d variant existed (Additional file 1: Fig. S3 and S4a). As shown in Fig. 1d, we retro-orbitally injected 12-week-old ^LSL^RfxCas13d^fl/fl^ adult mice with AAV-PHP.eB to deliver a systemic expression of U6-driven crRNA and hSYN-driven Cre (Additional file 1: Fig. S4b). AAV-PHP.eB could efficiently cross the blood-brain barrier and transduce the majority of neurons and astrocytes across the adult mouse brain [27, 29]. hSYN restricted the expression of Cre recombinase in neurons. Subsequently, Cre recombinase mediated excision of a tripartite transcriptional stop cassette (STOP) flanked by loxP to release the expression of RfxCas13d. Finally, RfxCas13d, under the guidance of *Sik3-S* crRNAs, specifically recognized and cleaved *Sik3-S* transcripts. Unexpectedly, mice injected with AAV-PHP.eB containing *Sik3-S* crRNA 8 began to lose weight at ~20 days post injection (dpi) and died at ~24 dpi (Fig. 1e,f). And SIK3-S was significantly knocked down in *Sik3-S* crRNA 8 group, compared with NT crRNA group (Fig. 1g,h). This phenomenon prevented our experiments from continuing but aroused our curiosity-why mice died when knocking down *Sik3-S* using RfxCas13d in the adult mouse brain neurons.

### RfxCas13d-mediated lethality was not due to the loss of target gene function

**Fig. 2:**
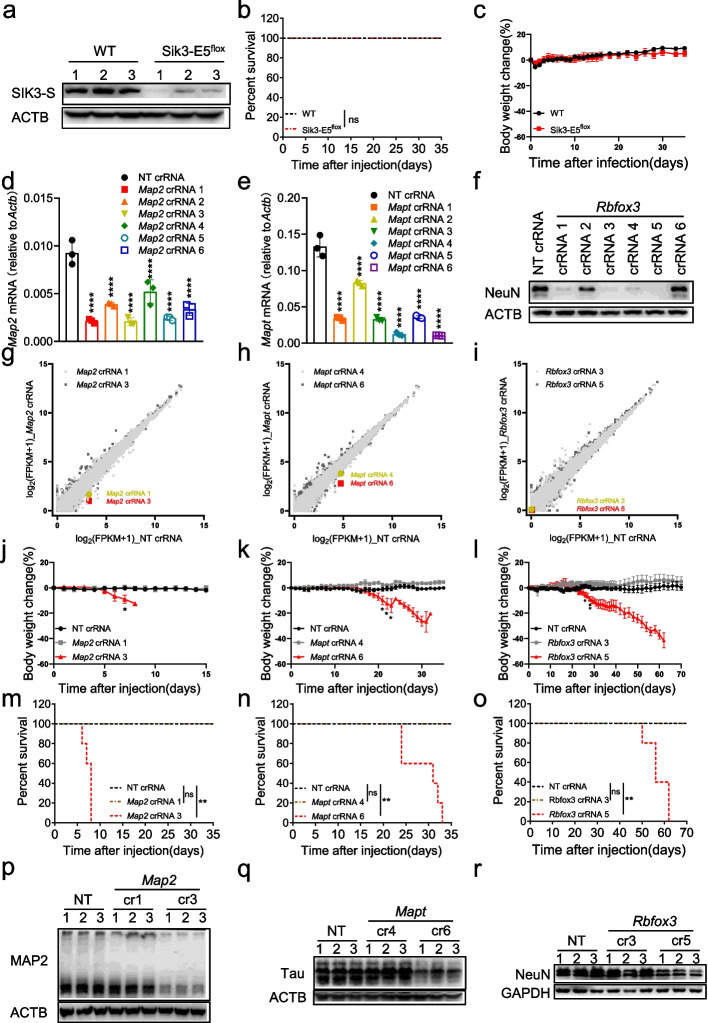
RfxCas13d-mediated lethality was not due to the loss of target gene function. **a** Western blot to measure SIK3-S expression level in brain lysates from Sik3-E5^fl/fl^ and WT mice at 21 dpi. **b** Survival curve of Sik3-E5^fl/fl^ (*n*=5) and WT (*n*=5) mice after injection of AAV-PHP.eB-hSYN-Cre. **c** Body weight change curve of Sik3-E5^fl/fl^ (*n*=5) and WT (*n*=5) mice after injection of AAV-PHP.eB-hSYN-Cre. **d**, **e** RT-qPCR to measure the knockdown efficiency of *Map2* (*n*=3) and *Mapt* (*n*=3) crRNAs in N2a cells. **f** Western blot to measure the knockdown efficiency of *Rbfox3* crRNAs by knocking down overexpressed NeuN in HEK293T cells using RfxCas13d. **g**–**i** Transcriptome analysis in N2a cells 48 h after transfection of plasmids encoding RfxCas13d and crRNAs (*n*=3). **j**–**l** Body weight change curve of ^LSL^RfxCas13d^fl/fl^ mice after infection of PHP.eB carrying NT crRNA (*n*=5), *Map2* crRNAs (*n*=5), *Mapt* crRNAs (*n*=5), or *Rbfox3* crRNAs (*n*=5). **m**–**o** Survival curve of ^LSL^RfxCas13d^fl/fl^ mice after injection of AAV-PHP.eB carrying NT crRNA (*n*=5), *Map2* crRNAs (*n*=5), *Mapt* crRNAs (*n*=5), or *Rbfox3* crRNAs (*n*=5). **p**–**r** Western blot to measure MAP2 (at 6 dpi), Tau (at 25 dpi), or NeuN (at 50 dpi) expression level in brain lysates of ^LSL^RfxCas13d^fl/fl^ mice after injection of AAV-PHP.eB carrying NT crRNA, *Map2* crRNAs, *Mapt* crRNAs or *Rbfox3* crRNAs. **d**, **e** One-way ANOVA with Dunn’s multiple comparisons test. **j**–**l** Two-tail unpaired *t* test. **b**, **m**-**o** Log-rank test. Significance levels are noted as *P < 0.05, **P < 0.01, ****P < 0.0001 or ns (*P* > 0.05). All values are presented as mean ± SEM

Several previous studies have proved that RfxCas13d can be used to knock down endogenous genes in vivo with no reported side effects in the liver, brain, and eyes [15–17]. In addition, although the homozygous *Sik3* knockout mice can be created but exhibit impaired chondrocytes during development, neonatal lethality, and reduced size, indicating that *Sik3* is essential for mouse health and survival [30, 31]. Therefore, we first guessed whether the death of mice was caused by the downregulation of *Sik3-S*. To test this, we intended to knock out *Sik3* in the same neurons of mice by the conventional Cre-loxP system. We generated Sik3-E5^fl/fl^ mice by inserting two loxP sites into both sides of exon 5 in the *Sik3* gene locus and then delivered AAV-PHP.eB carrying hSYN-driven Cre into Sik3-E5^fl/fl^ mice to knock out *Sik3*, with WT mice as control (Additional file 1: Fig. S4a and S5). At 21 dpi, brain lysates from Sik3-E5^fl/fl^ mice showed lower SIK3-S expression levels compared with WT mice, indicating that SIK3-S was knocked out in brain neurons (Fig. 2a). But Sik3-E5^fl/fl^ mice behaved as normal as WT mice without loss of body weight or death (Fig. 2b,c), which means that knocking out *Sik3* in neurons will not cause mouse death. Thus, the mouse lethality that occurred when using RfxCas13d to knock down *Sik3-S* expression had nothing to do with the loss of functional SIK3-S.

Map2, Tau, and NeuN were well-characterized neuron marker genes. Homozygous knockout of each of these genes did not lead to death of mice [32–34]. We thus selected them as targets and knocked down each of three targets in the same way as knocking down *Sik3-S* in vivo. In theory, when knocking down these genes individually using RfxCas13d in adult mouse brain neurons, the mice will not die due to the loss of these genes. We designed six crRNAs for each gene and tested their knockdown efficiency in N2a cells by RT-qPCR (Fig. 2d,e). Since the expression level of NeuN is too low in N2a cells, we tested the efficiency of crRNAs by knocking down the expression of co-transfected NeuN plasmid in HEK293T (Fig. 2f). The two best crRNAs for each gene were chosen for follow-up experiments. Transcriptome analysis showed that these three genes were knocked down using corresponding crRNAs in tandem with RfxCas13d, while most of the other genes remained unchanged (Fig. 2g,i and Additional file 2: Table S1). Following the same protocol of knocking down *Sik3-S* in vivo, we knocked down these three genes respectively in ^LSL^RfxCas13d^fl/fl^ mice. Results showed that mice in *Map2* crRNA 3, *Mapt* crRNA 6, and *Rbfox3* crRNA 5 groups showed significant loss of body weight and death, while mice in the other groups behaved normally and survived (Fig. 2j–o). Besides, brain lysates showed that these three target genes were downregulated in corresponding death groups (Fig. 2p–r).

Taken together, these data suggested that RfxCas13d-mediated mouse death was not due to the loss of target gene function.

### RfxCas13d-mediated lethality may result from the collateral activity of RfxCas13d rather than off-target effects

Since the death of mice was not related to the loss of function of the target genes, was it due to the defects of RfxCas13d itself? Off-target effects are an ongoing concern for gene editing using any CRISPR-Cas system [35]. Besides, collateral activity should be considered, although multiple studies have used RfxCas13d in vivo with no reported side effects [15–17]. Off-target effects can be defined as unintended cleavage at untargeted DNA/RNA sites showing a similar but not an identical sequence compared to the target site [36, 37]. Whereas the collateral activity of Cas13 is triggered by the binding of the target RNA to promiscuously cleave nearby RNAs [4, 5]. Both cleave untargeted RNAs but differ in the mechanism. Thus, it is necessary to determine whether RfxCas13d-mediated mouse death was caused by the collateral activity or off-target effects of RfxCas13d.

**Fig. 3:**
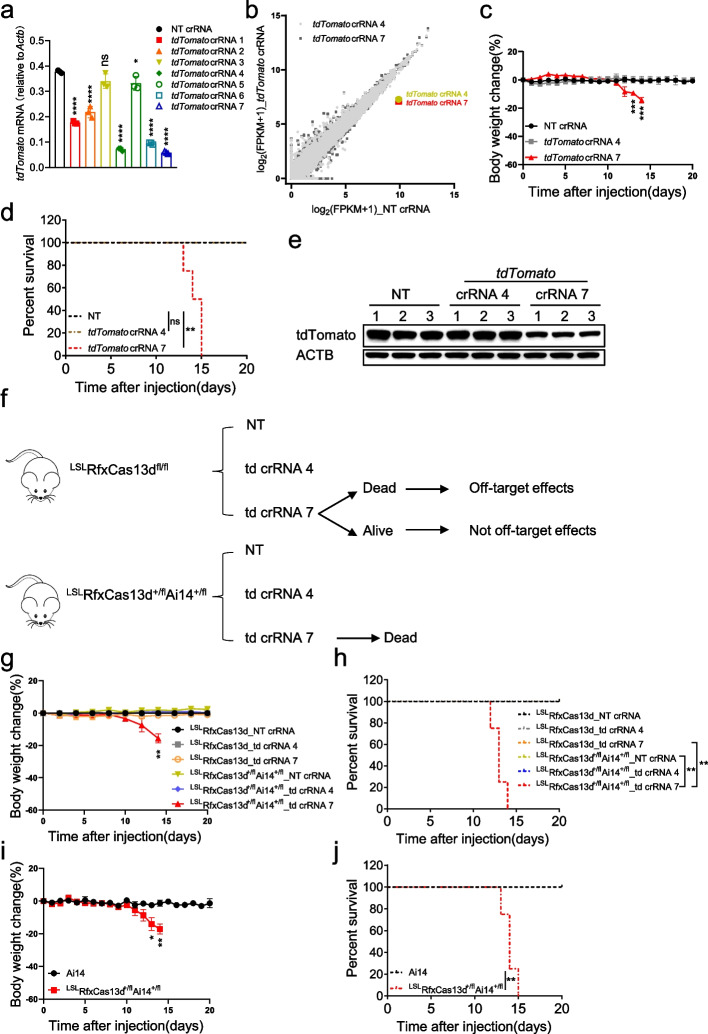
RfxCas13d-mediated lethality may result from the collateral activity of RfxCas13d rather than off-target effects. **a** RT-qPCR to measure the knockdown efficiency of tdTomato crRNAs (*n*=3) in N2a cells stably expressing tdTomato (N2a-td). **b** Transcriptome analysis of N2a-td cells 48 h after transfection of plasmids encoding RfxCas13d and tdTomato crRNAs (*n*=3) or NT crRNA (*n*=3). **c** Body weight change curve of ^LSL^RfxCas13d^+/fl^Ai14^+/fl^ mice after injection of AAV-PHP.eB carrying tdTomato crRNAs (*n*=4) or NT crRNA (*n*=4). **d** Survival curve of ^LSL^RfxCas13d^+/fl^Ai14^+/fl^ mice after injection of AAV-PHP.eB carrying tdTomato crRNAs (*n*=4) or NT crRNA (*n*=4). **e** Western blot to measure tdTomato expression level in brain lysates of ^LSL^RfxCas13d^+/fl^Ai14^+/fl^ mice injected with AAV-PHP.eB carrying tdTomato crRNAs or NT crRNA at 12 dpi. **f** Schematic illustration of delivering AAV-PHP.eB carrying tdTomato crRNAs or NT crRNA into ^LSL^RfxCas13d^+/fl^Ai14^+/fl^ and ^LSL^RfxCas13d^fl/fl^ mice, and the possible outcomes. **g** Body weight change curve of ^LSL^RfxCas13d^fl/fl^ and ^LSL^RfxCas13d^+/fl^Ai14^+/fl^ mice after injection of AAV-PHP.eB carrying tdTomato crRNAs (*n*=4) or NT crRNA (*n*=4). **h** Survival curve of ^LSL^RfxCas13d^fl/fl^ and ^LSL^RfxCas13d^+/fl^Ai14^+/fl^ mice after injection of AAV-PHP.eB carrying tdTomato crRNAs (*n*=4) or NT crRNA (*n*=4). **i** Body weight change curve of Ai14 (*n*=4) and ^LSL^RfxCas13d^+/fl^Ai14^+/fl^ (*n*=3) mice after injection of AAV-PHP.eB carrying tdTomato crRNA 7. **j** Survival curve of Ai14 (*n*=4) and ^LSL^RfxCas13d^+/fl^Ai14^+/fl^ (*n*=3) mice after injection of AAV-PHP.eB carrying tdTomato crRNA 7. **a** One-way ANOVA with Dunn’s multiple comparisons test. **g**, **j** Two-tail unpaired *t* test. **d**, **h**, **j** Log-rank test. Significance levels are noted as *P < 0.05, **P < 0.01, ***P < 0.001, ****P < 0.0001, or ns (*P* > 0.05). All values are presented as mean ± SEM

Firstly, Ai14 (*Rosa26*-CAG-LSL-tdTomato-WPRE) reporter mice were introduced and crossed with ^LSL^RfxCas13d^fl/fl^ mice to generate ^LSL^RfxCas13d^+/fl^Ai14^+/fl^ mice [38] (Additional file 1: Fig. S6). We designed seven crRNAs targeting tdTomato and tested their knockdown efficiency in N2a cells stabling expressing tdTomato by RT-qPCR. Among these crRNAs, crRNA 4 and 7 significantly damped the expression of tdTomato (Fig. 3a). Transcriptome analysis showed that tdTomato was specifically knocked down in cells transfected with tdTomato crRNA 4 or 7 (Fig. 3b and Additional file 2: Table S1). Then, we knocked down tdTomato in ^LSL^RfxCas13d^+/fl^Ai14^+/fl^ mice in the same way as knocking down *Sik3-S*. Results showed that mice injected with AAV-PHP.eB carrying tdTomato crRNA 7 began to lose weight at ~12 dpi and died at ~15 dpi, mice in the other groups behaved normally and survived (Fig. 3c,d). Brain lysates showed that tdTomato expression was lower in tdTomato crRNA 7 group than NT crRNA or tdTomato crRNA 4 group (Fig. 3e).

Next, we simultaneously injected AAV-PHP.eB carrying tdTomato crRNA 4, 7, or NT crRNA into ^LSL^RfxCas13d^fl/fl^ and ^LSL^RfxCas13d^+/fl^Ai14^+/fl^ mice. Theoretically, if RfxCas13d-mediated mouse death was caused by off-target effects, both ^LSL^RfxCas13d^fl/fl^ and ^LSL^RfxCas13d^+/fl^Ai14^+/fl^ mice injected with AAV-PHP.eB carrying tdTomato crRNA 7 would die, whereas, if RfxCas13d-mediated mouse death was not caused by off-target effects, only ^LSL^RfxCas13d^+/fl^Ai14^+/fl^ mice injected with AAV-PHP.eB carrying tdTomato crRNA 7 would die (Fig. 3f). Results showed that only ^LSL^RfxCas13d^+/fl^Ai14^+/fl^ mice injected with AAV-PHP.eB carrying tdTomato crRNA 7 began to lose body weight at ~12 dpi and died at ~15 dpi, mice in the other groups behaved normally (Fig. 3g, h). These data demonstrated that RfxCas13d-mediated mouse death was not caused by off-target effects and suggested that RfxCas13d and tdTomato crRNA 7 were insufficient to cause mouse death, and tdTomato expression was also required. Moreover, since tdTomato is a foreign gene and has no function in brain neurons, this result further supports that RfxCas13d-mediated lethality had nothing to do with the loss of target gene function.

Since crRNAs have been reported to knock down genes even in the absence of Cas13 protein [39], we also tested whether the mouse death observed was mediated by tdTomato crRNA 7 alone independent of RfxCas13d protein. We simultaneously injected AAV-PHP.eB carrying Cre and tdTomato crRNA 7 into Ai14 and ^LSL^RfxCas13d^+/fl^Ai14^+/fl^ mice. Results showed that ^LSL^RfxCas13d^+/fl^Ai14^+/fl^ mice began to lose body weight at ~12 dpi and died at ~15 dpi, while Ai14 mice behaved normally (Fig. 3i, j). These data demonstrated that tdTomato crRNA 7 alone was insufficient to cause mouse death, and RfxCas13d protein was also required.

Taken together, mice only died when RfxCas13d significantly knocked down the target gene (tdTomato) guided by the targeting crRNA (tdTomato crRNA 7). The expression of any two of RfxCas13d, tdTomato, and tdTomato crRNA 7 was insufficient to cause death in mice, and only simultaneous expression of all three caused death in mice. And these three components were also required to activate the collateral activity of RfxCas13d. Therefore, these data suggested that RfxCas13d-mediated lethality may result from the collateral activity of RfxCas13d rather than off-target effects.

### The collateral activity of RfxCas13d was positively correlated with the abundance of target RNA in mammalian cells

**Fig. 4:**
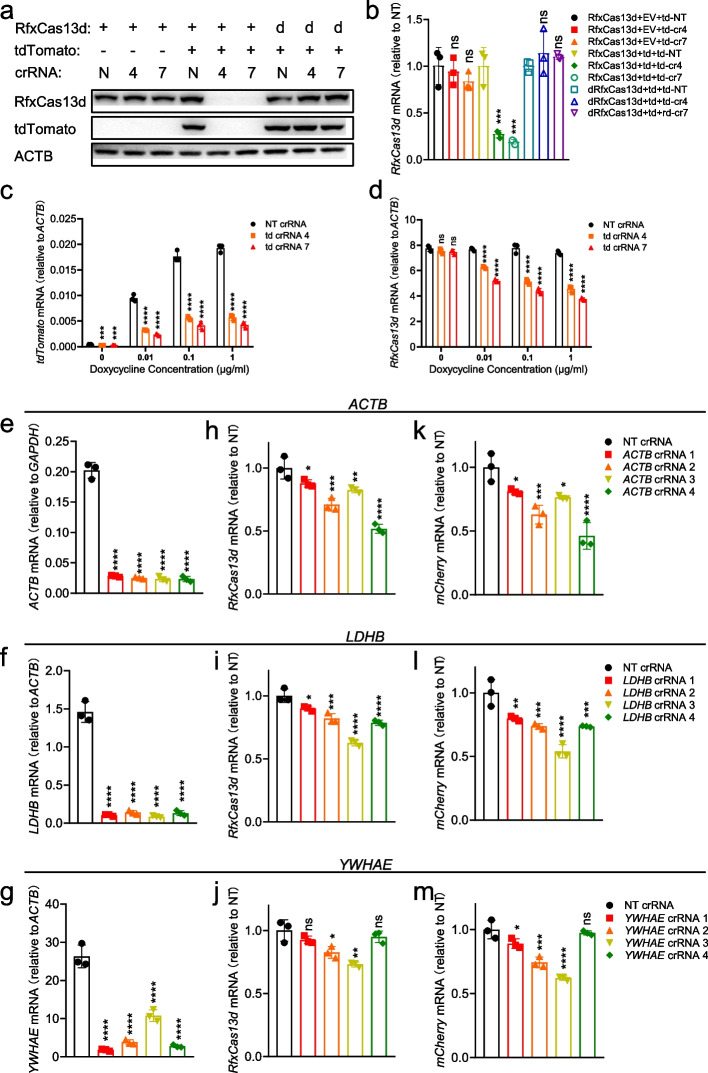
The collateral activity of RfxCas13d was positively correlated with the abundance of target RNA in mammalian cells. **a** Western blot to measure the expression level of RfxCas13d and tdTomato 24 h after transfection plasmids encoding RfxCas13d, tdTomato, and crRNAs into HEK293T cells. 3F-tdTomato means tdTomato with 3× Flag tag at N-terminal. **b** RT-qPCR to measure the RNA level of RfxCas13d in **a**. td represents tdTomato. EV represents empty vector. **c**, **d** RT-qPCR to measure the mRNA level of tdTomato (**c**) and RfxCas13d (**d**) 24 h after transfection of plasmids encoding RfxCas13d and crRNA into the inducible-expressing tdTomato HEK293T cells. The cells were pretreated with different concentrations of doxycycline for 16 h. **e**–**g** RT-qPCR to measure the knockdown efficiency of ACTB, LDHB, and YEHAE crRNAs in HEK293T cells (*n*=3). **h**–**m** RT-qPCR to measure the mRNA levels of RfxCas13d (**h**–**j**) and mCherry (**k**–**m**) in the HEK293T cells 24 h after transfection of plasmids respectively encoding RfxCas13d, mCherry, and crRNA. **b**–**m** One-way ANOVA with Dunn’s multiple comparisons test. Significance levels are noted as *P < 0.05, **P < 0.01, ***P < 0.001, ****P < 0.0001, or ns (*P* > 0.05). All values are presented as mean ± SEM

Up to now, it is still controversial whether Cas13 exhibits collateral activity in mammalian cells. To evaluate the collateral activity of RfxCas13d in mammalian cells, we transiently transfected plasmids encoding RfxCas13d/dRfxCas13d (catalytically dead RfxCas13d), tdTomato/SIK3-S, and targeting crRNAs/NT crRNA into HEK293T cells. Results showed that the protein and RNA level of RfxCas13d decreased, when it knocked down tdTomato/SIK3-S under guidance of tdTomato/Sik3-S crRNAs not NT crRNA (Fig. 4a, b and Additional file 1: Fig. S7a-b). However, this phenomenon would not occur when there was no target gene expression or using dRfxCas13d (Fig. 4a, b and Additional file 1: Fig. S7a-b). This suggested that the collateral activity of RfxCas13d was activated to cleave its own mRNA when RfxCas13d-crRNA complex bound and cleaved tdTomato/SIK3-S mRNA. Interestingly, changes in protein levels were more obvious than changes in RNA levels (this will be explained later). LwaCas13a and PspCas13b also exhibited similar characteristics (Additional file 1: Fig. S7c-d).

Interestingly, we could easily observe the collateral activity of RfxCas13d upon targeting overexpressed genes in HEK293T cells, but the previous study did not detect it upon targeting endogenous genes in the same cells [12]. Similarly, Yang group also found that LwaCas13a, RfxCas13d, and Cas13X.1 exhibited weak collateral activity when targeting transiently overexpressing mCherry, but not endogenous genes, using EGFP stably expressed in HEK293T as the indicator of collateral effects [14], which gives us a hint that the collateral activity of Cas13 may relate with the abundance of target RNA. Besides, in bacteria, Cas13-induced dormancy requires target RNA levels to exceed an expression threshold [40]. And in vitro experiments proved that collateral activity of Cas13 is positively correlated with the abundance of target RNA [6, 9]. To verify whether this correlation also exists in mammalian cells, a HEK293T cell line inducibly expressing tdTomato was constructed leveraging the tetracycline-controlled Tet-On inducible expression system, and then transfected with plasmids encoding RfxCas13d and crRNAs under different concentration doxycycline treatment. The mRNA level of tdTomato increased with the dose of doxycycline (Fig. 4c). Compared with NT crRNA, the mRNA level of tdTomato was significantly knocked down by RfxCas13d with targeting crRNAs, and the mRNA level of RfxCas13d was also downregulated at the same time (Fig. 4c, d). RfxCas13d was negatively correlated with tdTomato at the expression level under co-transfection of RfxCas13d with targeting crRNAs instead of NT crRNA (Fig. 4c, d). These data indicated that the collateral activity of RfxCas13d was triggered and positively correlated with the abundance of target RNA in mammalian cells when targeting exogenous genes, which is consistent with the findings of recent studies [22–25].

Next, we determined whether the collateral activity of RfxCas13d occurs when targeting endogenous genes in mammalian cells. We noticed that these endogenous genes previously used as targets are low in abundance [12, 14]. It is possible that collateral activity may have been activated, but it was too weak to be detected. Therefore, we here selected several highly expressed genes as targets and designed four crRNAs for each gene. Through RT-qPCR, we confirmed that all crRNAs can effectively knock down the target genes (Fig. 4e–g and Additional file 1: Fig. S7e-h). Then, we transfected plasmids respectively encoding RfxCas13d, crRNA, and mCherry into HEK293T cells. Changes in the expression levels of RfxCas13d and mCherry were used as indicators of collateral activity. Results showed that RfxCas13d and mCherry were downregulated when targeting these highly expressed genes, indicating that collateral activity was activated (Fig. 4h–m and Additional file 1: Fig. S7i-p).

Taken together, these data demonstrated that RfxCas13d exhibited collateral activity in mammalian cells, which is positively correlated with the abundance of target RNA. And recent studies have also reached the same conclusion as ours [22–25].

### The collateral activity of RfxCas13d cleaved 28s rRNA into two fragments, leading to translation attenuation and activation of ZAKα-JNK/p38-IEG pathway

**Fig. 5:**
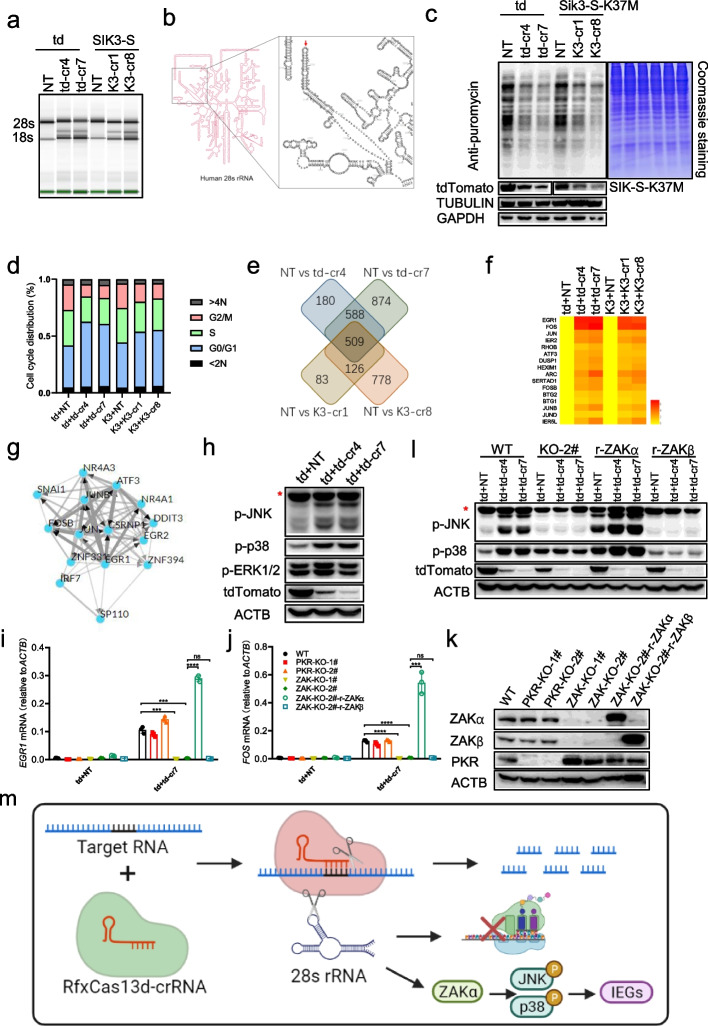
The collateral activity of RfxCas13d cleaved 28s rRNA into two fragments, leading to translation attenuation and activation of ZAKα-JNK/p38-IEG pathway. **a** Quality control of total RNA of HEK293T-RfxCas13d cells transfected with plasmids encoding tdTomato/SIK3-S and corresponding crRNAs (as shown in Additional file 1: Fig. S8a) by Agilent 2200 Bioanalyzer. td: tdTomato; td-cr4/7: tdTomato crRNA 4/7; K3-cr1/8: *Sik3-S* crRNA 1/8. **b** Schematic illustration of the cleavage site of RfxCas13d on human 28s rRNA. **c** SUnSET essay to measure the protein translation rate of HEK293T-RfxCas13d cells 24 h after transfection of plasmids encoding tdTomato/SIK3-S-K37M and corresponding crRNAs. **d** Statistical diagram of cell cycle distribution in Additional file 1: Fig. S9b. **e** Schematic illustration of 509 common DEGs (*P* adj < 0.05 and change fold ≥2) from four sets of comparisons. **f** Heatmap representing the log2 fold-change values of IEGs in targeting crRNA-transfected groups vs. NT crRNA-transfected groups. **g** Transcription factor enrichment analysis using ChEA3. There are Top 15 transcription factors. **h** Western blot to measure the phosphorylation level of p38, JNK, and ERK in HEK293T-RfxCas13d 24 h after transfection of plasmids encoding tdTomato and tdTomato crRNAs or NT crRNA. Red asterisk represents non-specific band. **i**, **j** RT-qPCR to measure the RNA level of EGR1 and FOS in HEK293T-RfxCas13d cells 24 h after transfection of plasmids encoding tdTomato and tdTomato crRNAs or NT crRNA. **k** Western blot to measure the expression level of ZAKα, ZAKβ, and PKR in indicated cells. PKR-KO-1/2#: two strains of PKR knockout HEK293T-RfxCas13d cells; ZAK-KO-1/2#: two strains of ZAK knockout HEK293T-RfxCas13d cells; ZAK-KO-2#-r-ZAKα: re-expression of ZAKα in ZAK-KO-2#; ZAK-KO-2#-r-ZAKβ: re-expression of ZAKβ in ZAK-KO-2#. **l** Western blot to measure the phosphorylation level of p38, JNK 24 h after transfection of plasmids encoding RfxCas13d and tdTomato crRNA 7 or NT crRNA into indicated cells. Red asterisk represents non-specific band. KO-2#: ZAK-KO-2#; r-ZAKα: ZAK-KO-2#-r-ZAKα; r-ZAKβ: ZAK-KO-2#-r-ZAKβ. **m** Schematic illustration that the collateral activity of RfxCas13d cleaves 28s rRNA into two fragments, leading to translation attenuation and activation of ZAKα-p38/JNK-IEGs pathway. **i**, **j** Two-tail unpaired *t* test. Significance levels are noted as ***P < 0.001, ****P < 0.0001, or ns (*P* > 0.05). All values are presented as mean ± SEM

Although it was confirmed that the collateral activity of RfxCas13d existed in mammalian cells, it remains unknown whether and how this activity affects the biological process of cells. To this end, we constructed a HEK293T cell line stably expressing RfxCas13d (HEK293T-RfxCas13d) and then transfected with plasmids encoding target genes and corresponding crRNAs (Additional file 1: Fig. S8a). In this way, RfxCas13d was fully expressed before the collateral activity was induced, which circumvents the collateral cleavage effect of RfxCas13d on its own mRNA (Additional file 1: Fig. S8b). Thus, more RfxCas13d protein and the secondary induced collateral activity could be preserved than co-transfection. Cells were harvested 24 h post-transfection for cell cycle distribution analysis, total RNA integrity analysis, and RNA-seq analysis (Additional file 1: Fig. S8a). Analysis of total RNA integrity showed that not only 28s and 18s rRNA, but also two additional bands were detected when co-transfecting of target genes with corresponding targeting crRNAs instead of NT crRNA into HEK293T-RfxCas13d cells (Fig. 5a). The same phenomenon occurred when targeting highly expressed endogenous genes (Additional file 1: Fig. S8c). In terms of size, these two additional bands looked like the products of 28s rRNA being cleaved. To test this, we did oligonucleotide extension assay to map cleavage sites (Additional file 1: Fig. S8d). PCR and Sanger sequencing revealed that 28s rRNA was cut into two fragments, one fragment of ~2100nt and the other segment of ~2800nt (Additional file 1: Fig. S8e-f). Noticeably, several sequencing results detected until ~2187nt of 28s rRNA (marked by blue color), and one sequencing result revealed that a poly-A tail was added to 2187nt of 28s rRNA (marked by brown color) (Additional file 1: Fig. S8f). There is “UU” behind 2187nt of the complete 28s rRNA (marked by red color) (Additional file 1: Fig. S8f). And we proved that the collateral activity of RfxCas13d prefers to cleave poly-U in vitro, which is consistent with the previous study [41] (Additional file 1: Fig. S8g-h). Therefore, this “UU” site (2188–2189nt) is likely to be the cleavage site by RfxCas13d on 28s rRNA (Fig. 5b). Those slightly shorter fragments may be caused by post-cleavage degradation. Interestingly, why did the collateral activity of RfxCas13d cleave 28s rRNA but not 18s rRNA? Theoretically, the abundance of 18s rRNA is also high, and there are also “UU” sites on it that can be cleaved. Besides, why did the collateral activity of RfxCas13d cut 28s rRNA at this “UU” site, not others? We speculated that it may be due to the structure of RNA and RNA binding proteins (RBPs) that protect rRNAs from being cut. And only “UU” site (2188–2189nt) on 28s rRNA is accessible to the collateral activity of RfxCas13d in vivo. To test our speculation, we extracted total RNA from HEK293T cells and then reconstituted the collateral activity of RfxCas13d in vitro and found that 28s and 18s rRNA were cleaved into multiple fragments (Additional file 1: Fig. S8i). These results suggested that RNA structure and RBPs were involved in protecting RNA from the collateral activity of RfxCas13d.

28s rRNA is a key component of the ribosome. To determine whether the translation function of the ribosome was affected due to 28s rRNA breakage, SUnSET assay was employed to monitor protein synthesis. To avoid the impact of SIK3-S enzyme activity on translation, we used kinase dead SIK3-S (SIK3-S-K37M) instead of WT SIK3-S (Additional file 1: Fig. S9a). Results showed that protein synthesis was attenuated when target genes were co-transfected with targeting crRNA, not NT crRNA (Fig. 5c). These data suggested that the collateral activity of RfxCas13d cleaved 28s rRNA into two fragments, thereby affecting the translation function of the ribosome. This may explain why in Fig. 4a, changes in protein levels of RfxCas13d were more obvious than changes in RNA levels (Additional file 1: Fig. S8b). Cell cycle distribution analysis showed that co-transfection of target genes with targeting crRNAs, but not NT crRNA leaded to cell cycle arrest at G1 phase (Fig. 5d and Additional file 1: Fig. S9b). This may result from impaired translation of protein.

RNA-seq analysis showed that there were 509 common differentially expressed genes (DEGs) (*P* adj < 0.05 and change fold ≥2) from four sets of comparisons (Fig. 5e and Additional file 3: Table S2). Interestingly, these were all upregulated genes compared with NT crRNA (Additional file 3: Table S2). Among these genes, we noticed that multiple genes with obvious difference belong to IEGs (Fig. 5f). Besides, transcription factor enrichment analysis of 509 genes showed that multiple enriched transcription factors mediate the expression of IEGs, including JUNB, FOSB, JUN, EGR1, EGR2, ATF3, NR4A3, NR4A1, and CSRNP1 (Fig. 5g). IEGs are activated transiently and rapidly in response to various cellular stimuli and several pathways have been reported to regulate the activation of IEGs, such as the RhoA-actin and the ERK, JNK, and p38 MAPK pathways [42]. We used inhibitors of these pathways to block IEG expression and found that IEG expression can be blocked by p38 and JNK inhibitors but not MEK1/2 or RhoA/C inhibitors (Additional file 1: Fig. S9c-d). The combination of p38 and JNK inhibitors worked better (Additional file 1: Fig. S9c-d). Consistently, western blot revealed increased phosphorylation of p38 and JNK, but not ERK1/2 (Fig. 5h), demonstrating that JNK and p38 pathways were responsible for the expression of IEGs. Previous studies reported that ZAKα, the long isoform of ZAK, senses ribotoxic stress caused by rRNA damage or ribosome impairment, and then activates p38 and JNK pathways [43]. We speculated that ZAKα may sense 28s rRNA breakage caused by RfxCas13d and activate JNK and p38 pathways. To test this, we firstly used two ZAK inhibitors (6p [44] and HY180) to block IEG expression. IEG expression can be inhibited by both inhibitors in a dose-dependent manner (Additional file 1: Fig. S9e-f). Then, we knocked out ZAK in HEK293T-RfxCas13d cells and found that IEG expression was blocked in ZAK knockout cells not PKR (another ribotoxic stress sensor) knockout cells (Fig. 5i–k). Besides, re-expression of ZAKα but not ZAKβ (the short isoform of ZAK) in ZAK knockout cells can rescue the expression of IEGs (Fig. 5i–k). Consistently, western blot demonstrated that phosphorylation of p38 and JNK was blocked in ZAK knockout cell and can be rescued by re-expression of ZAKα but not ZAKβ (Fig. 5l). These data proved that ZAKα sensed 28s rRNA breakage caused by RfxCas13d and mediated phosphorylation of p38 and JNK, then activating IEG expression.

Moreover, we found that the collateral activity of RfxCas13d caused upregulation of IEGs in HEK293T cells when targeting highly expressed endogenous genes, but not targeting low-expressed endogenous genes (Additional file 1: Fig. S10). This was consistent with previous studies that no collateral activity of RfxCas13d was detected upon targeting lowly expressed genes in HEK293T cells [3, 7, 12, 14], and again indicated that the collateral activity of RfxCas13d is correlated with target RNA abundance. We further tested the collateral activity of RfxCas13d in other mammalian cell lines and found that RfxCas13d also exhibited collateral activity in Hela, MBA-MD-231, and HCT116 cells, resulting in 28s rRNA breakage, impaired translation and upregulation of IEGs (Additional file 1: Fig. S11).

Taken together, these data demonstrated that the collateral activity of RfxCas13d cleaves 28s rRNA into two fragments, leading to translation attenuation and activation of the ZAKα-JNK/p38-IEG pathway (Fig. 5m).

## Discussion

Since Cas13-mediated gene silencing does not change genomic DNA and is reversible, Cas13 is considered safer than Cas9 and is a promising therapy for cancers, metabolic diseases, neurodegenerative diseases, and virus infections [15, 17, 39, 45]. Although collateral activity of Cas13 was obviously detected in bacteria and in vitro, it was initially not observed in mammalian cells. However, our results confirmed that RfxCas13d exhibited collateral activity in mammalian cells and that knocking down gene expression using RfxCas13d in the adult brain neurons caused death of mice. Therefore, it is needed to re-evaluate the safety of Cas13 and the research conclusions obtained using Cas13.

Previous studies have proved that RfxCas13d can be used to knock down endogenous genes in vivo with no reported side effects in the liver, brain, and eyes [15–17]. However, we found that mice died when knocking down target genes in the adult mouse neurons using RfxCas13d. The reasons for the conflict may be as follows: (1) RfxCas13d may also exhibit in vivo toxicity in previous studies but was not noticed or detected. For example, a recent study reported that using AAV8 to deliver RfxCas13d and crRNAs targeting *Pcsk9* into mouse liver as the previous study did [15], caused liver damage [22]. (2) The AAV subtype and injection method we used determined that RfxCas13d would be expressed more widely in the central nervous system in our study than in the previous study [17]. Therefore, more neurons were affected by the collateral activity of RfxCas13d, resulting in mouse death. Moreover, compared with the liver and eyes, neurons in the brain are more fragile and less prone to regeneration and are more indispensable for the survival of mice. (3) Differences in the expression levels of target transcripts may affect the extent of collateral activity (analogous to the in vitro effect). (4) Differences in promoter strength, vector dosing, and serotype may cause the different strength of RfxCas13d expression.

A recent study showed that overexpression of RfxCas13d resulted in death in mice after birth and caused more rapid death together with targeting crRNA, suggesting that both overexpression of RfxCas13d and its collateral activity are toxic in vivo [22]. It is worth noting that they inserted CAG-RfxCas13d cassette into the mouse genome using the PiggyBac transposon system, resulting in over 50 copies of transgenes per cell [22], whereas we inserted CAG-LSL-RfxCas13d into the mouse genome by homologous recombination using CRISPR/Cas9 technology, resulting in only two copies of transgenes per cell. The expression levels of RfxCas13d vary widely between our mice and theirs, and the expression of RfxCas13d in our mice is only released in cells in the presence of Cre recombinase. We agree with their findings, but our experimental results showed that the RfxCas13d-mediated mouse death we observed was dominantly caused by the collateral activity of RfxCas13d rather than the expression of RfxCas13d. The reasons are as follows: (1) We obtained the mouse line constitutively expressing RfxCas13d by mating ^LSL^RfxCas13d^fl/fl^ mice with the β-actin-Cre transgenic mice (FVB/N-*Tmem*163^Tg(ACTB-cre)2Mrt^/J; Strain #:003376; This mouse line express Cre recombinase directed by the human beta actin gene promoter). And these mice behaved as normally as WT mice (data not shown). (2) In Figs. 1, 2, and 3, we retro-orbitally injected ^LSL^RfxCas13d^fl/fl^ or ^LSL^RfxCas13d^+/fl^Ai14^+/fl^ mice with AAV-PHP.eB carrying hSYN-driven Cre and U6-driven crRNA, targeting crRNAs as the experimental groups and NT crRNA as the control group. In the control group, the expression of RfxCas13d was also released in neurons by hSYN-driven Cre recombinase, but no death of mice was observed. (3) Furthermore, in Fig. 3, we demonstrated that expression of any two of RfxCas13d, tdTomato, and tdTomato crRNA 7 was insufficient to cause death in mice, and only simultaneous expression of all three caused death in mice. These data suggested that mice may die from the collateral activity instead of RfxCas13d expression.

In this study, we have chosen two crRNAs for each gene to perform in vivo knockdown, but only 1 of 2 crRNAs caused death in mice. This phenomenon may be caused by different collateral activities triggered by different crRNAs in vivo. The lethal crRNAs may elicit strong collateral activity, which exceeded the tolerance threshold of mice, resulting in mouse death, whereas the non-lethal crRNAs may elicit collateral activity below the tolerance threshold of mice, so no mouse death was observed.

Whether Cas13 has collateral activity in mammalian cells has previously been debated. In this study, we confirmed that RfxCas13d exhibited collateral activity in mammalian cells, which is positively correlated with the abundance of target RNAs. Consistently, several recently published and preprinted studies have also reached the conclusions as ours [22–25]. These findings partly explain the conflict about the collateral activity of Cas13. It remains to be further investigated whether RfxCas13d exhibits detectable collateral activity upon targeting lowly expressed genes. And we believe that RfxCas13d also cleaves RNAs other than 28s rRNA, although we have not yet identified them. Among the 509 common DEGs (Fig. 5e), the upregulation of other genes except IEGs is also worthy of further investigation. Therefore, it is urgently needed to develop a highly sensitive high-throughput sequencing technology to study all the cleavage substrates and sites of the collateral activity, which will help to gain insights into the mechanism of collateral activity and the optimization of Cas13.

To determine whether RfxCas13d exhibited collateral activity in N2a when targeting *Sik3-S*, *Map2*, *Mapt*, and *Rbfox3*, we performed RNA-seq using RfxCas13d/dRfxCas13d with or without crRNA in N2a cells and identified DEGs (*P* adj < 0.05 and change fold ≥2) by comparing their transcriptome profiles, although these genes were expressed at low levels in N2a and lower than in brain (Additional file 1: Fig. S12a). Six DEGs were found in comparison between dRfxCas13d and RfxCas13d alone, and 194~1179 DEGs were identified in comparisons between dRfxCas13d with crRNA and RfxCas13d with crRNA (Additional file 1: Fig. S12b and Additional file 4: Table S3). This indicated that RfxCas13d alone only had little effect on the transcriptome, whereas RfxCas13d equipped with crRNA did. Through analysis, we identified 185 common DEGs from seven sets of comparisons (Groups 3–9) between dRfxCas13d with targeting crRNA and RfxCas13d with targeting crRNA (Additional file 4: Table S3). These DEGs were spacer sequence-independent, and therefore were thought to be associated with collateral activity. GO term analysis showed that these genes were significantly enriched in nucleosome assembly (cluster 1) and ribosome (cluster 2) (Additional file 1: Fig. S12c). And these nucleosome and ribosome-related genes were upregulated in all seven sets of comparisons (Additional file 1: Fig. S12d). Consistently, Tong et al. recently reported that upregulated genes induced by RfxCas13d targeting RPL4 and PPIA can be enriched in nucleosome assembly [22]. Moreover, multiple histone genes and a few ribosome-related genes were found in the DEGs caused by RfxCas13d in HEK293T and U87 cells [21]. Besides, we also compared the transcriptomes between dRfxCas13d with NT crRNA and dRfxCas13d with targeting crRNA, and no or few (3 and 17) DEGs were found, indicating that crRNA alone had little effect on the transcriptome (Additional file 1: Fig. S12d and Additional file 4: Table S3). These data suggested that these nucleosome and ribosome-related genes were associated with collateral activity, but how collateral activity affected the expression of these genes remains to be further investigated.

More recently, Tong et al. developed high-fidelity Cas13 variants that showed similar RNA knockdown activity to wild-type Cas13 but no detectable collateral damage in transgenic mice or adeno-associated-virus-mediated somatic cell targeting [22]. Kelley et al. introduced gRNA excision for negative-autoregulatory optimization (GENO) to attenuate Cas13d expression, which mitigated collateral effects in human cells while retaining modest on-target knockdown of CUG_n_ RNA [23]. With deep learning model interpretation, Wei et al. revealed specific sequence motifs at spacer position 15–24 along with favored secondary features for highly efficient guides. Furthermore, they identified DjCas13d, a highly efficient ortholog with minimal detectable cellular toxicity when targeting highly expressed transcripts in mammalian cells [46]. Those high-fidelity Cas13 variants or optimization with minimal collateral effects would be better choices for targeted degradation of RNAs in basic research and therapeutic applications. Moreover, the newly discovered Cas7-11 is also a promising candidate for transcriptome engineering [21].

## Conclusions

Here, we found that knocking down gene expression using RfxCas13d in the adult brain neurons caused death of mice, which may result from the collateral activity of RfxCas13d rather than the loss of target gene function or off-target effects. Mechanistically, we showed that RfxCas13d exhibited collateral activity in mammalian cells, which is positively correlated with the abundance of target RNA. The collateral activity of RfxCas13d could cleave 28s rRNA into two fragments, leading to translation attenuation and activation of the ZAKα-JNK/p38-IEG pathway. These findings provide new mechanistic insights into the collateral activity of RfxCas13d in mammalian cells and warn that the biosafety of CRISPR-Cas13 system needs further evaluation.

## Materials and methods

### Cell culture

HEK293T, N2a, MDA-MB-231, HCT116, and Hela cells were obtained from ATCC. Cells were cultured in DMEM supplemented with 10% FBS (Gibco) and 100 U/ml Penicillin-Streptomycin in a humidified incubator at 37 °C with 5% CO_2_. The 1640 culture medium was used for HCT116.

### Animals

All animal care and use adhered to the Guide for the Care and Use of Laboratory Animals of the Chinese Association for Laboratory Animal Science. All procedures of animal handling were approved by the Animal Care Committee of Peking University Health Science Center (permit number LA 2016240). ^LSL^RfxCas13d^fl/fl^ and Sik3-E5^flox^ mice on a C57BL/6J background were generated by the Transgenic Animal Center, NIBS, Beijing, China. Ai14 reporter mice were purchased from The Jackson Laboratory. Wild-type (WT) mice were purchased from Department of Laboratory Animal Science of Peking University Health Science Center, Beijing, China. Mice were kept and bred in pathogen-free conditions.

### Plasmid construction

Plasmids used in this study were prepared by standard molecular biology techniques and coding sequences entirely verified. Plasmids encoding LwaCas13a (#91902), PspCas13b (#103862), RfxCas13d (#109049), LwaCas13a crRNA backbone (#91906), RfxCas13d crRNA backbone (#109053), and Cas9&gRNA (#52961) were purchased from Addgene. The backbone expressing PspCas13b crRNA was engineered from #103862. For the construction of the plasmids encoding SIK3-S, SIK3-S-K37M, tdTomato, ZAKα, ZAKβ and NeuN, and AAV-PHP.eB backbone, the target fragments were amplified by PCR and ligated into the linearized corresponding vectors obtained by restriction enzyme (NEB) digestion via Gibson assembly (CL116-02, Biomed). For the construction of the plasmids encoding crRNAs, the primers for crRNA/gRNA were annealed and ligated into the linearized corresponding vectors cut by Bbs1 /BsmB1 (R0539S/R0739S, NEB) via T4 DNA ligase (M0202S, NEB). All plasmids were confirmed by sequencing. The sources, sequences, and application locations of all plasmids used were provided in Additional file 5: Table S4. In vitro-synthesized crRNAs were purchased from GenScript. All primers were order from Synbio Technologies or RuiBiotech. All sequencings were done in RuiBiotech.

### Reagents and antibodies

Polyethylenimine (PEI) (764582, Sigma-Aldrich) and jetPRIME (114-15, Polyplus) were used for transfection. Quenched fluorescent reporter RNA was purchased from General Biology. Inhibitors used in this study include the following: p38 inhibitor SB203580 (HY-10256, MCE); JNK inhibitor SP600125 (HY-12041, MCE); MEK1/2 inhibitor U0126 (HY-12031, MCE); RhoA/C inhibitor (S7719, Selleck). ZAK inhibitors 6P and HY180 are gifts from Prof. Xiaoyun Lu, Jinan University. Antibodies used in this study include the following: anti-Cre (Rabbit, 15036T, CST); anti-HA (Rat, 11867423001, Roche); anti-HA (Rabbit, H6908, Sigma-Aldrich); anti-HA (Mouse, self-made); anti-SIK3 (Rabbit, self-made); anti-β-Tubulin (Mouse, HC101, TransGen Biotech); anti-ACTB (Mouse, 60008-1-Ig, Proteintech); anti-GAPDH (Mouse, 60004-1-Ig, Proteintech); anti-NeuN (Rabbit, 26975-1-AP, Proteintech); anti-Tau (Rabbit, 10274-1-AP, Proteintech); anti-MAP2 (Rabbit, 17490-1-AP, Proteintech); anti-ZAK (Rabbit, 28761-1-AP, Proteintech); anti-PKR (Rabbit, 18244-1-AP, Proteintech); anti-p-p38 (Rabbit, 4511, CST); anti-p-JNK (Rabbit, 4370, CST); anti-p-ERK1/2 (Rabbit, ET1609-42, HUABIO); HRP-conjugated Affinipure Goat Anti-Rabbit IgG(H+L) (SA00001-2, Proteintech); HRP-conjugated Recombinant Rabbit Anti-Mouse IgG Kappa Light Chain (SA00001-1, Proteintech).

### Purification of RfxCas13d protein

The expression construct (The source and sequence of the plasmid was described in Additional file 5: Table S4) was transformed into BL21(DE3) cells and inoculated into a bacterial culture plate supplemented with 50 ug/ml kanamycin. A single colony was picked and incubated in 10 ml LB medium at 37°C for 12 h and then expanded to 1 L medium at a ratio of 1:100. Protein expression was induced overnight at 16°C with 0.5 mM Isopropyl-β-D-thiogalactopyranoside (IPTG) after OD600 nm reached 0.8. Cells were harvested by centrifugation (4000 rpm, 10 min) and resuspended in lysis buffer (20 mM Tris-HCl, 150 mM NaCl, 20 mM imidazole, 10 mM 2-mercaptoethanol and 1 mM phenylmethanesulfonylfluoride (PMSF), pH 8.0), and then lysed with an ultrasonic cell disruptor (300 W, work 5 s, off 3 s, 30 min). Cell debris was removed by centrifugation (18,000 rpm, 15 min), and the clear lysate containing recombinant RfxCas13d was first purified by affinity chromatography using Ni-NTA beads. The SUMO tag was removed by ULP1 protease digestion overnight at 4°C. The cleaved RfxCas13d was further purified by Superdex G-200 gel filtration chromatography (GE Healthcare Life Sciences). Proteins were collected and stored at −80°C until use.

### AAV-PHP.eB packaging, purification, and injection

AAV-PHP.eB was packaged in AAVpro 293T cells (632273, Clontech). PHP.eB (Addgene#103005), pHelper (240071-54, Agilent), and transfer plasmids were co-transfected to AAVpro 293T cells by PEI MAX (24765, Polysciences). Cells were harvested by cell lifter (70-2180, Biologix) 72 h post-transfection. The cell pellets were suspended in 1× Gradient Buffer (10 mM Tris-HCl pH=7.6, 150 mM NaCl, 10 mM MgCl_2_). Five repeated cycles of liquid nitrogen freezing, 37°C water bath thawing, and vortex were used to lyse cell. Then, ≥50 U/ml of Benzonase nuclease (E1014, Milipore) were added to cell lysates and incubated at 37°C for 30 min. Centrifuge the cell lysate at 21,130*g* for 30 min at 4°C and transfer the supernatant to a pre-build iodixanol (D1556, Optiprep) step gradients (15, 25, 40, and 58%) for ultracentrifugation purification. Vacuum centrifuge at 41,000 rpm, 4°C for 4 h, and the virus particles were in the layer of 40% iodixanol gradient. Purified virus were extracted from the 40% virus containing layer by needle and concentrated using Amicon filters (UFC801096, EMD) and formulated in sterile phosphate-buffered saline (PBS) supplemented with 0.01% Pluronic F68 (24040032, Gibco). Virus titers were determined by qPCR while a linearized AAV plasmid as a standard. 1×10^12^ vg/mouse AAV-PHP.eB were delivered into mice via retro-orbital injection.

### RNA extraction and reverse transcription quantitative real-time PCR (RT-qPCR)

Total RNA from cells or tissues were isolated using TRIzol reagent (DP424, TIANGEN) according to the instruction. One microgram RNA was reverse transcribed using HiScript II Q RT SuperMix (R223-01, Vazyme). Levels of these indicated genes were analyzed by qPCR amplified using SYBR Green (Q311, Vazyme). Data shown are the relative abundance of the indicated mRNA normalized to ACTB or GAPDH. The primers are listed in Additional file 6: Table S5.

### Measurement of crRNAs’ knockdown efficiency

Plasmids encoding RfxCas13d (addgene#109049) and crRNAs (addgene#109053) were transfected into N2a/HEK293T cells (Figs. 1b, 2d,e, 3a, 4d–f, and S4e-h). Forty-eight hours after transfection, GFP-positive cells were sorted and collected through Fluorescence-Activated Cell Sorting (FACS), and then were extracted for total RNA. Then, levels of indicated genes were measured by RT-qPCR. All crRNAs used in this paper are listed in Table S6.

### Cell cycle distribution analysis

Twenty-four hour after transfection of indicated plasmids, cells were washed and collected using PBS to get rid of serum proteins at centrifugation at 1200 rpm, 5 min. Pellets are resuspended using precooling 70% EtOH solution to fix cells at least 30 min at 4°C. The cells can remain in this solution for up to 1 week. EtOH/cell suspension was diluted with PBS, and was spun at 2000–2200 rpm for 10 min spin. Cells are much harder to pellet in EtOH. If EtOH is not diluted and the increased rate is not used, significant cell loss will be noticed. Wash cells three times using PBS and then stain cells using DAPI staining solution (C1005, Beyptime) for 30 min. Finally, cells were recorded by Fluorescence Activated Cell Sorting (FACS) and analyzed by FlowJo.

### Western blot

Cells were washed with PBS and lysed by incubation on ice for 10 min with RIPA lysis buffer (50 mM Tris, 150 mM NaCl, 0.1% SDS, 0.5% sodium deoxycholate, 1% Triton X-100, protease cocktail [C0001, Targetmol], and 1 mM PMSF). Additional phosphatase inhibitor cocktail (C0003, Targetmol) was used for western blot analysis of phosphorylated proteins. Brain tissue was firstly grinded in a mortar cooled on liquid nitrogen, and then lysed by incubation on ice for 30 min with RIPA lysis buffer. Supernatants were collected by centrifugation at 12,000 rpm for 10 min at 4°C and then mixed up with loading buffer and boiled for 10 min. Samples were resolved by SDS-PAGE and transferred to 0.22 μm nitrocellulose membrane (P-N66485, Pall). The membrane was blocked using skim milk for 30 min, then incubated overnight with primary antibodies, further incubated with the corresponding HRP-conjugated secondary antibodies, and finally detected by enhanced chemiluminescence. All uncropped western blot images are provided in Additional file 8.

### SUnSET assay

Cells were incubated with puromycin (2.5 μg/ml for HEK293T; 2 μg/ml for MDA-MB-231 and HCT116; 10 μg/ml for Hela) for 20 min and then washed with ice-cold PBS and lysed using RIPA lysis buffer. Equal quantity of cell lysates was submitted to western blot using anti-puromycin antibody to detect protein synthesis. Signals were normalized with probing GAPDH and TUBULIN (loading control).

### Construction of knockout cell lines

ZAK knockout and PKR knockout HEK293T cell lines were generated by a CRISPR-Cas9 system. Firstly, HEK293T cells were transfected with Lenti-V2 plasmids encoding Cas9 and gRNA (#52961). Culture medium was changed 6 h after transfection. Forty-eight hours after transfection, successfully transfected cells were selected in culture medium added puromycin. Then, monoclonal cells were obtained by limiting dilution. Finally, possible knockout cells of these monoclonal cells were validated by western blot and sequencing. All gRNAs used in this paper are listed in Additional file 7: Table S6.

### Construction of stable and inducible expression mammalian cell lines

For preparation of lentiviruses, HEK293T cells in 6-well plates were transfected with the lentiviral vector of interest (1800 ng), the lentiviral packaging plasmids psPAX2 (600 ng) and pMD2.G (600 ng) using 12 μl of PEI (1 mg/ml). Culture medium was changed 6 h after transfection. About 48 h after transfection, culture medium containing lentiviruses was collected and centrifugalized at 12,000 rpm for 10 min, and then filtered using a 0.22-μm filter. HEK293T, N2a, and U87 cells were then infected at ~50% confluency by lentiviruses for 48 h, followed by selection with puromycin or hygromycin for 7 days. More details on the construction of inducible expression cell lines were described in our previous study [47]. Monoclonal cells were obtained by limiting dilution and validated by western blot.

### Oligonucleotide extension assay

Total RNA was ligated with oligonucleotide adaptor 1 or 2 respectively using T4 RNA Ligase 1 (M0204S, NEB) following manual. Then RNA was purified by ethanol precipitation and then reverse transcribed using R1 or R2 (R312-02, Vazyme). cDNA was amplified by PCR using F1&R1 or F2&R2. PCR products were firstly analyzed by agarose gel electrophoresis. The desired bands were cut and purified by TIANgel Midi Purification Kit (DP209, Tiangen), then ligated into T vector (CT101-01, Transgen Biotech), and finally sequenced by Sanger sequencing.

The sequence of oligonucleotide adapters and primers:

- adaptor 1: 5-PO_4_-CTGTAGGCACCATCAATGGACCT-NH_2_-3 (DNA);
- adaptor 2: 5-NH_2_-CAGAAGGCACCAACAAAGGACC-OH-3 (RNA);
- F1: 5-ACCTGGGTATAGGGGCGAAAGAC-3 (DNA)
- R1: 5-AGGTCCATTGATGGTGCCTACAG-3 (DNA)
- F2: 5-CAGAAGGCACCAACAAAGGACC-3 (DNA)
- R2: 5-CCCTTAGAGCCAATCCTTATCCC-3 (DNA)

### Reconstitution of the collateral activity of RfxCas13d in vitro

To detect the collateral activity of RfxCas13d in vitro, we performed in vitro cleavage assay with 100 ng purified RfxCas13d protein, 100 ng synthesized tdTomato RNA, 100 ng crRNA, 2 μl RNase inhibitor (NEB), and 200 ng quenched fluorescent RNA reporters (6 nt poly-A/U/G/C), in 100 μl reaction buffer (40 mM Tris-HCl, 60 mM NaCl, 6 mM MgCl_2_, pH 7.6) [9]. Reactions were incubated at 37°C for 1 h and measured the fluorescence of RNA reporters with microplate reader. In Additional file 1: Fig. S8i, quenched fluorescent RNA reporters were replaced with total RNA extracted from HEK293T cells. Reactions were incubated at 37°C for 1 h and then RNA was purified by ethanol precipitation followed by quality control by Agilent 2200 Bioanalyzer.

### RNA denaturing gel electrophoresis

The process of making gel is as follows: weigh 0.5 g of agarose powder, add it to 36.5 ml of DEPC water, and heat to completely dissolve the agarose. After cooling slightly (60–70°C), add 5 ml of 10× MOPS Running Buffer (C516042-0001, Sangon Biotech), 8.5 ml of 37% formaldehyde. Then pour the gel in the glue tank, insert the comb, and place it horizontally for use after solidification. The process of adding the samples: mix the following reagents in a clean small centrifuge tube: 2 μl 10× MOPS Running buffer, 3.5 μl formaldehyde, 10 μl formamide (deionized), 4.5 μl RNA sample. Mix well, keep it at 60°C for 10 min, and cool quickly on ice. Add 3 μl of 10× loading buffer (B548318-0001, Sangon Biotech) and 0.5 μl of ethidium bromide, then mix well and add an appropriate amount to the sample well of the gel. Electrophoresis: turn on the electrophoresis instrument and stabilize the electrophoresis at 7.5 V/cm.

### Total RNA integrity analysis

Total RNA was extracted from cells and then sent to GENEWIZ company for quality control by Agilent 2200 Bioanalyzer.

### RNA-seq analysis

The sequencing data generated by Illumina Noveseq PE150 in fastq file format was filtered by FastQC (https://www.bioinformatics.babraham.ac.uk/projects/fastqc/) and Trim-Galore (https://www.bioinformatics.babraham.ac.uk/projects/trim_galore/) software for quality control. Then the mouse genome version mm10 and the human genome version hg38 were used as reference genome to align the clean data with Subread software (http://subread.sourceforge.net/). The gene count matrix was calculated by the featureCounts (http://subread.sourceforge.net/) program. Then the gene count data was normalized using the FPKM formula. The differentially expressed genes were analyzed by R package DESeq2 (https://bioconductor.org/packages/release/bioc/vignettes/DESeq2/inst/doc/DESeq2.html). Transcription factor enrichment analysis was conducted by ChEA3 (https://maayanlab.cloud/chea3/#top). The raw data and processed data were uploaded to the GEO Datasets (GSE193668, GSE222451, and GSE222461) [48–50].

### Statistical analysis

The statistical analysis was performed by Prism version 8 (GraphPad Software) including two-tailed unpaired *t* test, one-way ANOVA, and log-rank test. And the respective statistical test used for each figure is noted in the corresponding figure legends. Significance levels are noted as *P < 0.05, **P < 0.01, ***P < 0.001, ****P < 0.0001 or ns (*P* > 0.05). All values are represented as mean ± SEM.

## Supplementary Information

**Additional file 1.** Supplementary figures.

**Additional file 2: Table S1.** Transcriptome profiles of N2a cells transfected of RfxCas13d and crRNA, and DEGs identified through comparisons (Linked to Figs. 1, 2, and 3 and the answer to the first question of Reviewer 1# during first revision).

**Additional file 3: Table S2.** Transcriptome profiles of HEK293T-RfxCas13d cells transfected of target gene and crRNA, and 509 common DEGs identified through comparisons (Linked to Fig. 5).

**Additional file 4: Table S3.** Transcriptome profiles of N2a cells transfected of RfxCas13d/dRfxCas13d with or without crRNA, and DEGs identified through comparisons (Linked to Additional file 1: Fig. S12).

**Additional file 5: Table S4.** The sources, sequences and application locations of the plasmids used in this study.

**Additional file 6: Table S5.** The sequence of the primers used in this study.

**Additional file 7: Table S6.** The sequence of the crRNAs and gRNAs used in this study.

**Additional file 8.** Uncropped western blot images.

**Additional file 9: Table S7.** Transcriptome profiles of mouse brain tissue through bulk RNA-seq (Linked to additional file: Fig S12a and Response Fig. 1a of first revision).

**Additional file 10: Table S8.** Transcriptome profiles of HEK293T transfected of RfxCas13d, NeuN and crRNA (Linked to Response Fig. 8 of first revision).

**Additional file 11.** Review history.

## Data Availability

The datasets generated during the current study are available in the GEO Datasets (GSE193668, GSE222451, and GSE222461), including both raw and processed data [48–50].
