# Supplementary material for: The collateral activity of RfxCas13d can induce lethality in a RfxCas13d knock-in mouse model
Source: Genome Biol. 2023 Feb 1;24:20. doi: 10.1186/s13059-023-02860-w (PMC9893547; doi:10.1186/s13059-023-02860-w)
Supplement: Supplementary file 1 — Additional file 1. Supplementary figures. [file 13059_2023_2860_MOESM1_ESM.pdf]

Fig. S1

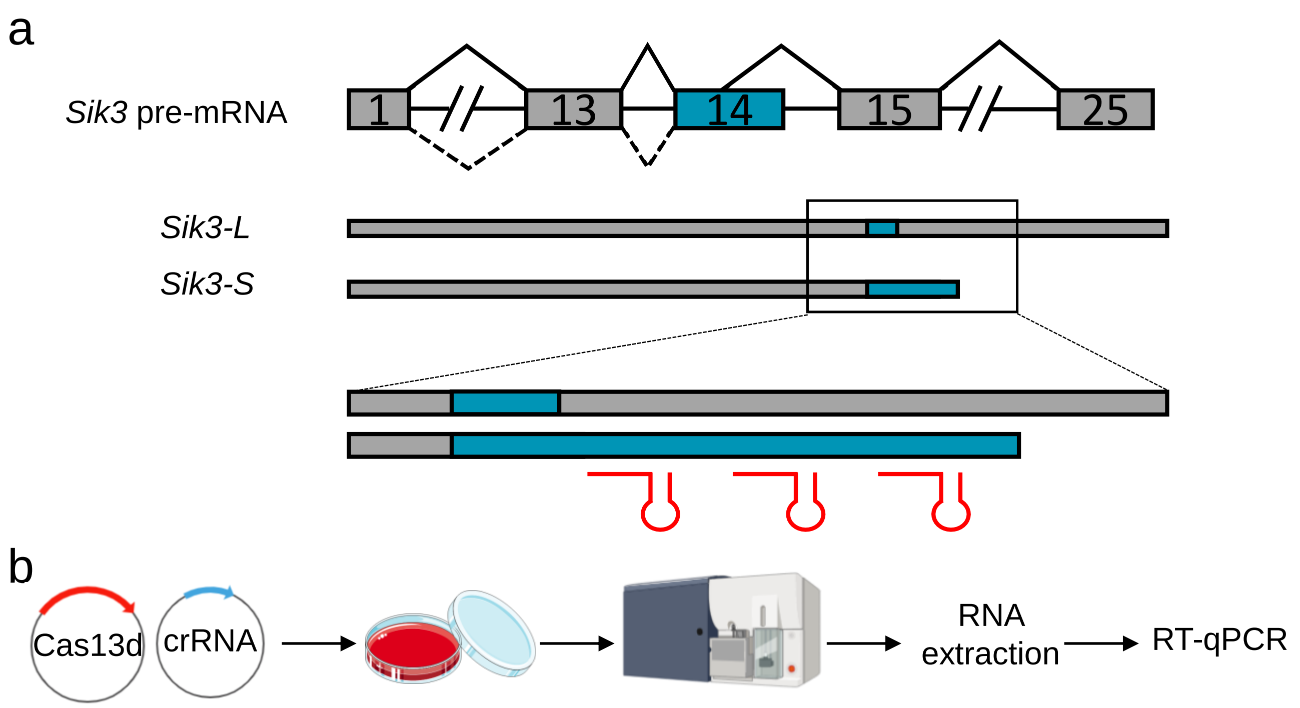
a
Sik3 pre-mRNA
1
13
14
15
25
Sik3-L
Sik3-S
b
Cas13d
crRNA
RNA extraction
RT-qPCR

Fig. S1 Designing crRNAs targeting *Sik3-S* to specifically knock down *Sik3-S* in tandem with RfxCas13d ((Linked to Fig. 1). **a.** Schematic illustration of alternative splicing process to generate *Sik3-L* and *Sik3-S* transcripts, and design of *Sik3-S* crRNAs. **b.** Workflow of measuring the knockdown efficiency of crRNAs in N2a or HEK293T cells.

Fig. S2

a

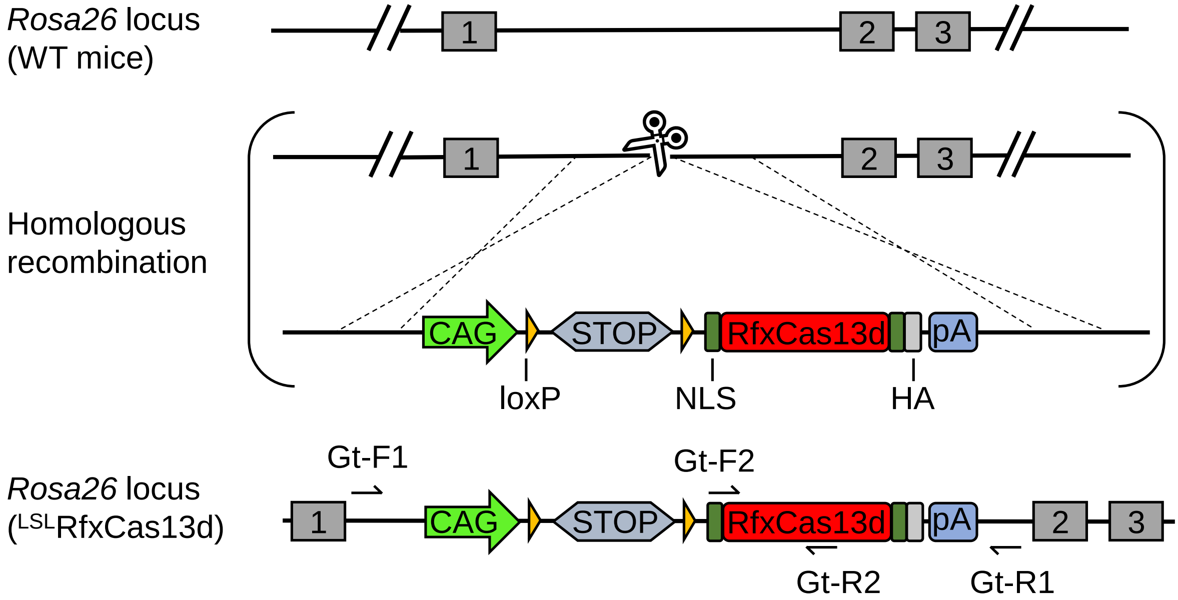

b

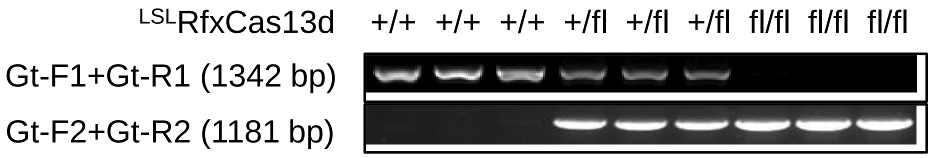

c

Embryos Microinjection Statistics			
Alleles	Embryo injected	Pups born	Positive founders
^{LSL}RfxCas13d	224	21	4 (19.0%)
^{LSL}RfxCas13d	100	13	2 (15.4%)

d

Statistical analysis of the deviations from Mendelian genetics									
	Observation			Expected			Chi-square value	df	P value
^{LSL}RfxCas13d	fl/fl	+/fl	+/+	fl/fl	+/fl	+/+			
Male	11	19	12	10.5	21	10.5	0.428571429	2	0.807117747
Female	13	24	16	13.25	26.5	13.25	0.811320755	2	0.6665365
Total	24	43	28	23.75	47.5	23.75	1.189473684	2	0.551707733

Fig. S2 The generation of ^{LSL}RfxCas13d mice (Linked to Fig. 1). **a.** Schematic illustration of the mechanism of generating ^{LSL}RfxCas13d mice. NLS: nuclear localization sequence; HA: hemagglutinin tag; CAG: CAG promoter; U6: U6 promoter; STOP: a tripartite transcriptional stop cassette. **b.** Genotyping of ^{LSL}RfxCas13d$^{fl/fl}$, ^{LSL}RfxCas13d$^{+/fl}$ and WT mice. **c.** Statistical table to show the results of twice embryo microinjections. **d.** Statistical analysis of the deviations from Mendelian genetics for six litters of mice (^{LSL}RfxCas13d$^{+/fl}$ x ^{LSL}RfxCas13d$^{+/fl}$). df: degree of freedom.

Fig. S3

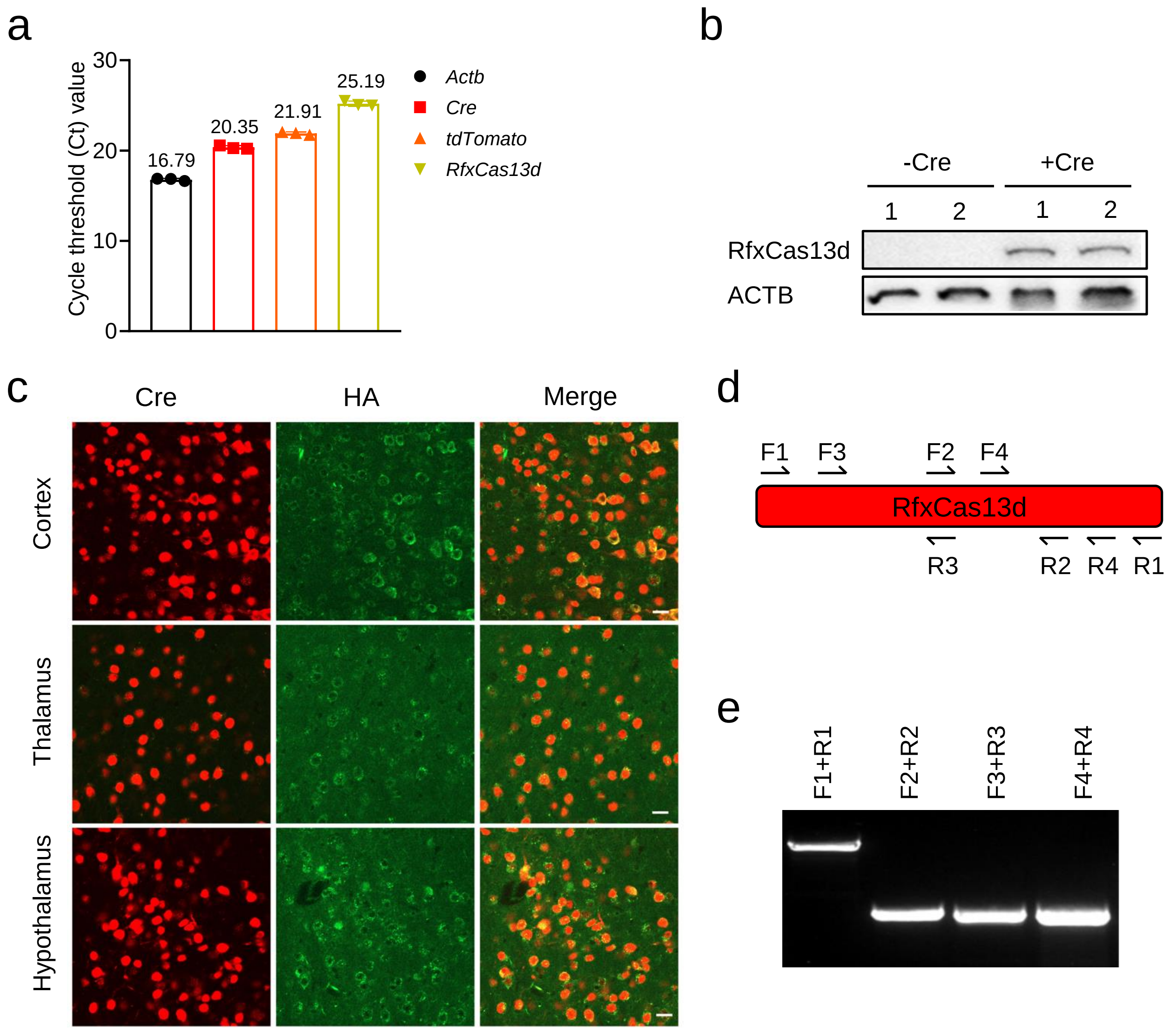
a
Cycle threshold (Ct) value
0
10
20
30
16.79
20.35
21.91
25.19
Actb
Cre
tdTomato
RfxCas13d
b
-Cre
+Cre
1
2
1
2
RfxCas13d
ACTB
c
Cre
HA
Merge
Cortex
Thalamus
Hypothalamus
d
F1
F3
F2
F4
RfxCas13d
R3
R2
R4
R1
e
F1+R1
F2+R2
F3+R3
F4+R4

Fig. S3 The expression of RfxCas13d can be released by hSYN-driven Cre in mouse brain (Linked to Fig. 1). **a.** RT-qPCR to measure the RNA level of *Actb*, *Cre*, *tdTomato* and *RfxCas13d* in the brain tissue from ^{LSL}RfxCas13d$^{+/fl}$Ai14$^{+/fl}$ mice two weeks after injection of AAV-PHP.eB carrying hSYN-driven Cre (n=3). The numbers above the histogram represent the average. **b.** Western blot to measure the protein level of RfxCas13d in the brain tissue from ^{LSL}RfxCas13d$^{+/fl}$Ai14$^{+/fl}$ mice two weeks after injection of AAV-PHP.eB carrying hSYN-driven Cre. **c.** Representative images showing coimmunostaining of Cre (1:1000, 15036T, CST) and RfxCas13d-HA (1:500, 11867423001, Roche) in the cortex, thalamus and hypothalamus (sagittal section) of ^{LSL}RfxCas13d$^{fl/fl}$ mice two weeks after injection of AAV-PHP.eB carrying hSYN-driven Cre (Flag tag). Scale bar: 20 μm. **d.** Schematic illustration of the design of primers for identifying the transcript variants of RfxCas13d in the brain tissue ^{LSL}RfxCas13d$^{+/fl}$Ai14$^{+/fl}$ mice two weeks after injection of AAV-PHP.eB carrying hSYN-driven Cre. **e.** Gel picture of PCR products using primers in **d**.

Fig. S4

a

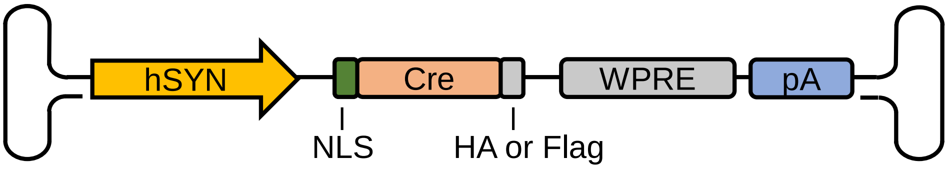

b

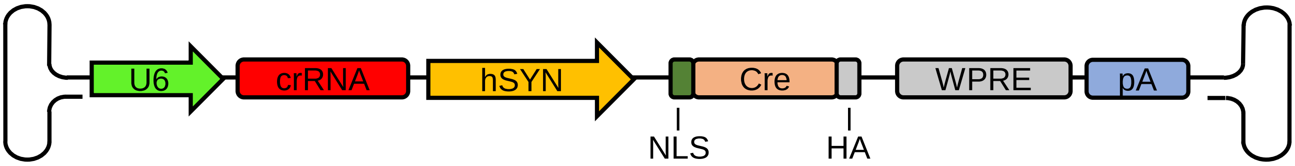

Fig. S4 Schematic structures of recombinant AAV-PHP.eB vector genomes (Linked to Fig. 1). **a.** Schematic illustration of AAV-PHP.eB plasmid used to deliver hSYN-driven Cre. Tag: HA/Flag. **b.** Schematic illustration of AAV-PHP.eB plasmid used to deliver U6-driven crRNA and hSYN-driven Cre. hSYN: human synapsin 1 gene promoter.

Fig. S5

a

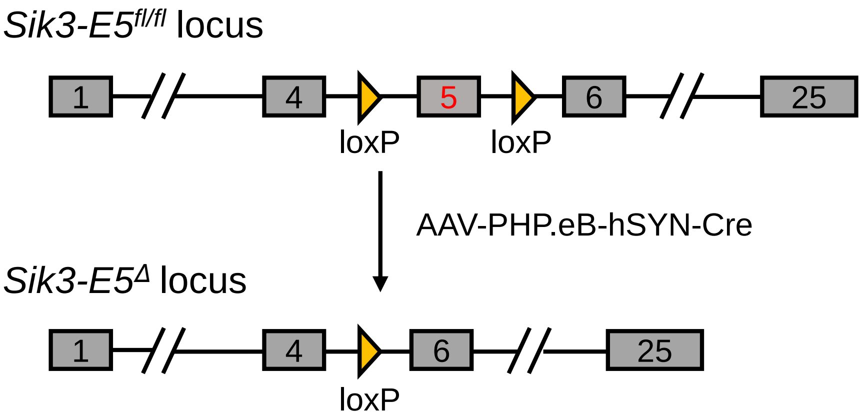
Sik3-E5fl/fl locus
1
4
5
6
25
loxP
loxP
AAV-PHP.eB-hSYN-Cre
Sik3-E5Δ locus
1
4
6
25
loxP

Fig. S5 Schematic illustration of knocking out Sik3 via the conventional Cre-loxP system (Linked to Fig. 2). **a.** Schematic illustration of knocking out Sik3 in Sik3-E5$^{fl/fl}$ mice by injection of AAV-PHP.eB carrying hSYN-driven Cre.

Fig. S6

a

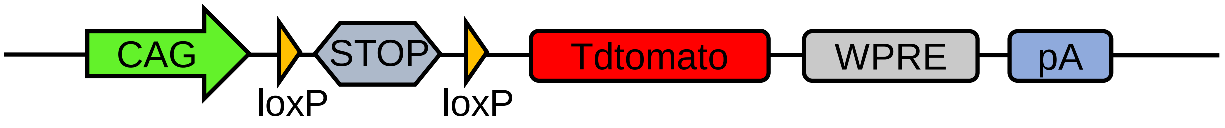
CAG
loxP
STOP
loxP
Tdtomato
WPRE
pA

b

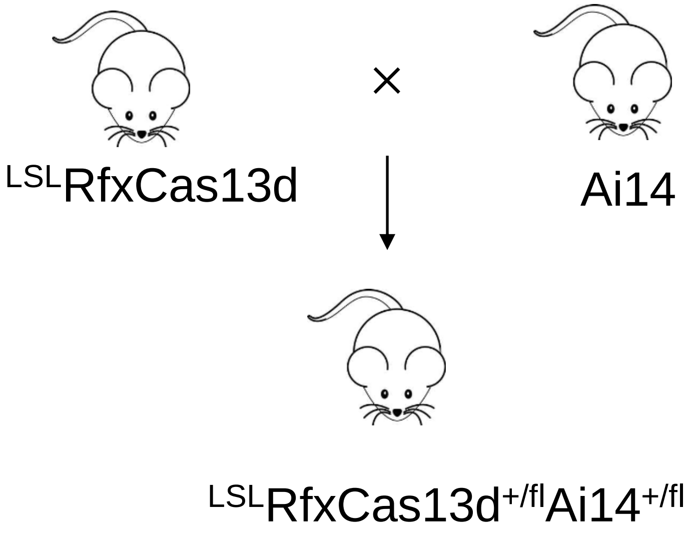
LSLRfxCas13d
×
Ai14
LSLRfxCas13d+/flAi14+/fl

Fig. S6 The generation of ^{LSL}Cas13d$^{+/fl}$Ai14$^{+/fl}$ mice (Linked to Fig. 3). **a.** Schematic diagram of Ai14 reporter mice. **b.** Schematic illustration of generating ^{LSL}RfxCas13d$^{+/fl}$Ai14$^{+/fl}$ mice by crossing ^{LSL}RfxCas13d$^{fl/fl}$ mice with Ai14 mice.

Fig. S7

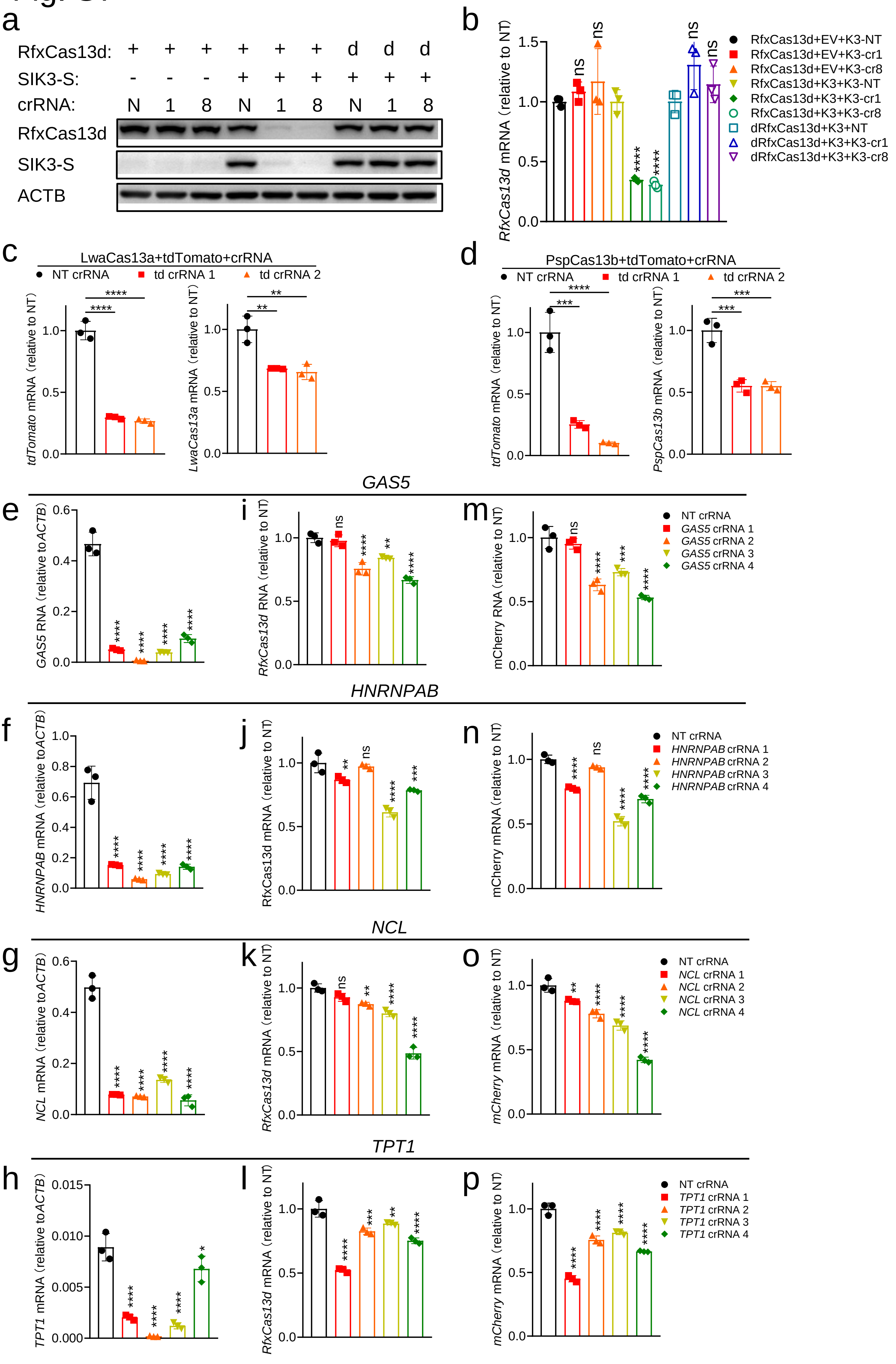
a
RfxCas13d: + + + + + + d d d
SIK3-S: - - - + + + + + +
crRNA: N 1 8 N 1 8 N 1 8
RfxCas13d
SIK3-S
ACTB
b
RfxCas13d mRNA (relative to NT)
RfxCas13d+EV+K3-NT
RfxCas13d+EV+K3-cr1
RfxCas13d+EV+K3-cr8
RfxCas13d+K3+K3-NT
RfxCas13d+K3+K3-cr1
RfxCas13d+K3+K3-cr8
dRfxCas13d+K3+NT
dRfxCas13d+K3+K3-cr1
dRfxCas13d+K3+K3-cr8
c
LwaCas13a+tdTomato+crRNA
NT crRNA
td crRNA 1
td crRNA 2
tdTomato mRNA (relative to NT)
LwaCas13a mRNA (relative to NT)
d
PspCas13b+tdTomato+crRNA
NT crRNA
td crRNA 1
td crRNA 2
tdTomato mRNA (relative to NT)
PspCas13b mRNA (relative to NT)
GAS5
e
GAS5 RNA (relative to ACTB)
i
RfxCas13d RNA (relative to NT)
m
mCherry RNA (relative to NT)
NT crRNA
GAS5 crRNA 1
GAS5 crRNA 2
GAS5 crRNA 3
GAS5 crRNA 4
HNRNPAB
f
HNRNPAB mRNA (relative to ACTB)
j
RfxCas13d mRNA (relative to NT)
n
mCherry mRNA (relative to NT)
NT crRNA
HNRNPAB crRNA 1
HNRNPAB crRNA 2
HNRNPAB crRNA 3
HNRNPAB crRNA 4
NCL
g
NCL mRNA (relative to ACTB)
k
RfxCas13d mRNA (relative to NT)
o
mCherry mRNA (relative to NT)
NT crRNA
NCL crRNA 1
NCL crRNA 2
NCL crRNA 3
NCL crRNA 4
TPT1
h
TPT1 mRNA (relative to ACTB)
l
RfxCas13d mRNA (relative to NT)
p
mCherry mRNA (relative to NT)
NT crRNA
TPT1 crRNA 1
TPT1 crRNA 2
TPT1 crRNA 3
TPT1 crRNA 4

Fig. S7 The collateral activity of RfxCas13d was positively correlated with the abundance of target RNA in mammalian cells (Linked to Fig. 4). **a.** Western blot to measure the expression level of RfxCas13d and SIK3-S 24 h after transfection of plasmids encoding RfxCas13d, SIK3-S and crRNAs into HEK293T cells. **b.** RT-qPCR to measure the RNA level of RfxCas13d in **a**. K3 represents SIK3-S; EV represents empty vector. **c.** RT-qPCR to measure the RNA level of LwaCas13a and tdTomato in HEK293T cells 24 h after transfection of plasmids encoding LwaCas13a, tdTomato and crRNAs (n=3). td crRNA 1/2: tdTomato crRNA 1/2. **d.** RT-qPCR to measure the RNA level of PspCas13b and tdTomato in HEK293T cells 24 h after transfection of plasmids encoding LwaCas13a, tdTomato and crRNAs (n=3). td crRNA 1/2: tdTomato crRNA 1/2. **e-h.** RT-qPCR to measure the knockdown efficiency of GAS5, HNRNPAB, NCL and TPT1 crRNAs in HEK293T cells**. i-p.** RT-qPCR to measure the mRNA levels of RfxCas13d (**i-l**) and mCherry (**m-p**) in the HEK293T cells 24 h after transfection of plasmids respectively encoding RfxCas13d, mCherry and crRNA. **b-p,** One-way ANOVA with Dunn's multiple comparisons test. Significance levels are noted as *P < 0.05, **P < 0.01, ***P < 0.001, ****P < 0.0001 or ns (P > 0.05). All values are presented as mean ± SEM.

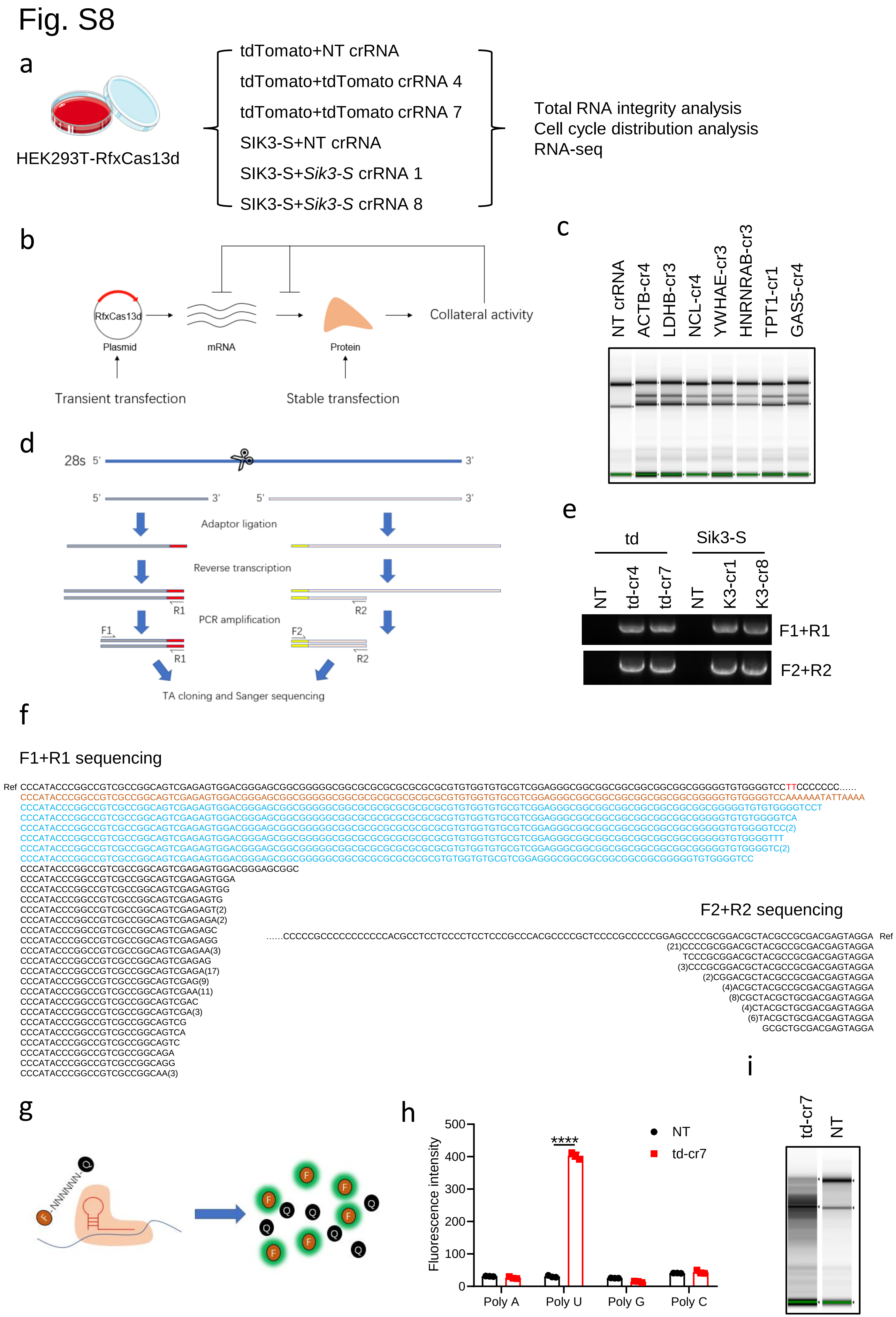
Fig. S8
a
HEK293T-RfxCas13d
tdTomato+NT crRNA
tdTomato+tdTomato crRNA 4
tdTomato+tdTomato crRNA 7
SIK3-S+NT crRNA
SIK3-S+Sik3-S crRNA 1
SIK3-S+Sik3-S crRNA 8
Total RNA integrity analysis
Cell cycle distribution analysis
RNA-seq
b
RfxCas13d
Plasmid
mRNA
Protein
Collateral activity
Transient transfection
Stable transfection
c
NT crRNA
ACTB-cr4
LDHB-cr3
NCL-cr4
YWHAE-cr3
HNRNRAB-cr3
TPT1-cr1
GAS5-cr4
d
28s 5' 3'
5' 3'
5' 3'
Adaptor ligation
Reverse transcription
R1
R2
PCR amplification
F1
F2
R1
R2
TA cloning and Sanger sequencing
e
td
Sik3-S
NT
td-cr4
td-cr7
NT
K3-cr1
K3-cr8
F1+R1
F2+R2
f
F1+R1 sequencing
Ref CCCATACCCGGCCGTCGCCGGCAGTCGAGAGTGGACGGGAGCGGCGGGGGCGGCGCGCGCGCGCGCGTGTGGTGTGCGTCGGAGGGCGGCGGCGGCGGCGGCGGGGGTGTGGGGTCCTTCCCCCCC......
CCCATACCCGGCCGTCGCCGGCAGTCGAGAGTGGACGGGAGCGGCGGGGGCGGCGCGCGCGCGCGCGTGTGGTGTGCGTCGGAGGGCGGCGGCGGCGGCGGCGGGGGTGTGGGGTCCAAAAAATATTAAAA
CCCATACCCGGCCGTCGCCGGCAGTCGAGAGTGGACGGGAGCGGCGGGGGCGGCGCGCGCGCGCGCGTGTGGTGTGCGTCGGAGGGCGGCGGCGGCGGCGGCGGCGGGGGTGTGTGGGGTCCT
CCCATACCCGGCCGTCGCCGGCAGTCGAGAGTGGACGGGAGCGGCGGGGGCGGCGCGCGCGCGCGCGTGTGGTGTGCGTCGGAGGGCGGCGGCGGCGGCGGCGGGGGTGTGTGGGGTCA
CCCATACCCGGCCGTCGCCGGCAGTCGAGAGTGGACGGGAGCGGCGGGGGCGGCGCGCGCGCGCGCGTGTGGTGTGCGTCGGAGGGCGGCGGCGGCGGCGGCGGGGGTGTGGGGTCC(2)
CCCATACCCGGCCGTCGCCGGCAGTCGAGAGTGGACGGGAGCGGCGGGGGCGGCGCGCGCGCGCGCGTGTGGTGTGCGTCGGAGGGCGGCGGCGGCGGCGGCGGGGGTGTGGGGTTT
CCCATACCCGGCCGTCGCCGGCAGTCGAGAGTGGACGGGAGCGGCGGGGGCGGCGCGCGCGCGCGCGTGTGGTGTGCGTCGGAGGGCGGCGGCGGCGGCGGCGGGGGTGTGGGGTC(2)
CCCATACCCGGCCGTCGCCGGCAGTCGAGAGTGGACGGGAGCGGCGGGGGCGGCGCGCGCGCGCGTGTGGTGTGCGTCGGAGGGCGGCGGCGGCGGCGGGGGTGTGGGGTCC
CCCATACCCGGCCGTCGCCGGCAGTCGAGAGTGGACGGGAGCGGC
CCCATACCCGGCCGTCGCCGGCAGTCGAGAGTGGA
CCCATACCCGGCCGTCGCCGGCAGTCGAGAGTGG
CCCATACCCGGCCGTCGCCGGCAGTCGAGAGTG
CCCATACCCGGCCGTCGCCGGCAGTCGAGAGT(2)
CCCATACCCGGCCGTCGCCGGCAGTCGAGAGA(2)
CCCATACCCGGCCGTCGCCGGCAGTCGAGAGC
CCCATACCCGGCCGTCGCCGGCAGTCGAGAGG
CCCATACCCGGCCGTCGCCGGCAGTCGAGAA(3)
CCCATACCCGGCCGTCGCCGGCAGTCGAGAG
CCCATACCCGGCCGTCGCCGGCAGTCGAGA(17)
CCCATACCCGGCCGTCGCCGGCAGTCGAG(9)
CCCATACCCGGCCGTCGCCGGCAGTCGAA(11)
CCCATACCCGGCCGTCGCCGGCAGTCGAC
CCCATACCCGGCCGTCGCCGGCAGTCGA(3)
CCCATACCCGGCCGTCGCCGGCAGTCG
CCCATACCCGGCCGTCGCCGGCAGTCA
CCCATACCCGGCCGTCGCCGGCAGTC
CCCATACCCGGCCGTCGCCGGCAGA
CCCATACCCGGCCGTCGCCGGCAGG
CCCATACCCGGCCGTCGCCGGCAA(3)
F2+R2 sequencing
......CCCCCGCCCCCCCCCCCACGCCTCCTCCCCTCCTCCCGCCCACGCCCCGCTCCCCGCCCCCGGAGCCCCGCGGACGCTACGCCGCGACGAGTAGGA Ref
(21)CCCCGCGGACGCTACGCCGCGACGAGTAGGA
TCCCGCGGACGCTACGCCGCGACGAGTAGGA
(3)CCCGCGGACGCTACGCCGCGACGAGTAGGA
(2)CGGACGCTACGCCGCGACGAGTAGGA
(4)ACGCTACGCCGCGACGAGTAGGA
(8)CGCTACGCTGCGACGAGTAGGA
(4)CTACGCTGCGACGAGTAGGA
(6)TACGCTGCGACGAGTAGGA
GCGCTGCGACGAGTAGGA
g
h
500
400
300
200
100
0
Fluorescence intensity
****
NT
td-cr7
Poly A
Poly U
Poly G
Poly C
i
td-cr7
NT

Fig. S8 The collateral activity of RfxCas13d caused 28s rRNA breakage (Linked to Fig 5). **a.** Workflow of experiments did in HEK293T-RfxCas13d cells. **b.** Schematic illustration that the collateral activity of RfxCas13d cleaves its own mRNA and 28s RNA, thereby inhibiting its own protein expression. **c.** Quality control of total RNA of WT HEK293T cells transfected with plasmids encoding RfxCas13d and indicated crRNAsby Agilent 2200 Bioanalyzer. cr: crRNA. **d.** Schematic illustration of oligonucleotide extension essay. Red represents oligonucleotide adaptor 1; Yellow represents oligonucleotide adaptor 2. **e.** Gel picture of PCR products from d. **f.** Sanger sequencing results. Ref: reference sequence. The numbers in brackets represent the number of identical sequencing results. **g.** Schematic illustration of reconstituting the collateral activity of RfxCas13d *in vitro*. **h.** Statistical diagram of fluorescence intensity in **g**. **i.** Quality control of total RNA by Agilent 2200 Bioanalyzer. **h,** Two-tail unpaired t test. Significance levels are noted as ***P < 0.001 or ns (P > 0.05). All values are presented as mean ± SEM.

Fig. S9

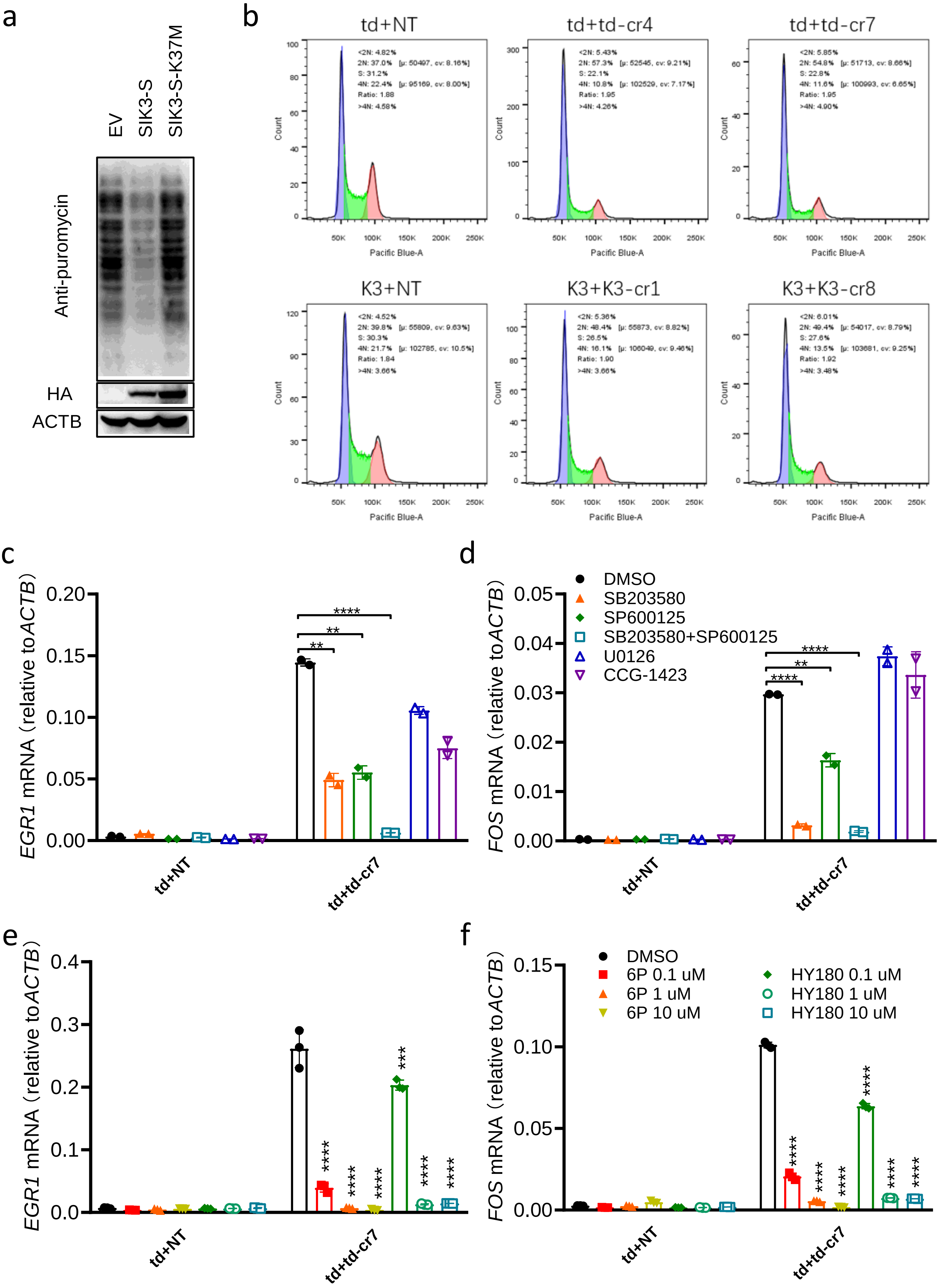

a
EV
SIK3-S
SIK3-S-K37M
Anti-puromycin
HA
ACTB
b
td+NT
<2N: 4.82%
2N: 37.0% [μ: 50497, cv: 8.16%]
S: 31.2%
4N: 22.4% [μ: 95169, cv: 8.00%]
Ratio: 1.88
>4N: 4.58%
td+td-cr4
<2N: 5.43%
2N: 57.3% [μ: 52545, cv: 9.21%]
S: 22.1%
4N: 10.8% [μ: 102529, cv: 7.17%]
Ratio: 1.95
>4N: 4.26%
td+td-cr7
<2N: 5.85%
2N: 54.8% [μ: 51713, cv: 8.66%]
S: 22.8%
4N: 11.6% [μ: 100993, cv: 6.65%]
Ratio: 1.95
>4N: 4.90%
K3+NT
<2N: 4.52%
2N: 39.8% [μ: 55809, cv: 9.63%]
S: 30.3%
4N: 21.7% [μ: 102785, cv: 10.5%]
Ratio: 1.84
>4N: 3.66%
K3+K3-cr1
<2N: 5.36%
2N: 48.4% [μ: 55873, cv: 8.82%]
S: 26.5%
4N: 16.1% [μ: 106049, cv: 9.46%]
Ratio: 1.90
>4N: 3.66%
K3+K3-cr8
<2N: 6.01%
2N: 49.4% [μ: 54017, cv: 8.79%]
S: 27.6%
4N: 13.5% [μ: 103681, cv: 9.25%]
Ratio: 1.92
>4N: 3.48%
Count
Pacific Blue-A
c
EGR1 mRNA (relative to ACTB)
td+NT
td+td-cr7
d
FOS mRNA (relative to ACTB)
DMSO
SB203580
SP600125
SB203580+SP600125
U0126
CCG-1423
td+NT
td+td-cr7
e
EGR1 mRNA (relative to ACTB)
td+NT
td+td-cr7
f
FOS mRNA (relative to ACTB)
DMSO
6P 0.1 uM
6P 1 uM
6P 10 uM
HY180 0.1 uM
HY180 1 uM
HY180 10 uM
td+NT
td+td-cr7

Fig S9. The collateral activity of RfxCas13d caused translation attenuation, cell cycle arrest and activation of ZAKα-JNK/p38-IEG pathway (Linked to Fig. 5). **a.** SUnSET essay to measure the protein translation rate of HEK293T-RfxCas13d cells 24 h after transfection of plasmids encoding SIK3-S or SIK3-S-K37M. **b.** Cell cycle analysis using FCAS. **c-d.** RT-qPCR to measure the RNA level of EGR1 and FOS in HEK293T-RfxCas13d cells 24 h after transfection of plasmids encoding tdTomato and crRNAs. Inhibitors and transfection mix were added together. SB203580: p38 inhibitor; SP600125: JNK inhibitor; U0126: MEK1/2 inhibitor; CCG-1423: RhoA/C inhibitor. **e-f.** RT-qPCR to measure the RNA level of EGR1 and FOS in HEK293T-RfxCas13d cells 24 h after transfection of plasmids encoding tdTomato and crRNAs into. ZAK inhibitors and transfection mix were added together. 6p and HY180: ZAK inhibitors. **c-d,** Two-tail unpaired t test. **e-f**, One-way ANOVA with Dunn's multiple comparisons test. Significance levels are noted as **P < 0.01, ***P < 0.001, ****P < 0.0001 or ns (P > 0.05). All values are presented as mean ± SEM.

Fig. S10

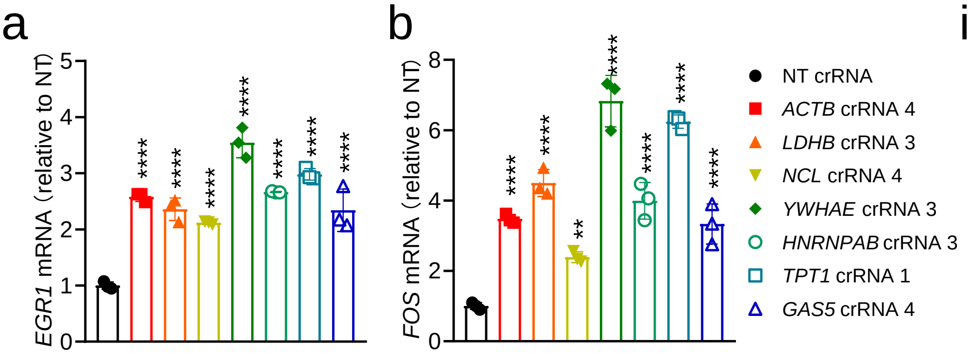

i

Gene	FPKM
ACTB	1160.6
YWHAE	502.5
LDHB	477.3
NCL	222.6
HNRNPAB	209.8
TPT1	193.0
GAS5	137.0
ANXA4	2.8
B4GALNT1	1.7

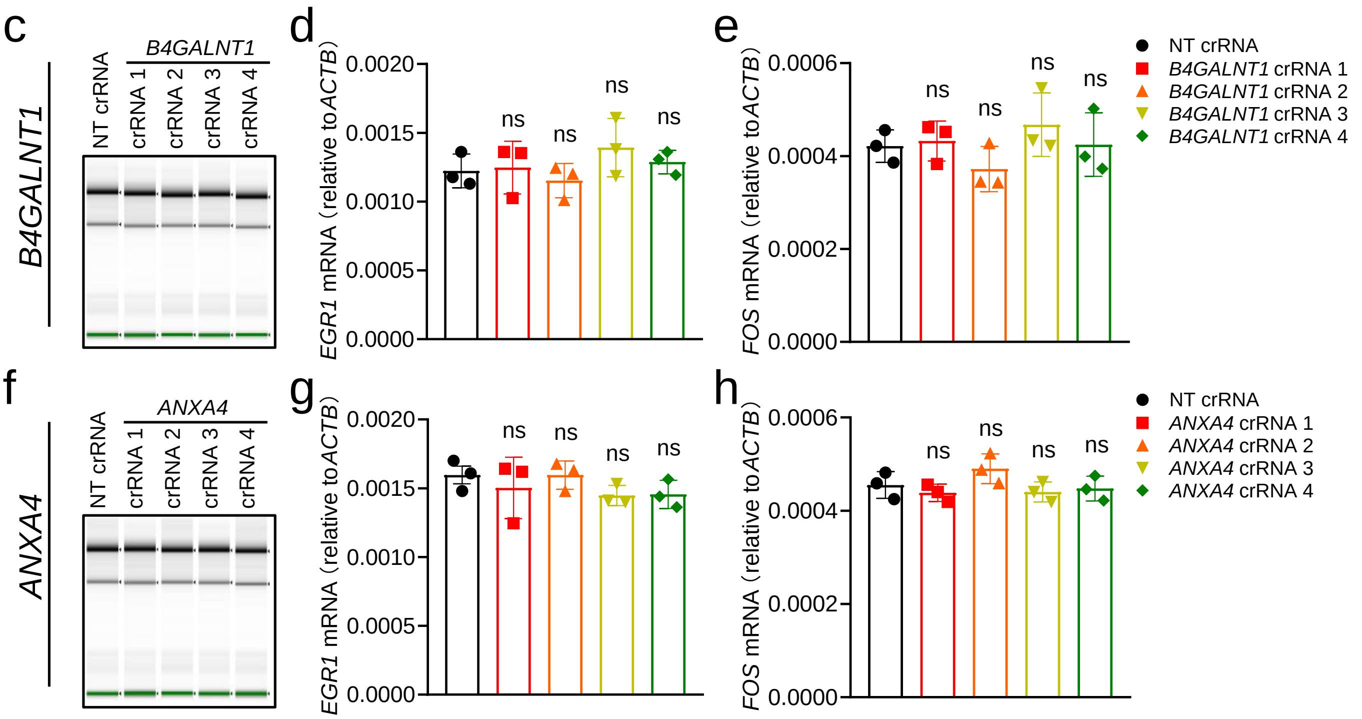

Fig. S10 RfxCas13d exhibited collateral activity upon targeting highly-expressed endogenous genes (Linked to Fig. 5). **a-b.** RT-qPCR to measure the RNA level of EGR1 (**a**) and FOS (**b**) in WT HEK293T cells 24 h after transfection of plasmids encoding RfxCas13d and NT crRNA (n=3) or indicated crRNAs (n=3). **c-e.** WT HEK293T cells were collected for total RNA integrity analysis and RT-qPCR 24 h after transfection of plasmids encoding RfxCas13d and NT crRNA (n=3) or B4GALNT1 crRNAs (n=3). **c.** Quality control of total RNA of each sample was quantified by Agilent 2200 Bioanalyzer. **d-e.** RT-qPCR to measure the RNA level of EGR1 (**a**) and FOS (**b**) in each sample. **f-h.** WT HEK293T cells were collected for total RNA integrity analysis and RT-qPCR 24 h after transfection of plasmids encoding RfxCas13d and NT crRNA (n=3) or ANXA4 crRNAs (n=3). **f.** Quality control of total RNA of each sample was quantified by Agilent 2200 Bioanalyzer. **g-h.** RT-qPCR to measure the RNA level of EGR1 (**a**) and FOS (**b**) in each sample. **i.** The table to show the expression levels (FPKM) of indicated genes in HEK293T cells (data from RNA-seq). **a-b, d-e, g-h,** One-way ANOVA with Dunn's multiple comparisons test. Significance levels are noted as $^{**}P < 0.01$, $^{****}P < 0.0001$ or ns ($P > 0.05$). All values are presented as mean ± SEM.

Fig. S11

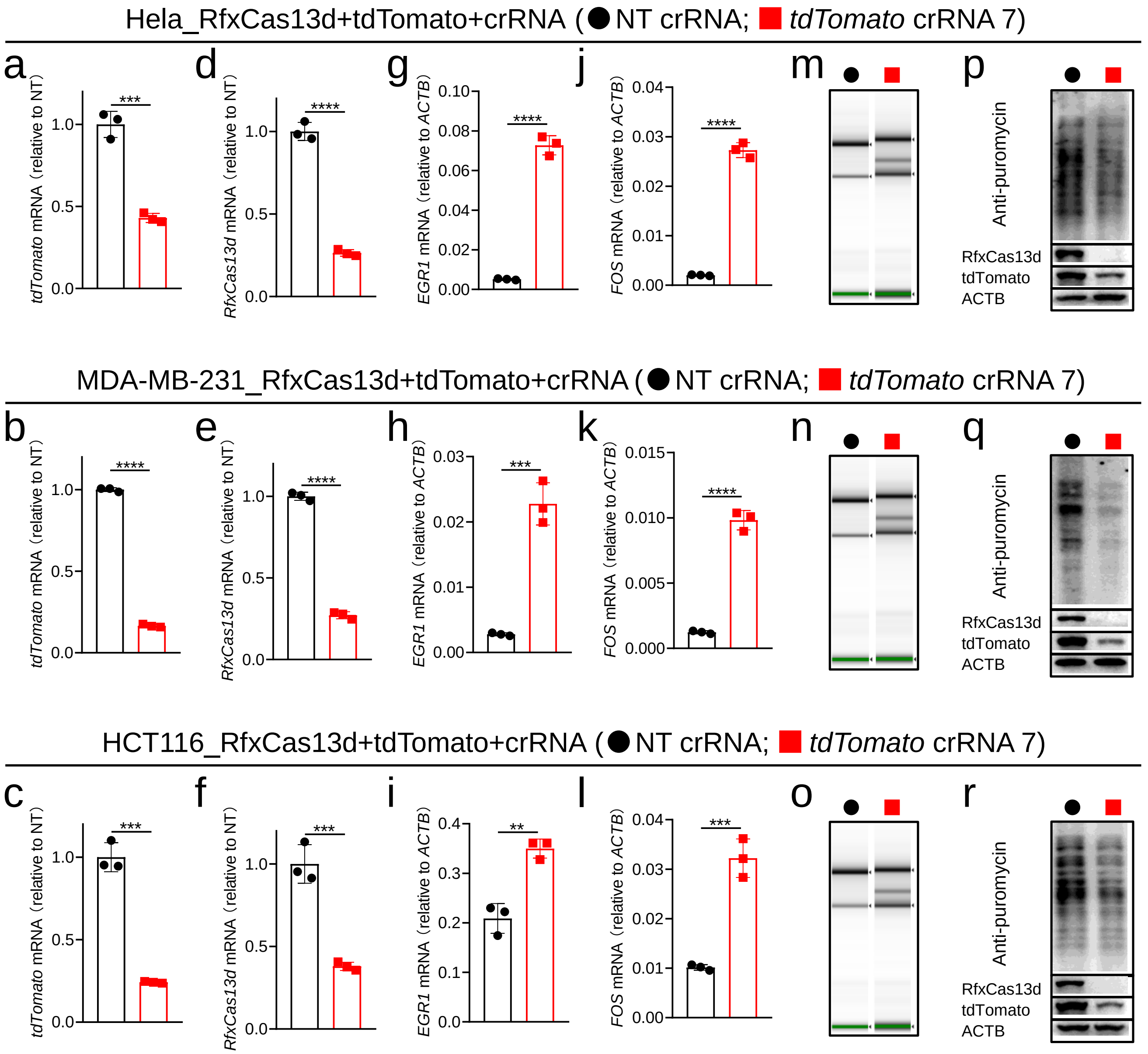

Hela_RfxCas13d+tdTomato+crRNA (● NT crRNA; ■ tdTomato crRNA 7)
MDA-MB-231_RfxCas13d+tdTomato+crRNA (● NT crRNA; ■ tdTomato crRNA 7)
HCT116_RfxCas13d+tdTomato+crRNA (● NT crRNA; ■ tdTomato crRNA 7)
a
b
c
d
e
f
g
h
i
j
k
l
m
n
o
p
q
r
tdTomato mRNA (relative to NT)
RfxCas13d mRNA (relative to NT)
EGR1 mRNA (relative to ACTB)
FOS mRNA (relative to ACTB)
Anti-puromycin
RfxCas13d
tdTomato
ACTB

Fig. S11 RfxCas13d exhibited collateral activity in Hela, MDA-MB-231 and HCT116 cells (Linked to Fig. 5). RT-qPCR, total RNA integrity analysis and SUnSET were used to measure the expression levels of indicated genes, RNA integrity and protein synthesis respectively in Hela, MDA-MB-231and HCT116 cells 24 h after transfection of plasmids encoding RfxCas13d, tdTomato and NT crRNA or tdTomato crRNA 7. **a-l.** RT-qPCR to measure the mRNA levels of tdTomato (**a-c**), RfxCas13d (**d-f**), EGR1 (**g-i**) and FOS (**j-l**). **m-o.** Quality control of total RNA of samples by Agilent 2200 Bioanalyzer. **p-r.** SUnSET essay to measure the protein translation rate. **a-l,** two-tailed unpaired t-test (n=3). Significance levels are noted as $**P < 0.01$, $***P < 0.001$, $****P < 0.0001$ or ns ($P > 0.05$). All values are presented as mean ± SEM.

Fig. S12

a

Gene	N2a	Brain
ACTB	1296.69	573.68
Sik3	8.84	21.39
Map2	6.16	80.91
Mapt	22.76	101.10
Rbfox3	0.03	57.27

b

Group	Comparison	DEGs
1	dRfxCas13d vs. RfxCas13d	6
2	dRfxCas13d+NT crRNA vs. RfxCas13d+NT crRNA	194
3	dRfxCas13d+*Sik3-S* crRNA 1 vs. RfxCas13d+*Sik3-S* crRNA 1	898
4	dRfxCas13d+*Sik3-S* crRNA 8 vs. RfxCas13d+*Sik3-S* crRNA 8	909
5	dRfxCas13d+*Map2* crRNA 1 vs. RfxCas13d+*Map2* crRNA 1	1179
6	dRfxCas13d+*Map2* crRNA 3 vs. RfxCas13d+*Map2* crRNA 3	447
7	dRfxCas13d+*Mapt* crRNA 4 vs. RfxCas13d+*Mapt* crRNA 4	772
8	dRfxCas13d+*Mapt* crRNA 6 vs. RfxCas13d+*Mapt* crRNA 6	715
9	dRfxCas13d+*Rbfox3* crRNA 5 vs. RfxCas13d+*Rbfox3* crRNA 5	760

c

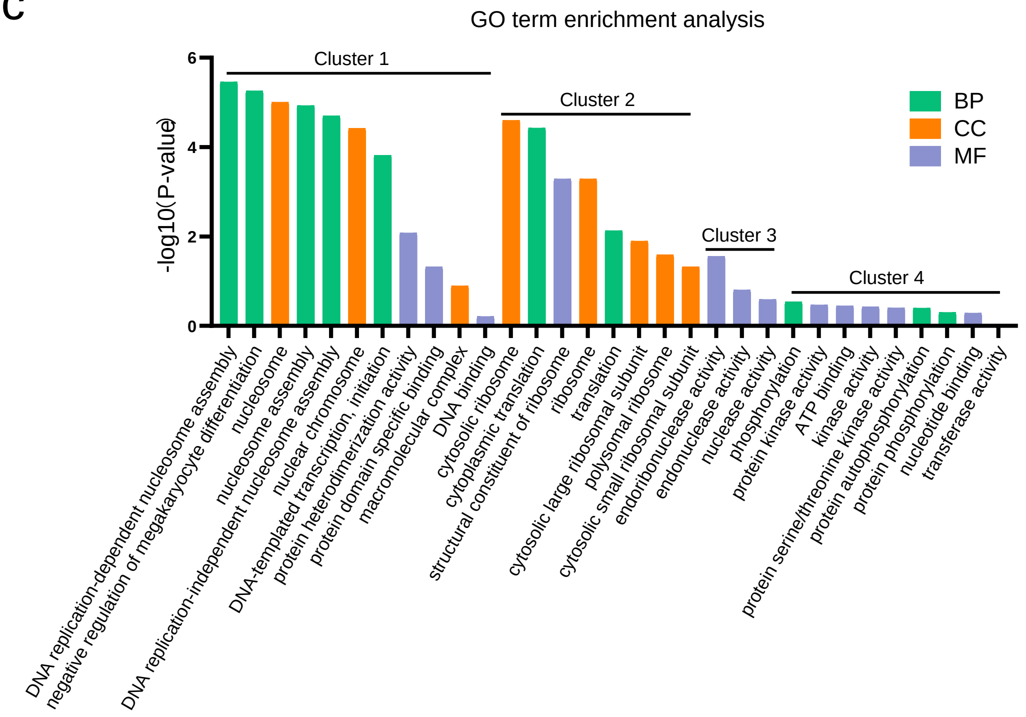

d

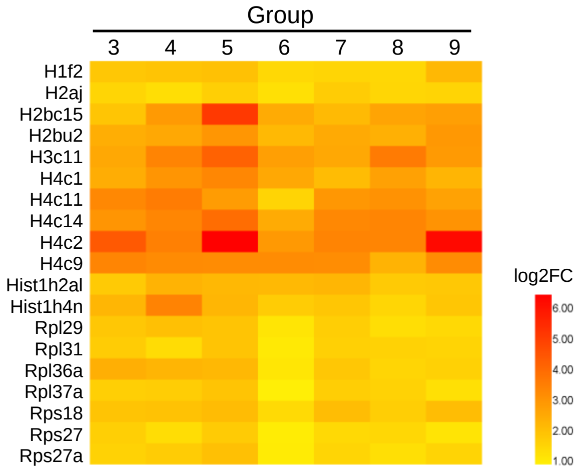

e

Group	Comparison	DEGs
10	dRfxCas13d+NT crRNA vs. dRfxCas13d+*Sik3-S* crRNA 1	0
11	dRfxCas13d+NT crRNA vs. dRfxCas13d+*Sik3-S* crRNA 8	0
12	dRfxCas13d+NT crRNA vs. dRfxCas13d+*Map2* crRNA 1	0
13	dRfxCas13d+NT crRNA vs. dRfxCas13d+*Map2* crRNA 3	0
14	dRfxCas13d+NT crRNA vs. dRfxCas13d+*Mapt* crRNA 4	0
15	dRfxCas13d+NT crRNA vs. dRfxCas13d+*Mapt* crRNA 6	17
16	dRfxCas13d+NT crRNA vs. dRfxCas13d+*Rbfox3* crRNA 5	3

Fig. S12 RfxCas13d exhibits collateral activity in N2a cells when targeting *Sik3-S*, *Map2*, *Mapt* and *Rbfox3*. **a**. The table to show the expression levels (FPKM) of indicated genes in N2a cells and mouse brain (data from RNA-seq analysis of N2a cells and bulk RNA-seq analysis of brains). **b.** The table to show the RNA-seq samples we did in N2a cells, the group comparisons made, and the number of identified DEGs (P adj < 0.05 and change fold ≥2). n=2. **c.** GO term enrichment analysis (David online) of 185common DEGs from seven sets of comparisons (Group 3-9). BP, CC and MF represents biological process, cellular component and molecular function respectively. **d.** Heatmap showing the log2 fold-change (log2FC) values of nucleosome and ribosome-related DEGs from seven sets of comparisons in N2a cells (Group 3-9). **e.** The table to show the group comparisons made, and the number of DEGs.
