# Supplementary material for: The collateral activity of RfxCas13d can induce lethality in a RfxCas13d knock-in mouse model
Source: Genome Biol. 2023 Feb 1;24:20. doi: 10.1186/s13059-023-02860-w (PMC9893547; doi:10.1186/s13059-023-02860-w)
Supplement: Supplementary file 6 — Additional file 6: Table S5. The sequence of the primers used in this study. [file 13059_2023_2860_MOESM6_ESM.docx]

The sequence of qPCR primers used in this study.

| Gene Name | Species | Forward primer | Reverse primer |
| --- | --- | --- | --- |
| Sik3-S | Mouse | CAGAGCGACAGTGACCATCA | GCTGGATTCTGGTGAAAACCC |
| Map2 | Mouse | ACCTTCCTCCATCCTCCCTC | TCCTGCTCTGCGAATTGGTT |
| Mapt | Mouse | CCTCCTAGGCCTGACTCCTT | CTGGGGTGGAAGCGAAAGAT |
| Actb | Mouse | TTACGGATGTCAACGTCACAGTTC | ACTATTGGCAACGAGCGGTTC |
| TPT1 | Human | GGACTACCGTGAGGATGGTG | GGATTTCTTTCTTTTGCATCACATT |
| LDHB | Human | GCCTTCTCTCTCCTGTGCAA | CCTCTTCTTCCGCAACTGGT |
| NCL | Human | AAGCGTTGGAACTCACTGGT | TCTCGCATCTCGCTCTTTCT |
| YWHAE | Human | GCAGAACTGGATACGCTGAG | ATTCTGCTCTTCACCGTCAC |
| HNRNPAB | Human | TGTCAGTGGAAGCAAGTGTGA | CTGGTTCCAGTAGTTGCCGT |
| GAS5 | Human | TATGGTGCTGGGTGCAGATG | CCATTAAGCTGGTCCAGGCA |
| EGR1 | Human | ACCTGACCGCAGAGTCTTTT | CAAGGTGTTGCCACTGTTGG |
| FOS | Human | TACACTCCAAGCGGAGACAG | TCCTTCAGCAGGTTGGCAAT |
| GAPDH | Human | CAGCCTCAAGATCATCAGCA | TGTGGTCATGAGTCCTTCCA |
| ACTB | Human | AAAGACCTGTACGCCAACAC | GTCATACTCCTGCTTGCTGAT |
| RfxCas13d | For cells | GACCCGTCCAGCAGGATATG | AGGCGGCGTTGGTAATGTAT |
| RfxCas13d | For mice | ACTCCCTGGAGTCCGCTAAT | GTTAAGAGCGGTCTTGGCCT |
| tdTomato |  | CCTGTTCCTGGGGCATGGCA | TCTTTGATGACGGCCATGTTGTTGT |
| EGFP |  | CGTAAACGGCCACAAGTTCA | CTTCATGTGGTCGGGGTAGC |

The sequence of the primers used for genotyping in this study.

| Primer | Sequence |
| --- | --- |
| Gt-F1 | GTCATTGTACACATCTGTAAAAGGTGGTTCCTTTTGGAATGCAAAGT |
| Gt-R1 | AGCTTCACAAATACATTTGTTGCTTAATCATTTTACTTGTGTTTGGCT |
| Gt-F2 | ACCTTGGCGTCTTCCTCGATGTAGTATCTGTAGATCACG |
| Gt-R2 | ACCTTGGCGTCTTCCTCGATGTAGTATCTGTAGATCACG |

The sequence of the primers used for identifying the other possible RfxCas13d variants in this study.

| Primer | Sequence |
| --- | --- |
| F1 | GAAGAGCTTCGCTAAGGGCA |
| R1 | TGGGTACGATCCGACCTTCC |
| F2 | AAGAAGGACGCCCCTAGACT |
| R2 | CACAGCTTGGTCACGCTAGA |
| F3 | TCACTAACGCTGCCTATGCC |
| R3 | AGTCTAGGGGCGTCCTTCTT |
| F4 | GCAGTGGTGAAGTTCGTCCT |
| R4 | GCCTCGATGCTCAGGTTCTT |
