# Supplementary material for: The collateral activity of RfxCas13d can induce lethality in a RfxCas13d knock-in mouse model
Source: Genome Biol. 2023 Feb 1;24:20. doi: 10.1186/s13059-023-02860-w (PMC9893547; doi:10.1186/s13059-023-02860-w)
Supplement: Supplementary file 7 — Additional file 7: Table S6. The sequence of the crRNAs and gRNAs used in this study. [file 13059_2023_2860_MOESM7_ESM.docx]

The sequence of the crRNAs and gRNAs used in this study.

| Subtypes of Cas13 | crRNA name | Sequence |
| --- | --- | --- |
| RfxCas13d | NT crRNA | TCACCAGAAGCGTACCATACTC |
| RfxCas13d | Rbfox3 crRNA 1 | CTGCATAGAATTCAGGCCCATA |
| RfxCas13d | Rbfox3 crRNA 2 | GAAATGTATTATACACAGCACG |
| RfxCas13d | Rbfox3 crRNA 3 | GCCTCCATAAATCTCAGCACCA |
| RfxCas13d | Rbfox3 crRNA 4 | GAGCATATCTGTAAGCTGCATA |
| RfxCas13d | Rbfox3 crRNA 5 | AAATCCATCCTGATACACGACC |
| RfxCas13d | Rbfox3 crRNA 6 | GCAGCAACATTCAATGAGGCCA |
| RfxCas13d | Sik3-S crRNA 1 | CAAGCACTGCCAGGTGCCACAT |
| RfxCas13d | Sik3-S crRNA 2 | GCAGGGGAGCCTGCCCAAGGAC |
| RfxCas13d | Sik3-S crRNA 3 | AATGGCGCTTATAGAAATGAAA |
| RfxCas13d | Sik3-S crRNA 4 | GAAGAGGGAGGGAGGAGAGAAA |
| RfxCas13d | Sik3-S crRNA 5 | TCACAGAAAATAAGAAAGACAA |
| RfxCas13d | Sik3-S crRNA 6 | TCTCCAATCTCCAACTCCTTTT |
| RfxCas13d | Sik3-S crRNA 7 | GGAGATATTCATTCATTCATTC |
| RfxCas13d | Sik3-S crRNA 8 | GTCGCTCTGTGAATCAGGCATC |
| RfxCas13d | Map2 crRNA 1 | ACTCTCAATTTTCACACGTCCA |
| RfxCas13d | Map2 crRNA 2 | TCAATCTTCACATTACCACCTC |
| RfxCas13d | Map2 crRNA 3 | GAGCATTGTCAAGTGAGCCAAC |
| RfxCas13d | Map2 crRNA 4 | CTTCGCCTGTTTAAAAGCACCA |
| RfxCas13d | Map2 crRNA 5 | CCATGCAAAACAGAGCAGAGCG |
| RfxCas13d | Map2 crRNA 6 | AAAGGAGAAGTATTCACAAGCC |
| RfxCas13d | Mapt crRNA 1 | GTGATATTATCCAAGGAGCCAA |
| RfxCas13d | Mapt crRNA 2 | CCACATCCCAGAATACCACCCC |
| RfxCas13d | Mapt crRNA 3 | GCCAAGCATGAGAACAGGCAGA |
| RfxCas13d | Mapt crRNA 4 | CTTAGATAAAAGAAAAGGCAGA |
| RfxCas13d | Mapt crRNA 5 | AACTACAACGTAACAGGGCGAA |
| RfxCas13d | Mapt crRNA 6 | ATAAAAGAAAAGGCAGAGGTCC |
| RfxCas13d | tdTomato crRNA 1 | CCCTCGGAGCGCTCGTACTGTT |
| RfxCas13d | tdTomato crRNA 2 | GGTGCCCTCGTAGGGGCGGCCC |
| RfxCas13d | tdTomato crRNA 3 | GGGGGACAGGATGTCCCAGGCG |
| RfxCas13d | tdTomato crRNA 4 | AGGAGTCCTGGGTCACGGTCAC |
| RfxCas13d | tdTomato crRNA 5 | TCTTGGCCATGTAGATGGTCTT |
| RfxCas13d | tdTomato crRNA 6 | CCAGACCGCCGTCCTCGAAGTT |
| RfxCas13d | tdTomato crRNA 7 | GTCACCTTCAGCTTGGCGGTCT |
| RfxCas13d | GAS5 crRNA 1 | CACACAGTGTAGTCAAGCCGAC |
| RfxCas13d | GAS5 crRNA 2 | ATAAAAACGTTACCAGGAGCAG |
| RfxCas13d | GAS5 crRNA 3 | AATTTATTAAAATTGGAGACAC |
| RfxCas13d | GAS5 crRNA 4 | ATAAAAACGTTACCAGGAGCAG |
| RfxCas13d | HNRNPAB crRNA 1 | TTATTGTACAGTCAACGACCTC |
| RfxCas13d | HNRNPAB crRNA 2 | CAACTCTGACTCTGACCTCCAC |
| RfxCas13d | HNRNPAB crRNA 3 | CAAACAAAGCATGTGTGCGATC |
| RfxCas13d | HNRNPAB crRNA 4 | CCTGGTAATAAAAATCAGCCCA |
| RfxCas13d | TPT1 crRNA 1 | GTTCATGACAATATCGACACCA |
| RfxCas13d | TPT1 crRNA 2 | AGTCCAATAGAGCAACCATGCC |
| RfxCas13d | TPT1 crRNA 3 | ATTACCATTAACATGCAGCCTA |
| RfxCas13d | TPT1 crRNA 4 | AGCTCAAGATGACATCAGTCCC |
| RfxCas13d | LDHB crRNA 1 | CCACAAGAGCAAGTTCATCAGC |
| RfxCas13d | LDHB crRNA 2 | CCCAGAATGCTGATAGCACACG |
| RfxCas13d | LDHB crRNA 3 | GGATTCAATAAGATCAGCCACA |
| RfxCas13d | LDHB crRNA 4 | CACACTTAATCCAATAGCCCAG |
| RfxCas13d | NCL crRNA 1 | CCAACAAAGAGATTGAAAGCCG |
| RfxCas13d | NCL crRNA 2 | TCCAACAAAGAGATTGAAAGCC |
| RfxCas13d | NCL crRNA 3 | CAGGTAACAGTAAAAACCCCAG |
| RfxCas13d | NCL crRNA 4 | GCAAACTCTATAAATGCATACC |
| RfxCas13d | YWHAE crRNA 1 | CGGAAAAATTGAGAGCAAGACC |
| RfxCas13d | YWHAE crRNA 2 | GTCATTGCAATATCACTAGCAG |
| RfxCas13d | YWHAE crRNA 3 | AATAGAAAACCTTGGACTCGCC |
| RfxCas13d | YWHAE crRNA 4 | CTACTTTCTTCATTGACTCCAC |
| RfxCas13d | ACTB crRNA 1 | TGAAGCTGTAGCCGCGCTCGGT |
| RfxCas13d | ACTB crRNA 2 | TGAACTTTGGGGGATGCTCGCT |
| RfxCas13d | ACTB crRNA 3 | CGTACAGGGATAGCACAGCCTG |
| RfxCas13d | ACTB crRNA 4 | ATCTTGATCTTCATTGTGCTGG |
|  |  |  |
| LwaCas13a | NT crRNA | CAGACTATGCGTCGACAAGCCAGGCATT |
| LwaCas13a | tdTomato crRNA 1 | AAGCGCATGAACTCTTTGATGACCTCCT |
| LwaCas13a | tdTomato crRNA 2 | TAGATGGTCTTGAACTCCACCAGGTAGT |
|  |  |  |
| PspCas13b | NT crRNA | GTAATGCCTGGCTTGTCGACGCATAGTC |
| PspCas13b | tdTomato crRNA 1 | AGGAGTCCTGGGTCACGGTCACCAGACC |
| PspCas13b | tdTomato crRNA 2 | GTCACCTTCAGCTTGGCGGTCTGGGTGC |
|  |  |  |
| spCas9 | PKR gRNA | TTATCCATGGGGAATTACAT |
| spCas9 | ZAK gRNA | ATTACCTTTGGCTACATCAG |
