# Supplementary material for: The collateral activity of RfxCas13d can induce lethality in a RfxCas13d knock-in mouse model
Source: Genome Biol. 2023 Feb 1;24:20. doi: 10.1186/s13059-023-02860-w (PMC9893547; doi:10.1186/s13059-023-02860-w)
Supplement: Supplementary file 8 — Additional file 8. Uncropped western blot images. [file 13059_2023_2860_MOESM8_ESM.pdf]

Fig. S1

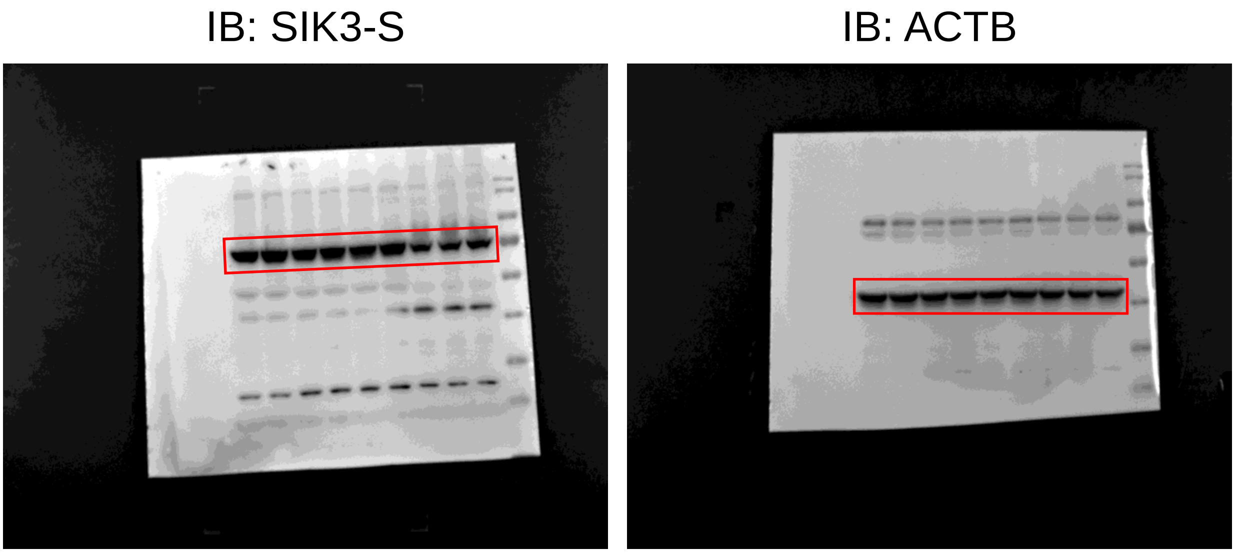

Fig. S1 Uncropped western blot images for Fig. 1g . Red boxes indicate cropped regions.

Fig. S2

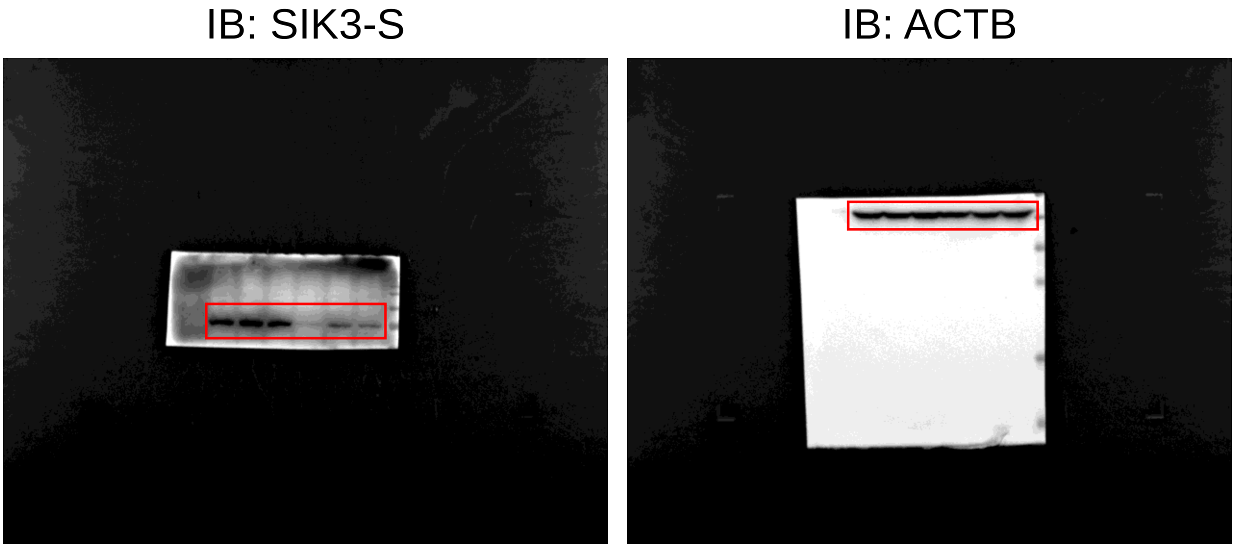

Fig. S2 Uncropped western blot images for Fig. 2a . Red boxes indicate cropped regions.

Fig. S3

IB: HA (RfxCas13d)

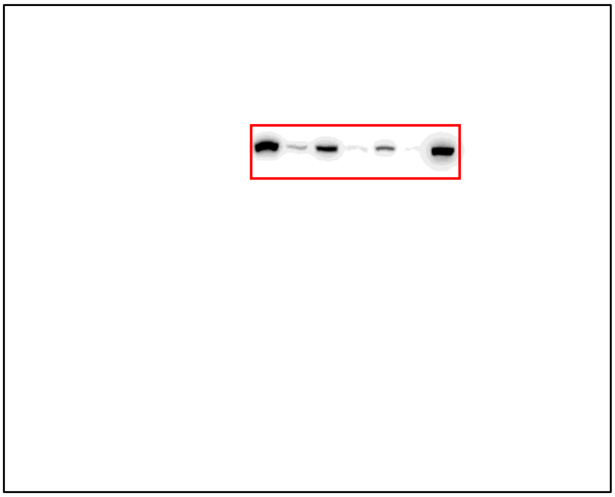

IB: HA (NeuN)

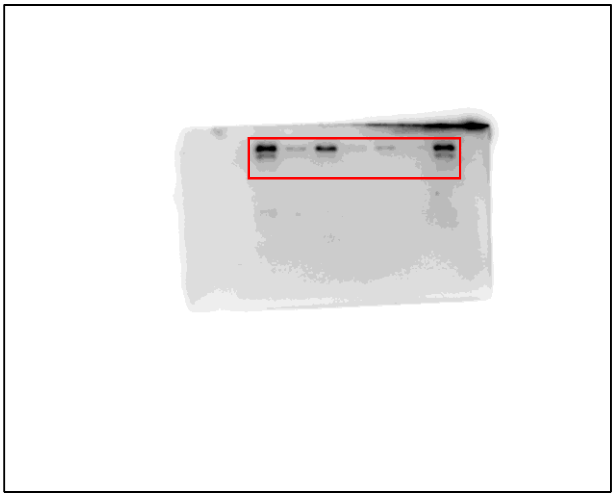

IB: ACTB

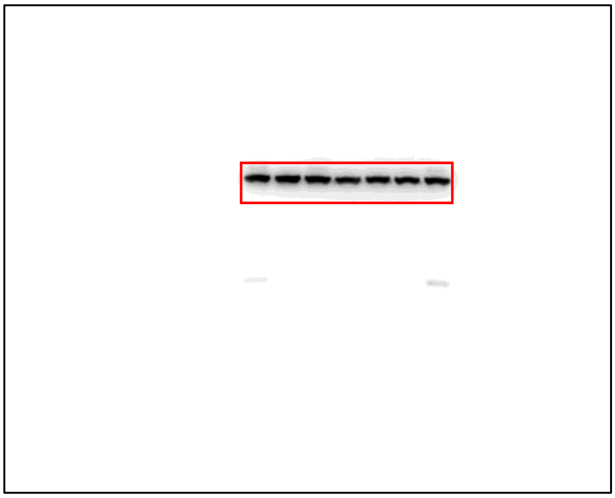

Fig. S3 Uncropped western blot images for Fig. 2f/Response Fig. 8c (First revision)/ Response Fig. 3a (Second revision). Red boxes indicate cropped regions.

Fig. S4

IB: MAP2 IB: ACTB

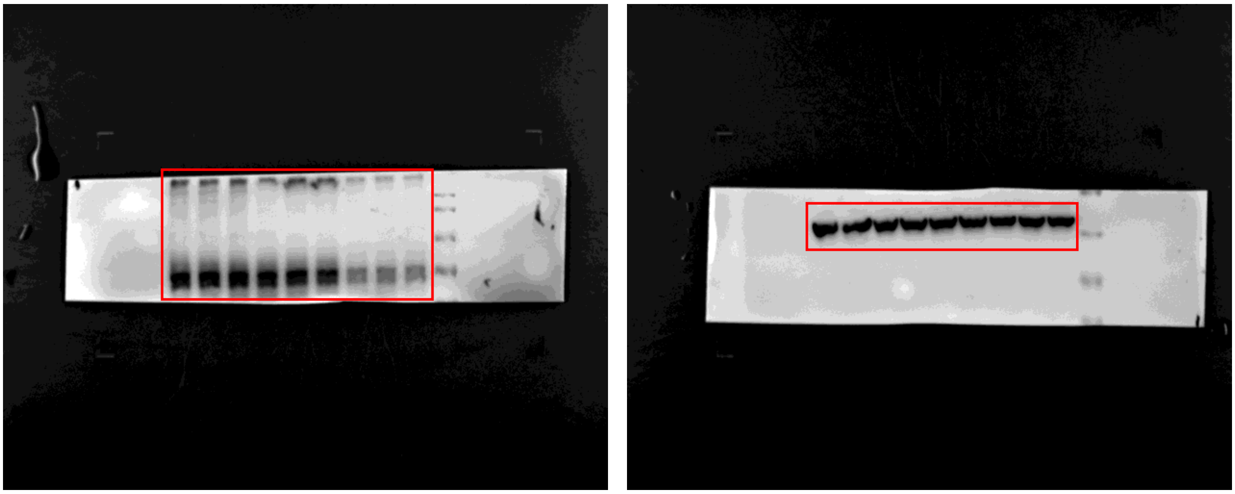

Fig. S4 Uncropped western blot images for Fig. 2p. Red boxes indicate cropped regions.

Fig. S5

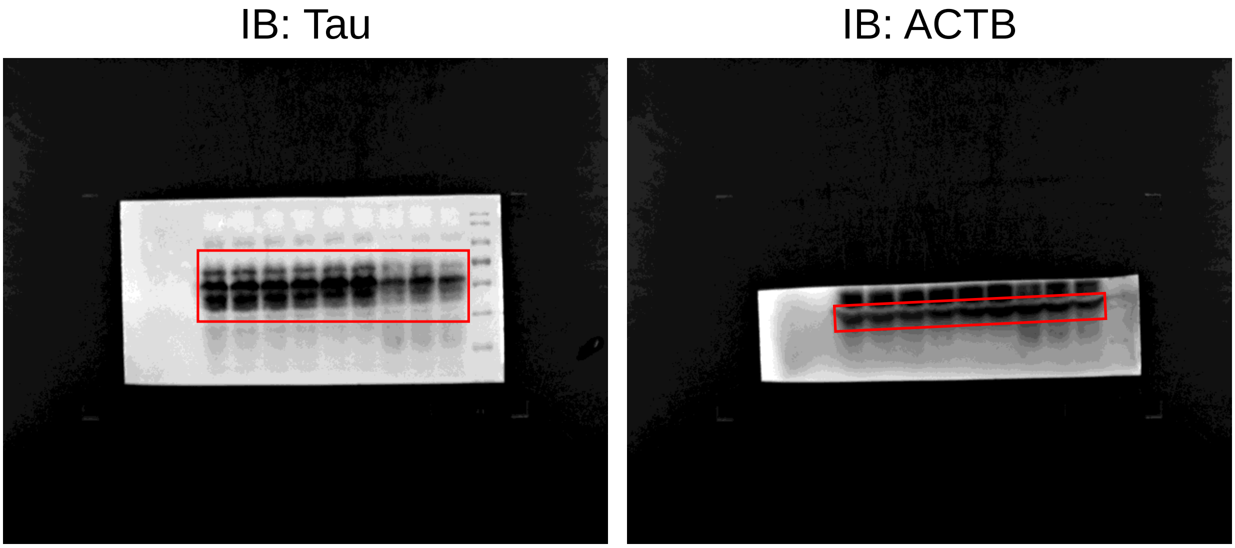

Fig. S5 Uncropped western blot images for Fig. 2q. Red boxes indicate cropped regions.

Fig. S6

IB: NeuN

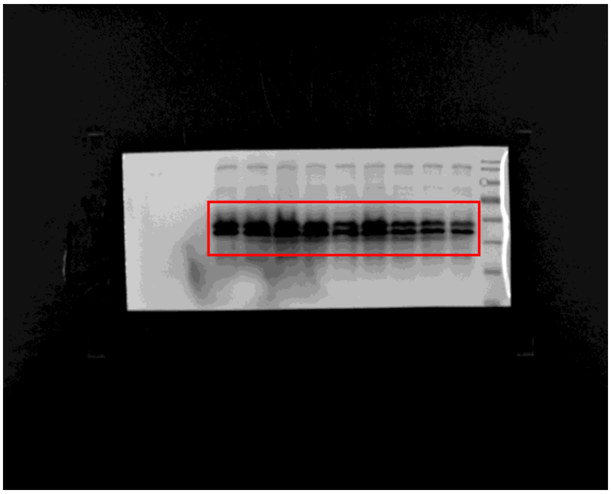

IB: GAPDH

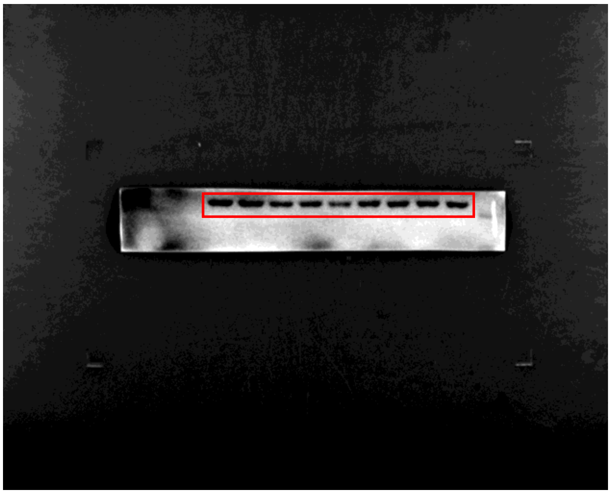

Fig. S6 Uncropped western blot images for Fig 2r. Red boxes indicate cropped regions.

Fig. S7

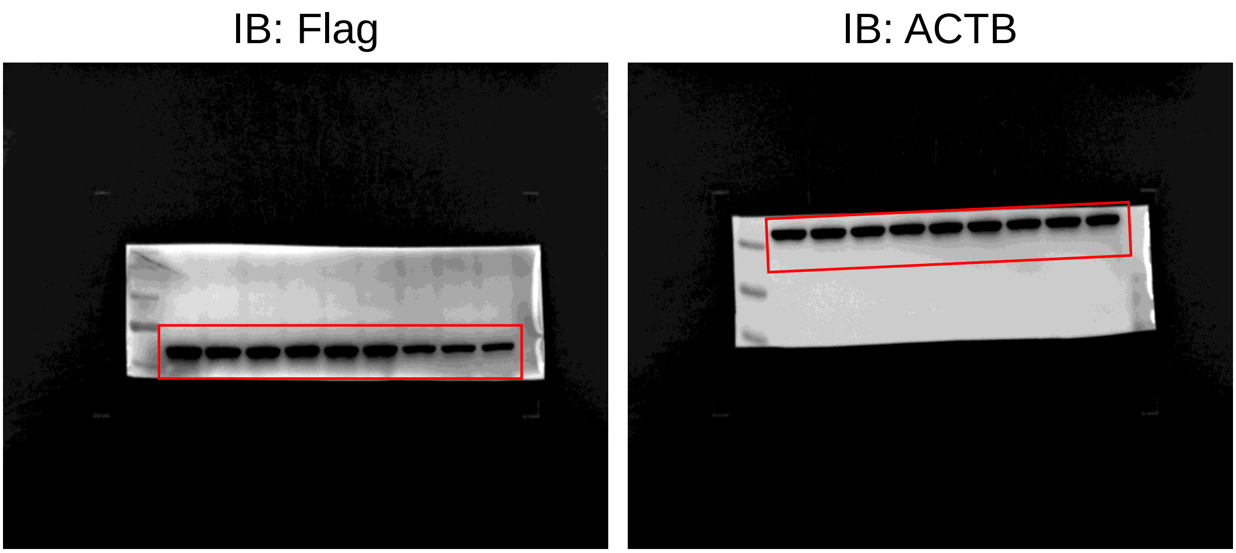

Fig. S7 Uncropped western blot images for Fig. 3e. Red boxes indicate cropped regions.

Fig. S8

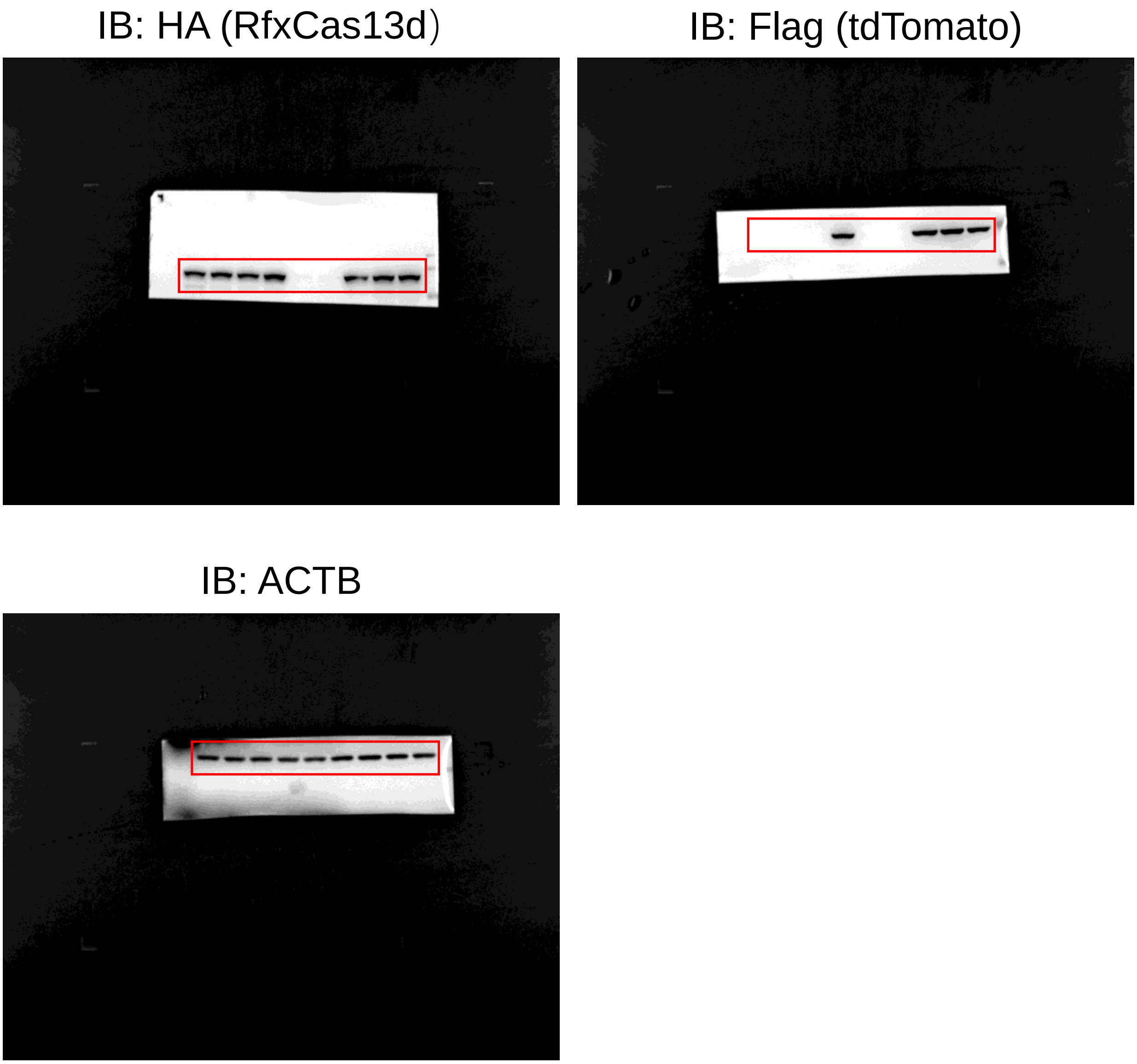

Fig. S8 Uncropped western blot images for Fig. 4a. Red boxes indicate cropped regions.

Fig. S9

IB: Puromycin

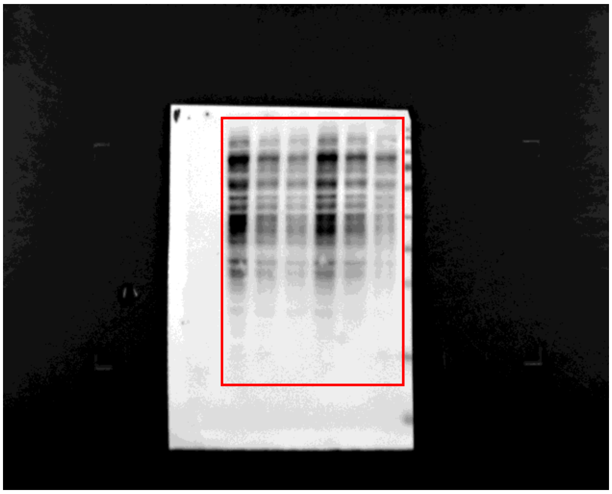

IB: Flag (tdTomato)

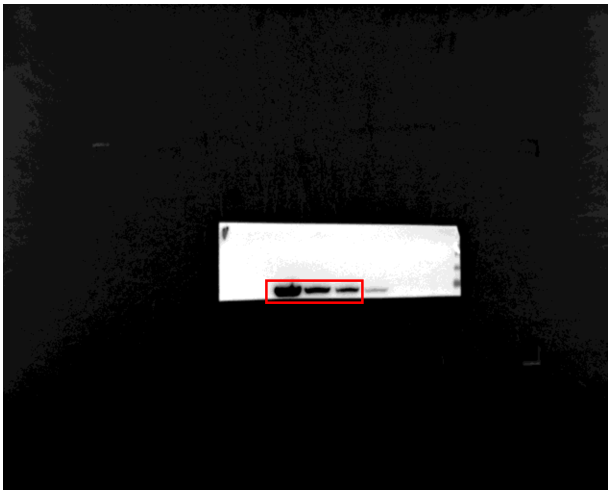

IB: HA (SIK3-S)

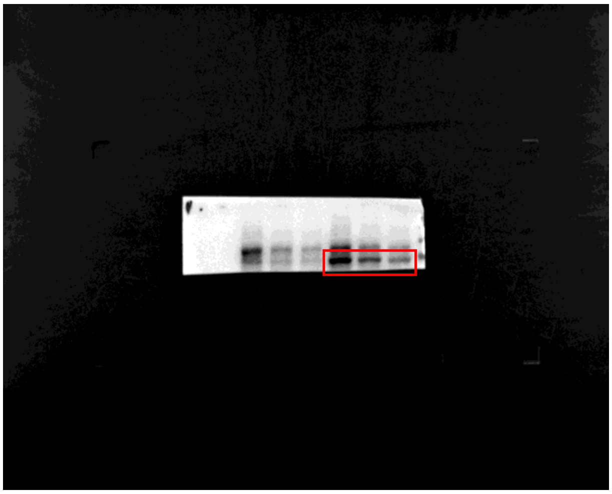

IB: TUBULIN

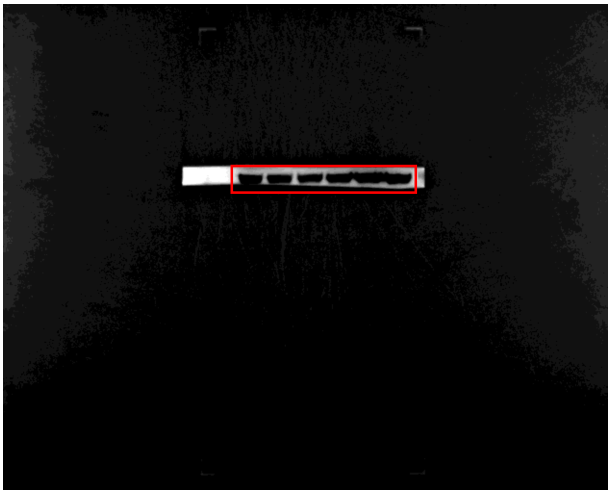

IB: GAPDH

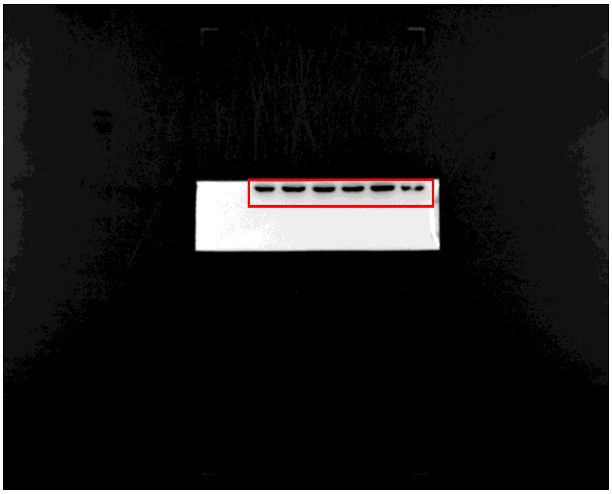

Comassie staining

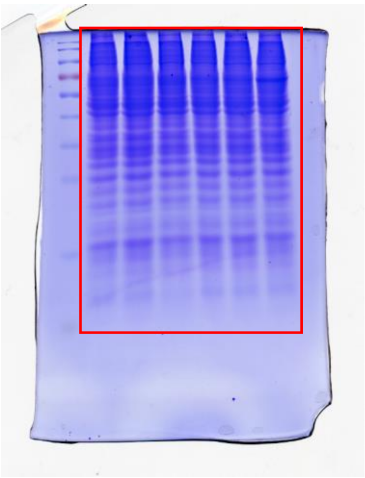

Fig. S9 Uncropped western blot images for Fig. 5c. Red boxes indicate cropped regions.

Fig. S10

IB: p-JNK

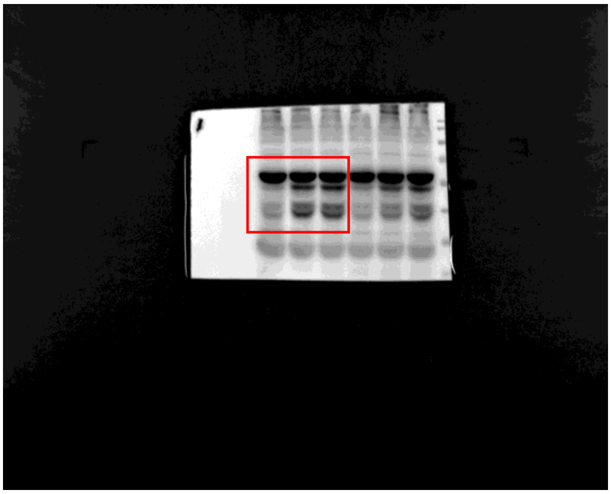

IB: p-p38

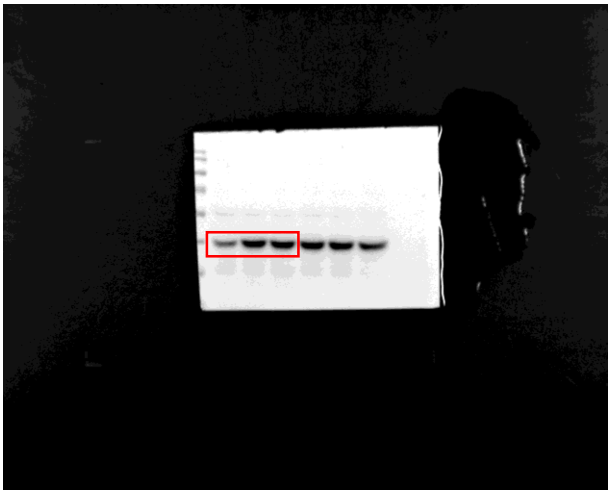

IB: p-ERK1/2

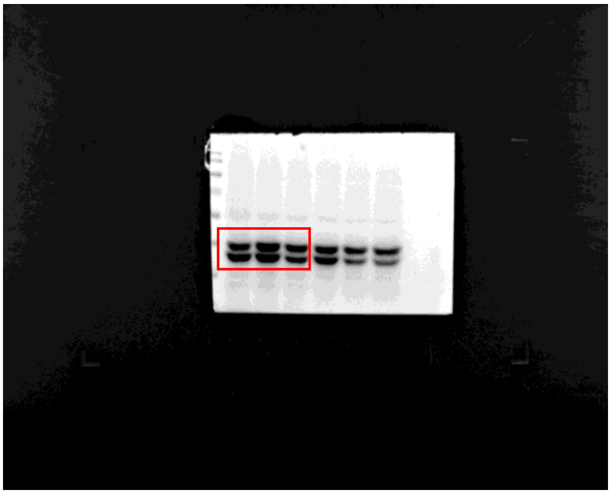

IB: Flag (tdTomato)

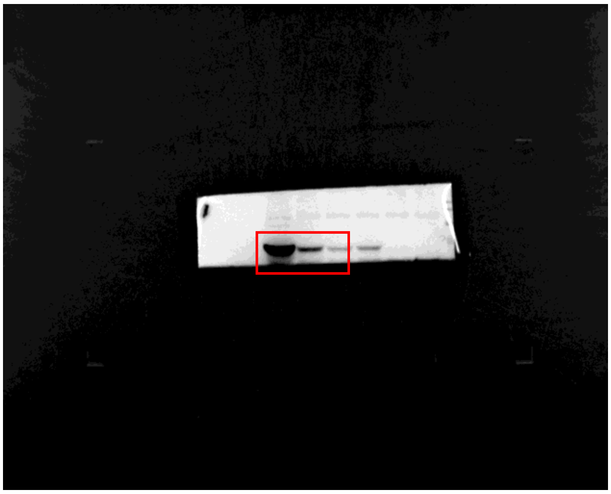

IB: ACTB

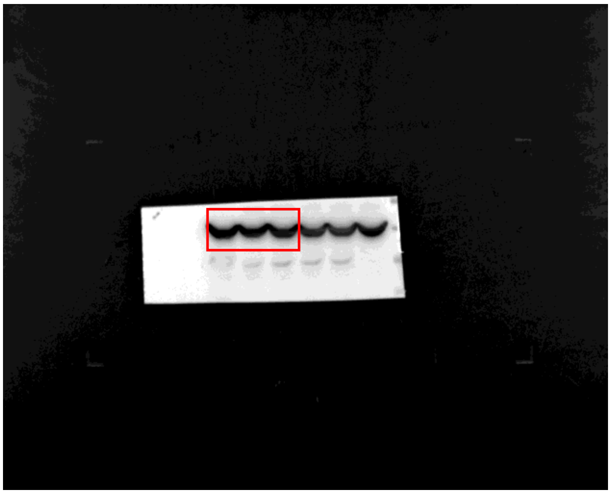

Fig. S10 Uncropped western blot images for Fig. 5h. Red boxes indicate cropped regions.

Fig. S11

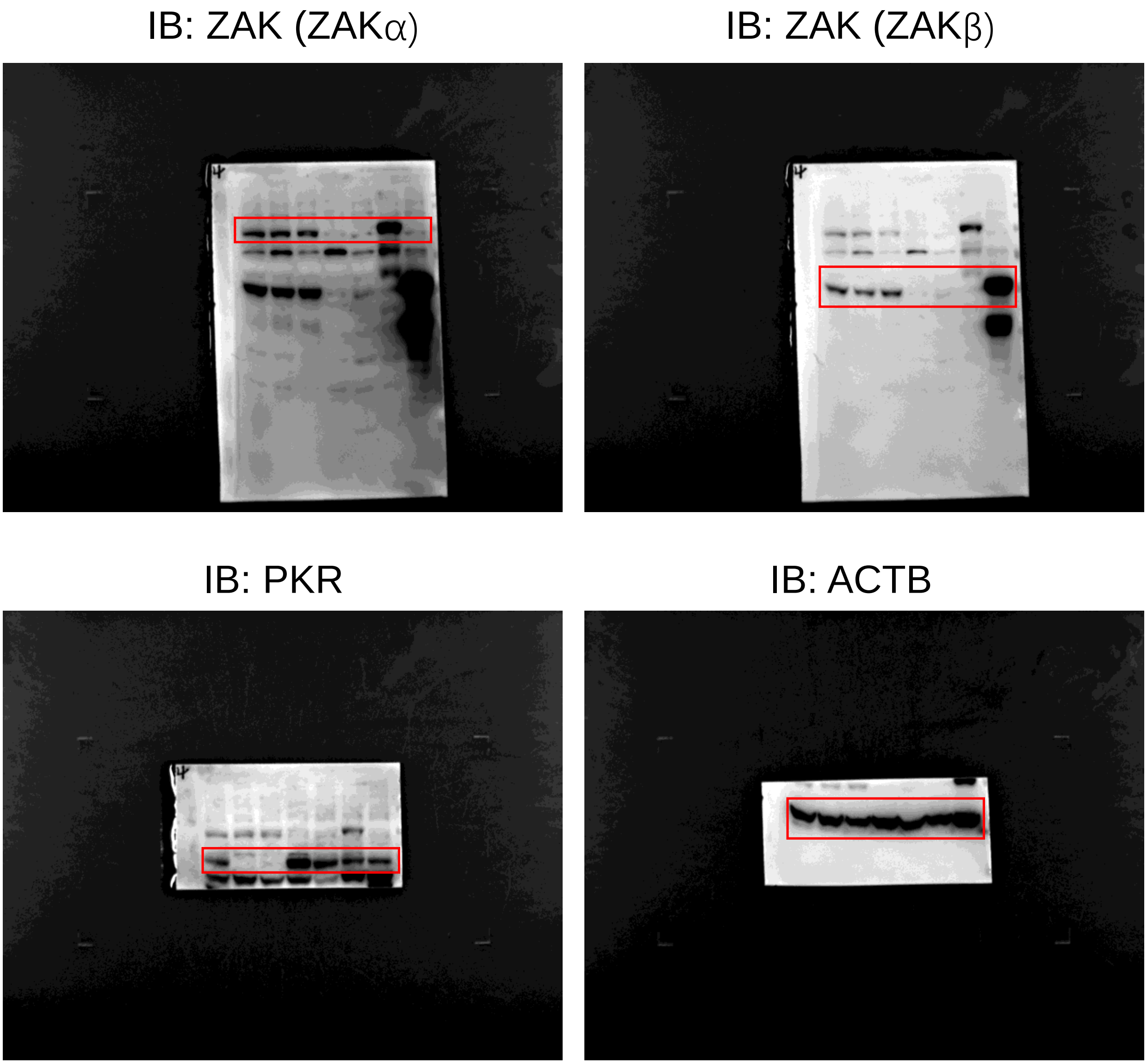

Fig. S11 Uncropped western blot images for Fig. 5k . Red boxes indicate cropped regions.

Fig. S12

IB: p-JNK

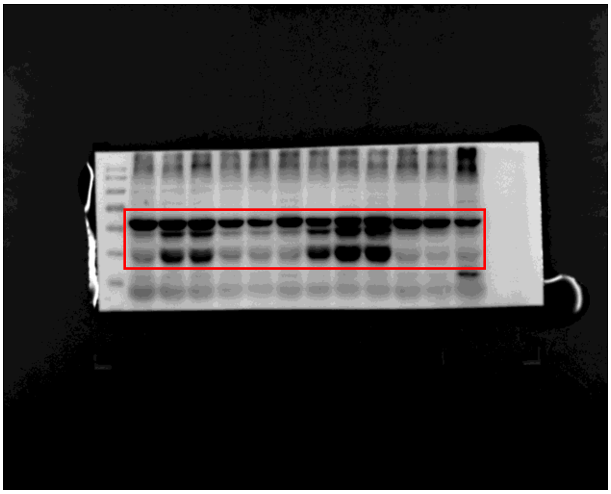

IB: p-p38

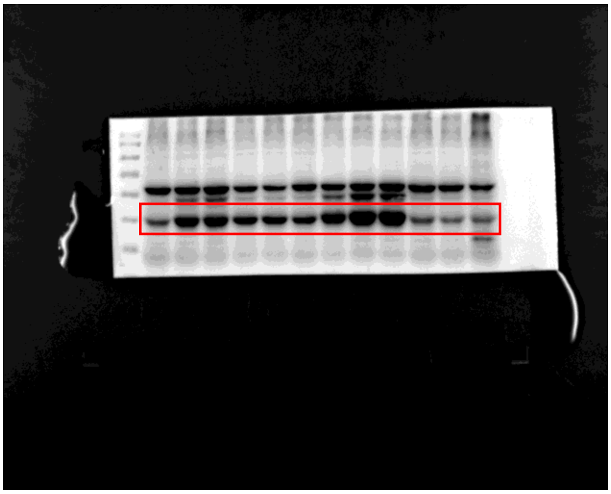

IB: Flag (tdTomato)

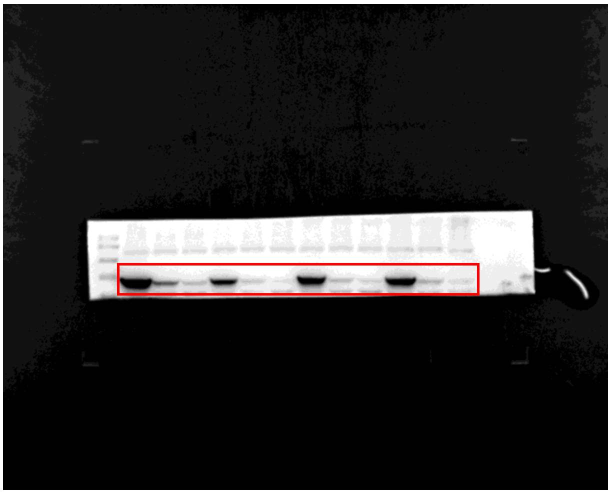

IB: ACTB

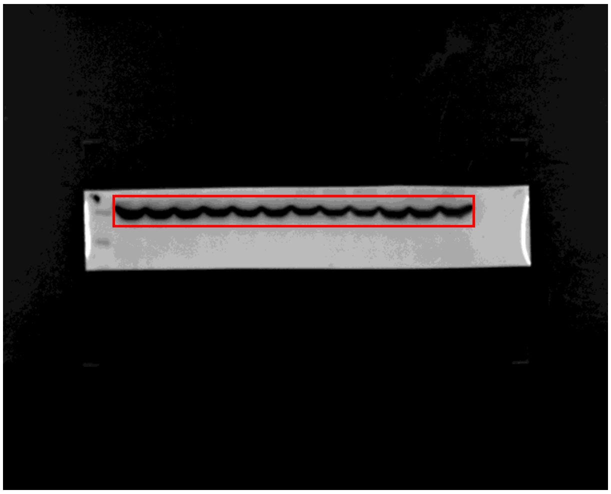

Fig. S12 Uncropped western blot images for Fig. 5I . Red boxes indicate cropped regions.

Fig. S13

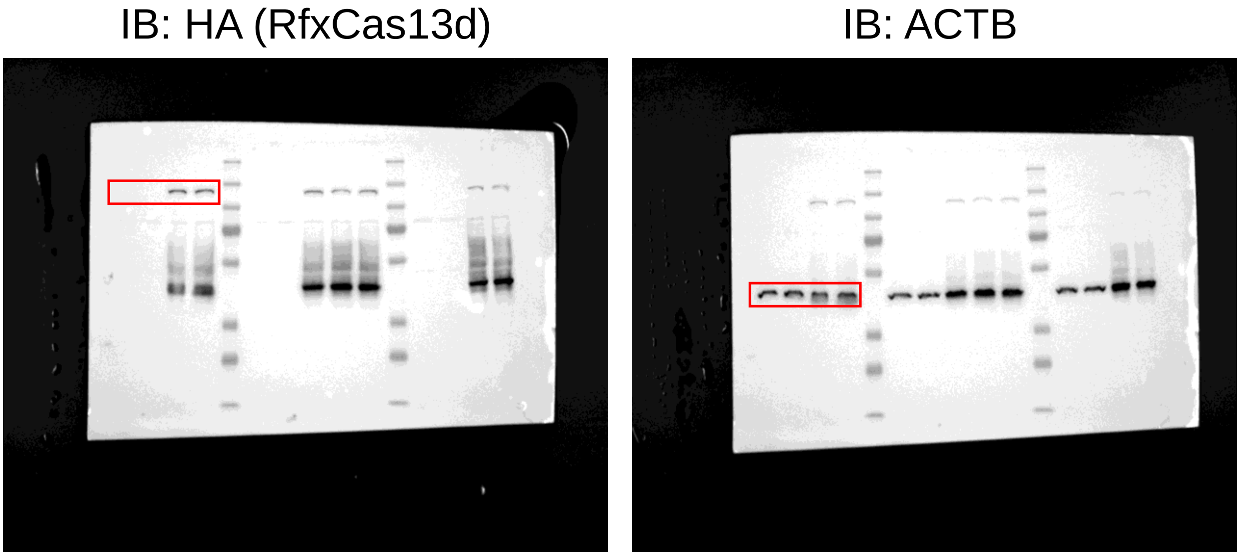

Fig. S13 Uncropped western blot images for Additional file 1: Fig. S3b. Red boxes indicate cropped regions.

Fig. S14

IB: HA (RfxCas13d)

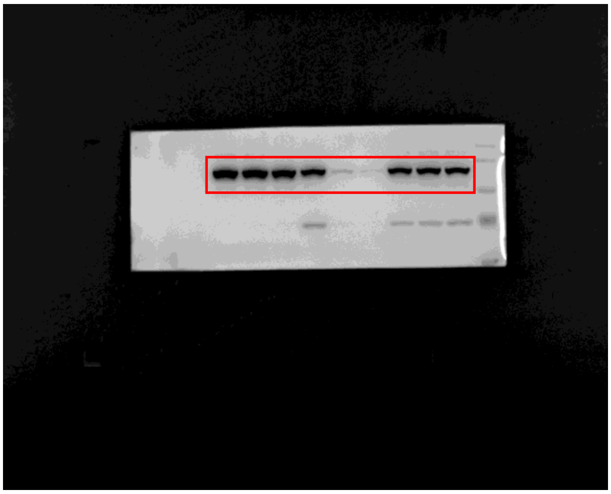

IB: HA (SIK3-S)

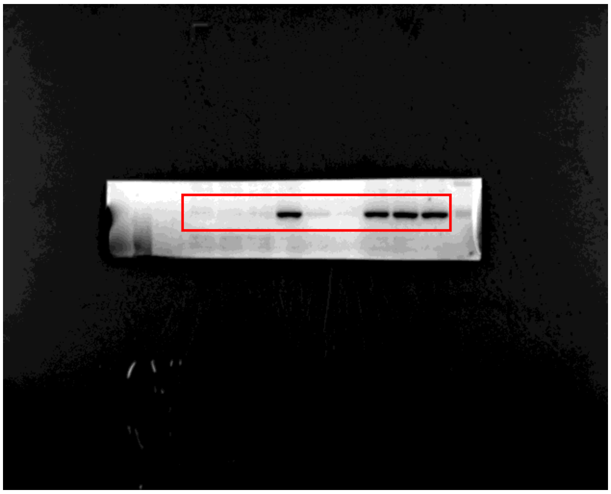

IB: ACTB

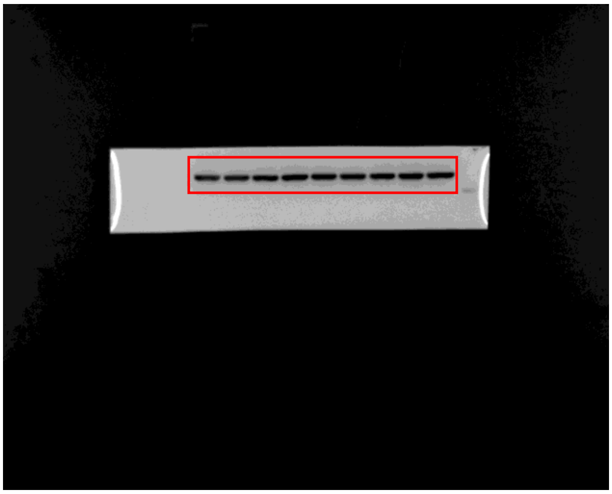

Fig. S14 Uncropped western blot images for Additional file 1: Fig. S7a. Red boxes indicate cropped regions.

Fig. S15

IB: Puromycin

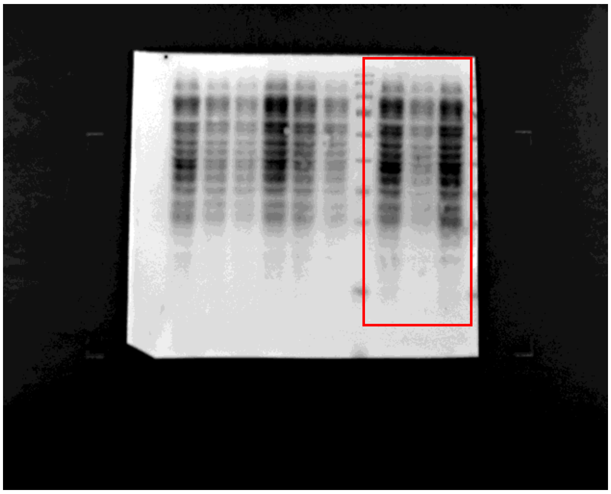

IB: HA (SIK3-S/SIK3-S-K37M)

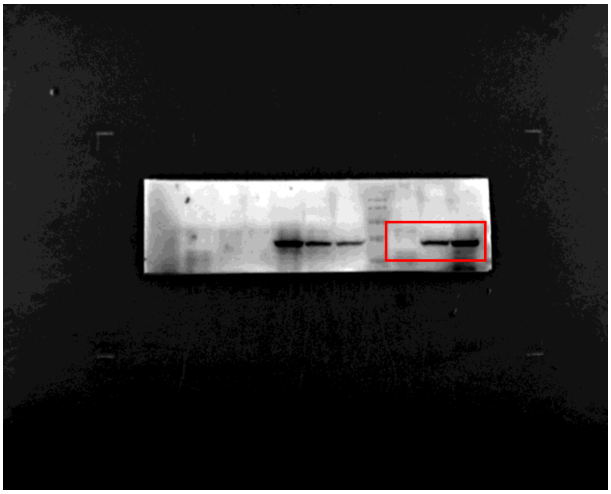

IB: ACTB

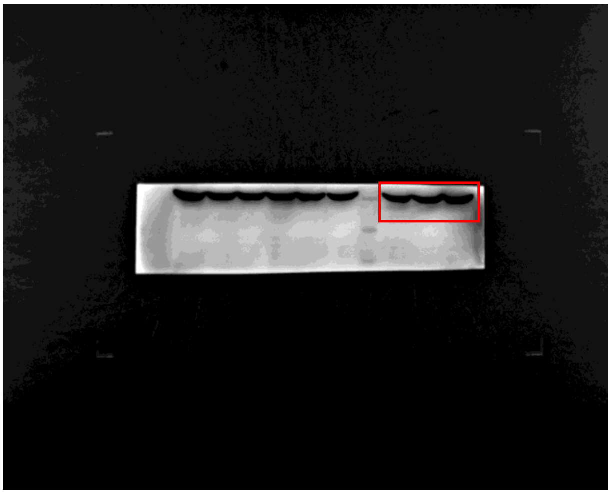

Fig. S15 Uncropped western blot images for Additional file 1: Fig. S9a. Red boxes indicate cropped regions.

Fig. S16

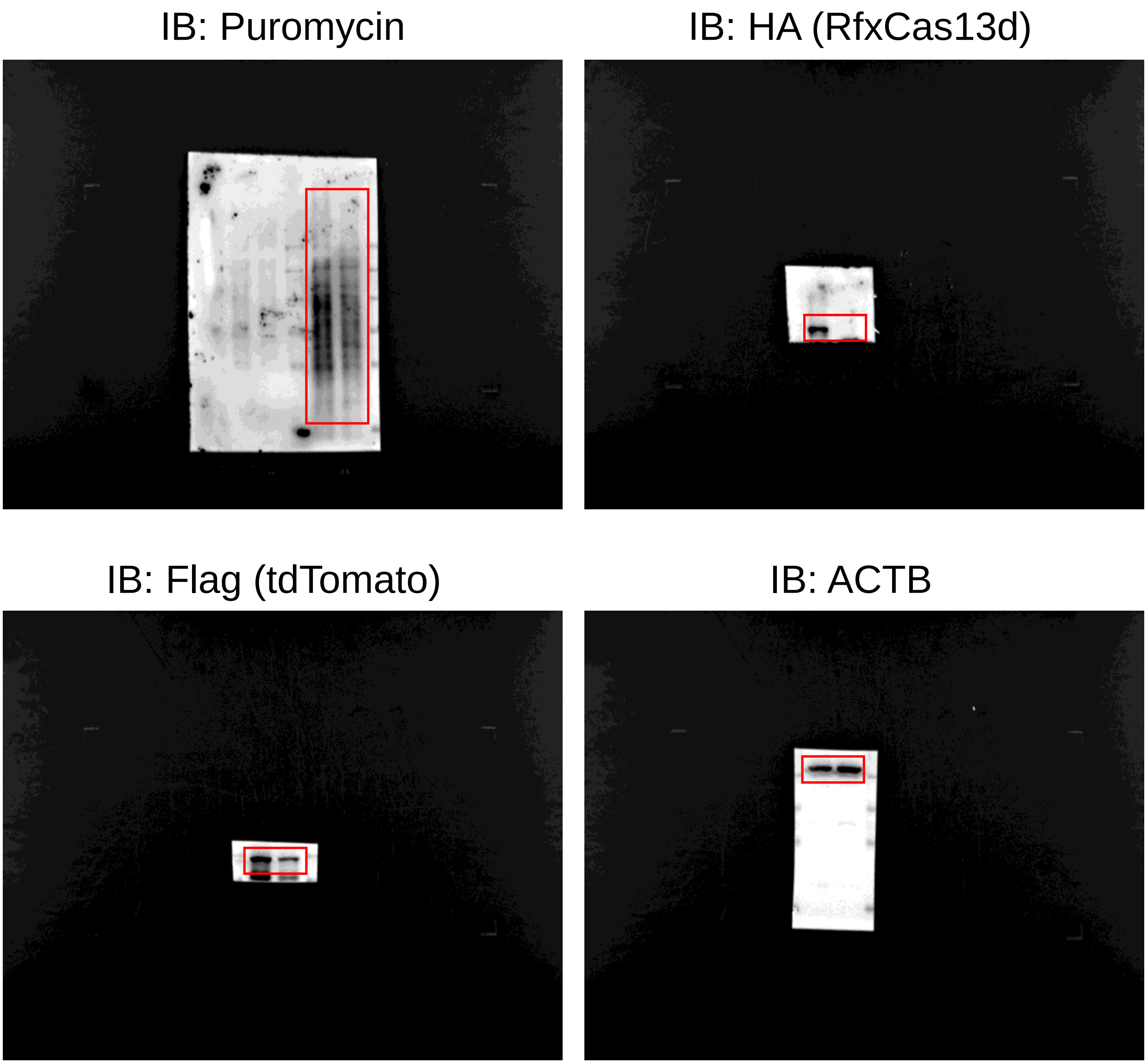

Fig. S16 Uncropped western blot images for Additional file 1: Fig. S11p. Red boxes indicate cropped regions.

Fig. S17

IB: Puromycin

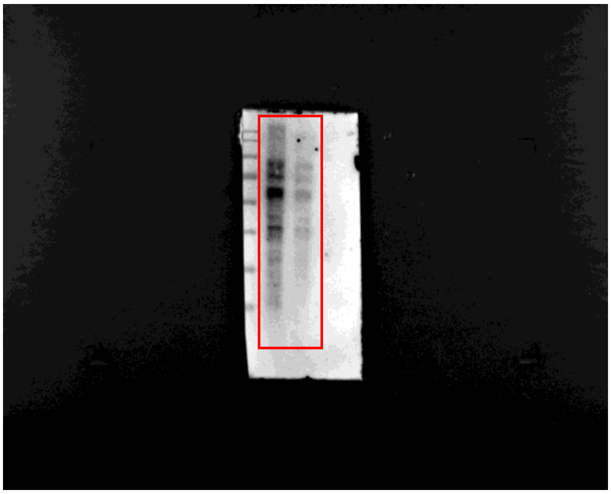

IB: HA (RfxCas13d)

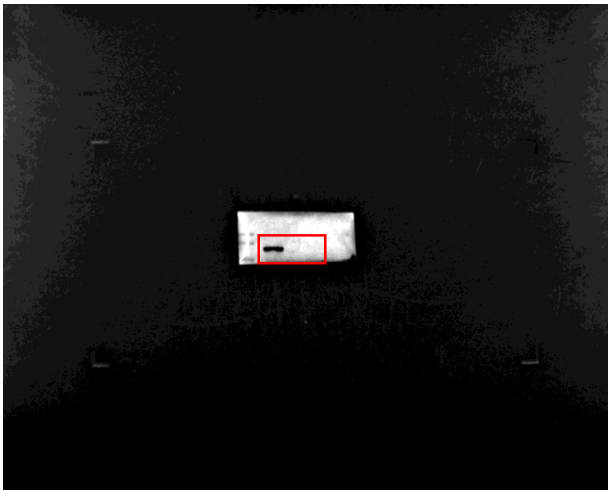

IB: Flag (tdTomato)

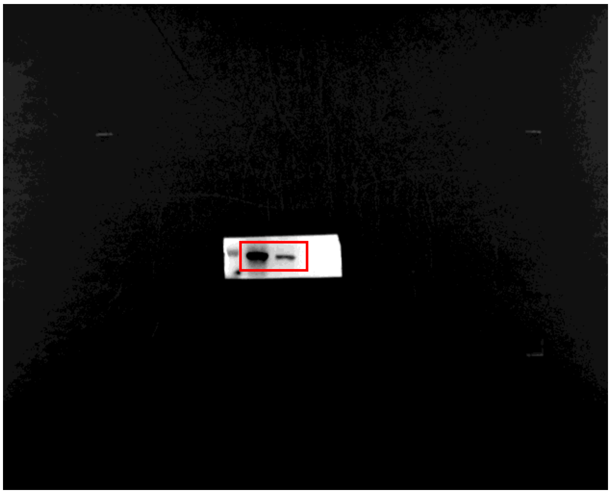

IB: ACTB

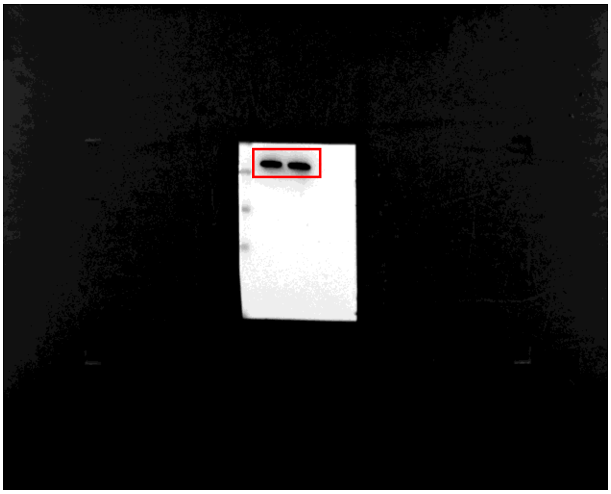

Fig. S17 Uncropped western blot images for Additional file 1: Fig. S11q. Red boxes indicate cropped regions.

Fig. S18

IB: Puromycin

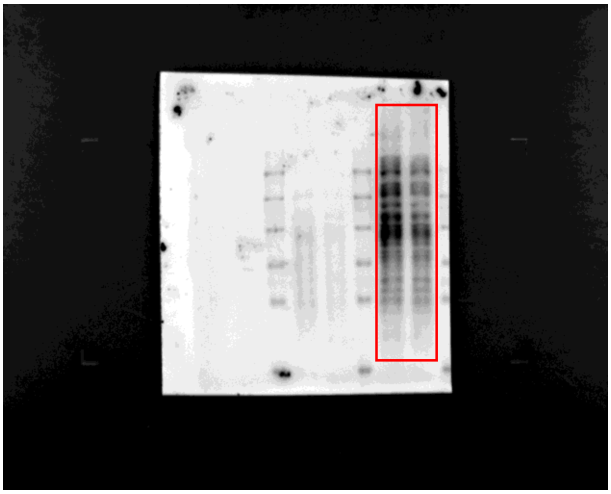

IB: HA (RfxCas13d)

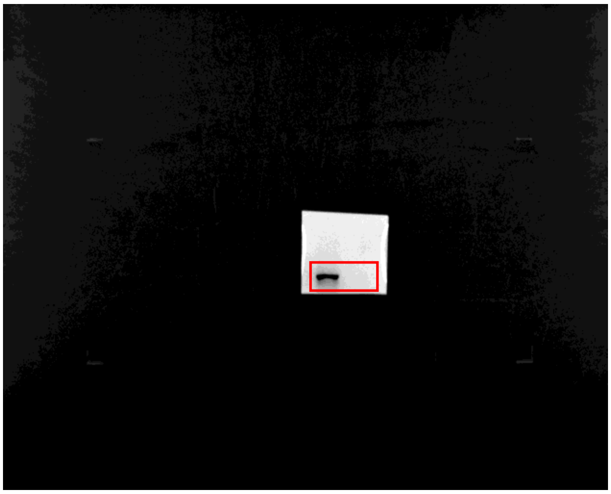

IB: Flag (tdTomato)

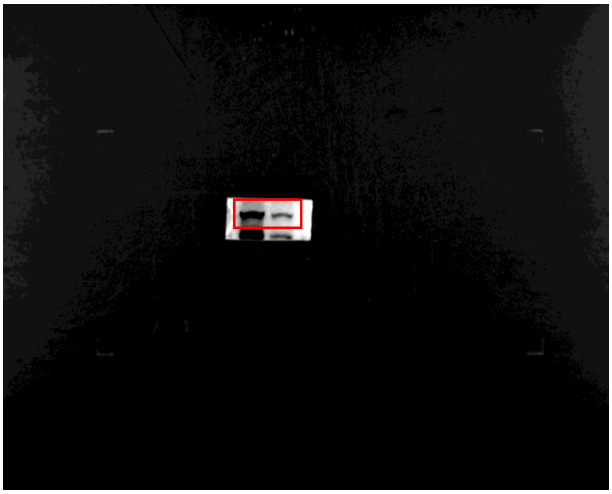

IB: ACTB

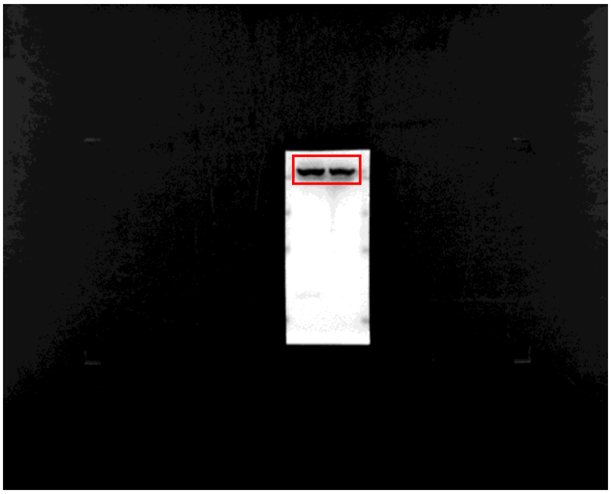

Fig. S18 Uncropped western blot images for Additional file 1: Fig. S11r. Red boxes indicate cropped regions.

Fig. S19

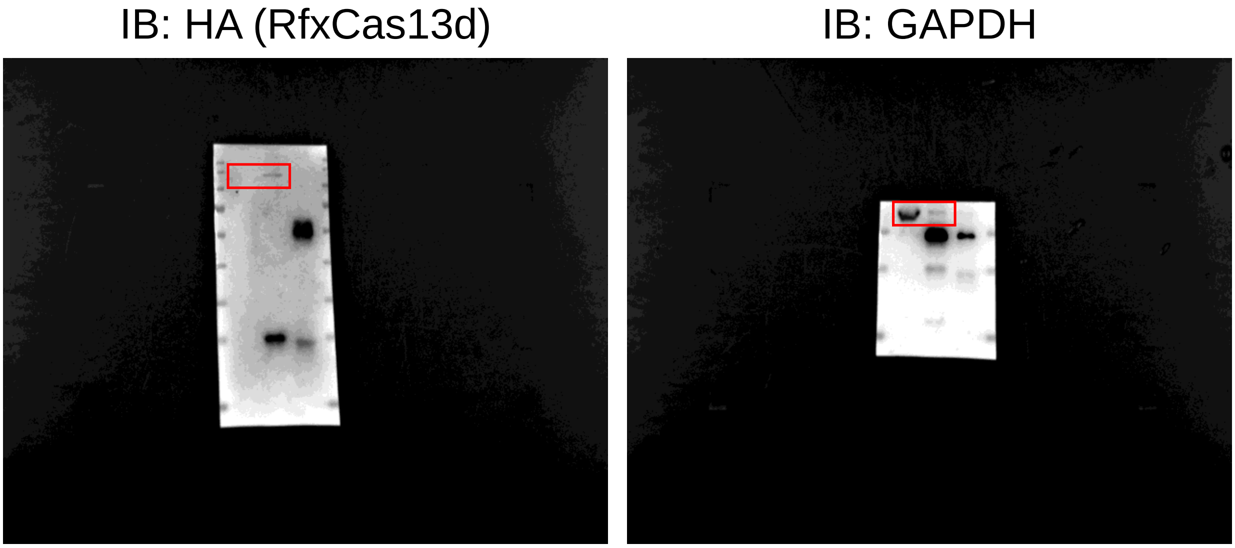

Fig. S19 Uncropped western blot images for Response Fig. 1a (First revision). Red boxes indicate cropped regions.

Fig. S20

IB: HA (RfxCas13d)

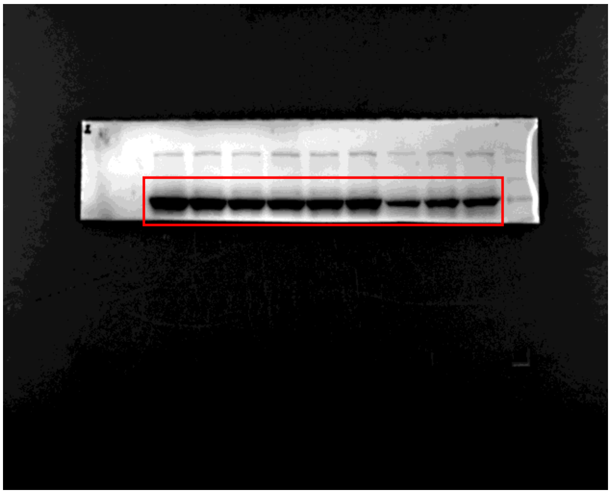

IB: GAPDH

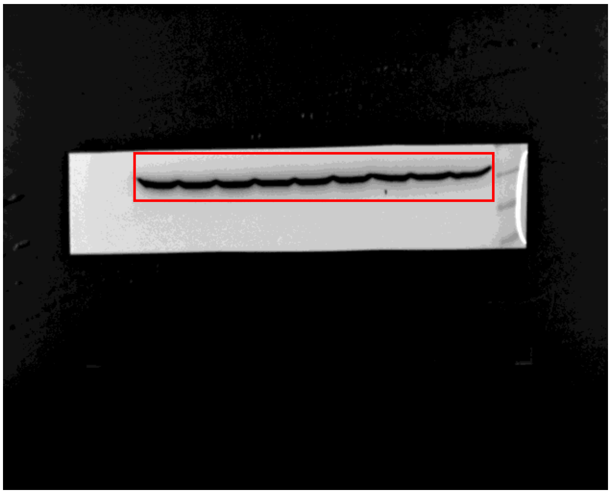

Fig. S20 Uncropped western blot images for Response Fig. 4a (First revision). Red boxes indicate cropped regions.

Fig. S21

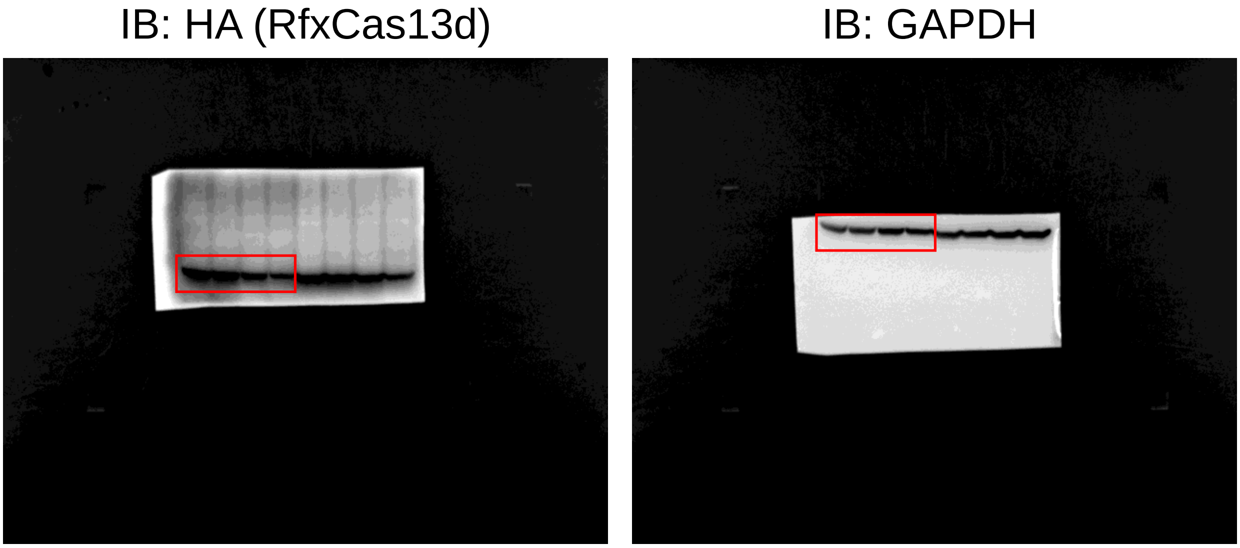

Fig. S21 Uncropped western blot images for Response Fig. 9a (First revision). Red boxes indicate cropped regions.

Fig. S22

IB: HA (RfxCas13d)

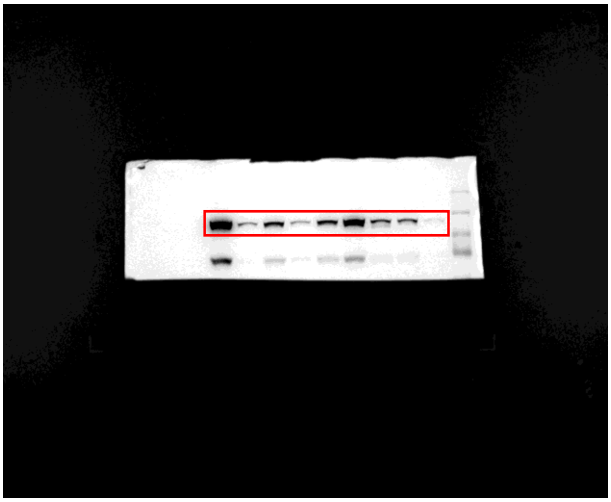

IB: HA (SIK3-S)

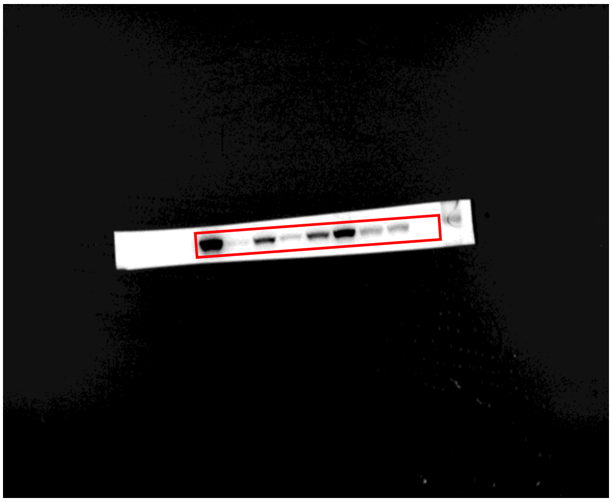

IB: ACTB

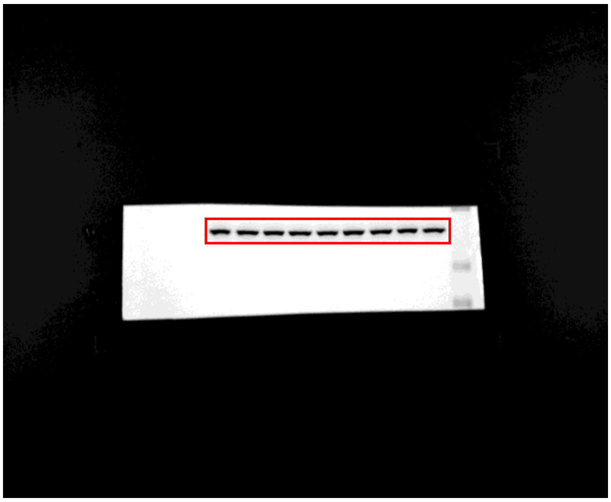

Fig. S22 Uncropped western blot images for Response Fig. 3b (Second revision). Red boxes indicate cropped regions.

Fig. S23

IB: HA (RfxCas13d)

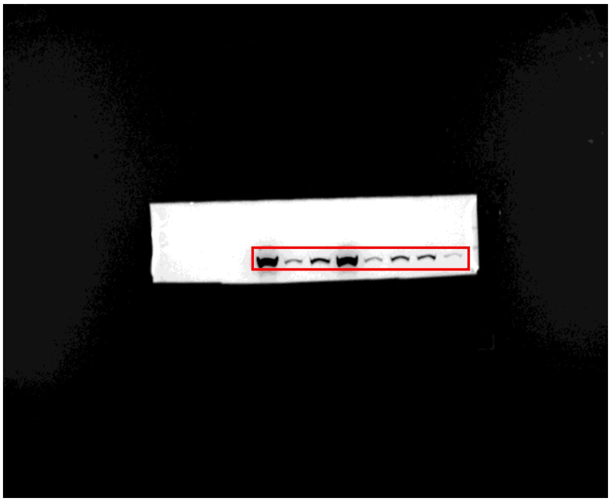

IB: Flag (tdTomato)

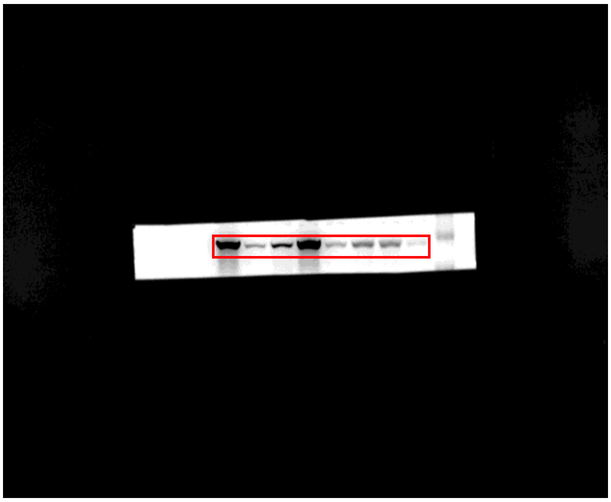

IB: ACTB

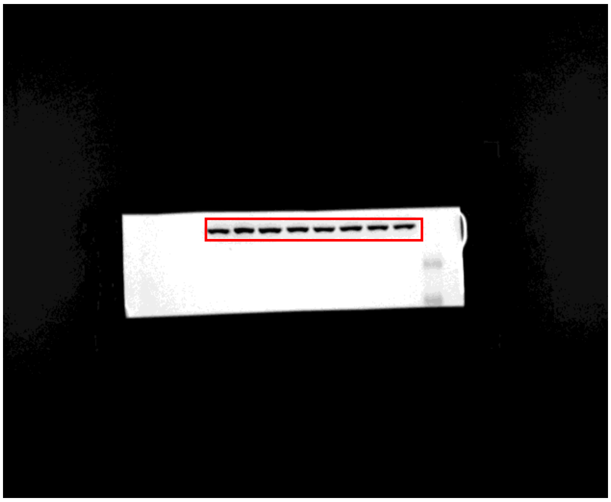

Fig. S23 Uncropped western blot images for Response Fig. 3c (Second revision). Red boxes indicate cropped regions.
