# Supplementary material for: The collateral activity of RfxCas13d can induce lethality in a RfxCas13d knock-in mouse model
Source: Genome Biol. 2023 Feb 1;24:20. doi: 10.1186/s13059-023-02860-w (PMC9893547; doi:10.1186/s13059-023-02860-w)
Supplement: Supplementary file 11 — Additional file 11. Review history. [file 13059_2023_2860_MOESM11_ESM.docx]

**First round of review**

**Reviewer reports:**

**Reviewer #1:** Cas13 technology has the potential to enable a range of gene silencing applications, including potentially for gene therapy. Interestingly, while Cas13 effectors display collateral targeting of non-target RNA when activated in bacterial cells, several studies that initially characterized some of these variants reported no such effect in mammalian cells. However, other groups in various settings have reported otherwise, observing instead that collateral targeting can manifest in mammalian cells but, curiously, not in all contexts.

The current study by Li et al. is interesting because it observes in vivo toxicity, a serious issue, potentially attributable to Cas13 collateral targeting. The authors in particular observe that collateral targeting is related to transcript concentration and that RfxCas13d can cleave 28s rRNA.

These are interesting results that are important for the field and can help guide the maturation of this technology. I do have several concerns that must be addressed. In particular, the authors did not provide convincing evidence for 28s rRNA degradation in vivo as source of the toxicity (or any other mechanism) and I have questions about the artificiality of the in vivo findings stemming from the LSL overexpression, among other points.

**Major comments:**

1. There is a disconnect between the In vivo toxicity results and the in vitro validation studies (and this is the case all throughout the manuscript). When targeting Sik3-S in mice, collateral targeting is observed, but when characterizing crRNAs in N2A cells, RNA-seq revealed specific knockdown, according to the authors in the text. One would expect observing the collateral targeting in the N2A background. Is this a product of the normalization to the non-targeted crRNA? Does the same result hold when compared to PBS treated cells? In addition, how abundant is Sik3-S mRNA in N2A cells and in the mouse brain, for that matter?

2. Did the authors observe any difficulties in establishing the LSL-RfxCas13d mouse line or in the viability of the colonies? Did these mice breed effectively? More detailed molecular genetics on the characterization of this mouse line are also required (how many copies, location, did it disrupt a gene, etc.). These are important points that can influence the conclusions from the results.

3. There does not appear to be any histological information on RfxCas13d expression in the brain or the extent of AAV-sgRNA delivery to the brain.

4. In Fig. 1G, the authors report detecting knockdown only with crRNA 8, however, it appears this is an analysis of whole brain. Did the authors analyze knockdown in specific areas known to be more effectively transduced by AAV-PHP.eB?

5. On a related note, did the authors confirm knockdown in the brain by qPCR?

6. How attributable is the observed toxicity to LSL overexpression? Is toxicity also observed when RfxCas13d targets the same transcripts but is delivered to WT mice via AAV? (Previously, this was reported to not cause toxicity). In addition, what is the toxic threshold of expression? Putting these results in context with the field suggest that RfxCas13d expression can also play a role in in vivo toxicity.

6. Comparing the western blots from 1G to 2A, the knockdown with RfxCas13d/crRNA-8 is not nearly as strong as with Cre, which was also delivered by AAV-PHP.eB, which begs the question, was RfxCas13d engaging with another target potentially? Or was collateral targeting affecting the total amount of protein in the tissue, causing a massive underestimation of knockdown? Did the authors observe any differences in total RNA or total protein between the RfxCas13d-injected mice and the negative control mice?

7. Similar point as in point 1 but for the RNA-seq results for Map2, Tau and NeuN as for Sik3-S. Why was RNA-seq showing specific knockdown but the collateral targeting (from non-target transcript knockdown) observed in vivo? Is this related to LSL used to express RfxCas13d in the mice? The disconnect here is concerning to me. The authors should conduct RNA-seq on the in vivo tissue and repeat RNA-seq in N2A cells using a promoter that matches the strength of expression from the in vivo studies. (In general, I wonder if the RNA-seq experiments in N2A cells are relevant, as the authors noted that NeuN is poorly expressed. Since there is disagreement with the in vivo studies, what is the predictive value in these measurements?)

8. I am confused by the conclusions from the section "RfxCas13d mediated lethality was not caused by off-target effects." When delivering a tdTomato-targeting crRNA, the authors observed toxicity in mice bred to express RfxCas13d and tdTomato, suggesting collateral activity was activated after targeting tdTomato. The fact that no toxicity was observed in mice LSL-RfxCas13d mice injected with the tdTomato-targeting crRNA is expected, because there was no target to activate collateral cleavage. This experiment does not disprove the role of RfxCas13d-mediated off-target effects and in fact supports that toxicity emerges only in the presence of the target transcript, which is still due to RfxCas13d and its "off-target effects."

9. When characterizing the role that transcript abundance has on collateral targeting in Fig. 4, the authors observe reduced RfxCas13d protein for some crRNA, but notably not all despite strong knockdown for all targets, indicating a crRNA dependence for this effect. I would appreciate if the authors could speculate on this. In particular, I wonder if the toxicity they observe in vivo can be attenuated by thorough crRNA screening, begging the question on what the result would be if they screened for Sik3-S crRNAs that did not reduce RfxCas13d expression.

10. The authors measured collateral targeting in 293 cells as detected by decreases in RfxCas13d protein, but how does this translate across the entire transcriptome? RNA-seq measurements would be very useful for this study,

11. I have concerns on the experimental design of the study beginning on Pg. 12. The authors stably express RfxCas13d but transfect the gene target. As the authors show, expression strength can influence the collateral targeting. I am concerned that the overexpression effects from this assay influenced the findings. Were similar effects observed when targeting endogenously expressed transcripts (low, medium and high thresholds)

12. Did the observe rRNA decreases in their in vivo experiments? There is currently no evidence suggesting a roel for ZAKalpha or 28s rRNA for the in vivo toxicity. What is the mechanism for toxicity then?

**Minor comments:**

1. The authors cite Zhuo et al. Cell 2020;181(3):590-603 e16. However, it should be noted that this has emerged as very controversial work. Beyond now several groups not being able to replicate its central reprogramming findings, one group was specifically unable to replicate its knockdown findings in vivo.

2. For additional evidence on the ability for RfxCas13d to target transcripts in the CNS, the authors should cite Powell et al. Sci Adv 2022 8, eabk2485 (2022). No severe adverse in vivo effects were observed in this work, though the authors did report that RfxCas13d silenced more non-target transcripts than previously suggested from the literature.

3. The authors should discuss the results by the Wilusz laboratory, which also observed that Cas13 collateral targeting may be due to transcript threshold (Nucleic Acids Res. 2022: https://doi.org/10.1093/nar/gkac159).

**Reviewer #2:** In this Manuscript, Li et al. have confirmed collateral RNA degradation from CRISPR-Cas13d system targeting either reporter or endogenous mRNAs in different mammalian cells. They, as other previously, showed this effect is more clearly observed when Cas13 targets are highly abundant. Finally, they described that the activity of RfxCas13d can cleave 28s rRNA triggering translation depletion and activation of the ZAKα-JNK/p38-immediate early gene (IEG) pathway. While the collateral activity trigger by Cas13d targeting on highly abundant mRNAs has been already shown in mammalian and drosophila cells (PMID: 35244715 and several biorxiv preprints: https://www.biorxiv.org/content/10.1101/2021.12.18.473271v1) this manuscript supports these results and partially characterizes the molecular effects induced by Cas13d collateral activity. This reviewer has, however, some important points that should be addressed.

**Major points:**

-The authors need to cite during all the manuscript the literature related with their findings. This reviewer can understand that preprints could not be cited (although I would strongly recommend it, especially when they support their results) but the recent paper published in NAR (PMID: 35244715) should be acknowledged and cited in all the statements regarding the collateral activity in mammalian cell culture including the findings about the more expression of the target the clearer the collateral. For example, in page 11: These data indicated that the collateral activity of RfxCas13d was triggered and positively correlated with the abundance of target RNA in mammalian cells, when targeting exogenous genes. There are multiples examples where this reference should be included. Thus, a complete revision of the manuscript is needed.

-Figure 2 g-i. It is great to see the transcriptomic data in these experiments but this reviewer wonders whether the collateral activity is detected in these experiments. Have the authors checked this? Similarly, authors showed RNAseq of Rbfox3 in cells with very low expression. Could it be more informative to show RNAseq data associated to panel 2f (western blot). And, is the collateral activity detected in Fig 2f conditions? Then, in the in vivo experiments (fig 2 j-l) it came to my attention that only 1 out the 2 gRNAs used triggers toxicity. Can the authors explain whether this can be due to the use of different gRNAs or little but still significant differences in RNA depletion can explain this. Is there a minimal threshold of the depletion to induce toxicity? For example, it is very intriguing in Fig 3a-d to see how with very similar level of depletion (by qPCR and RNAseq) of 2 independent gRNAs one triggers toxicity in vivo and the other not. Authors should discuss these results

-Fig S4b is confusing. This is aiming to show that LwaCas13a induces collateral activity. However, it only shows that LwaCas13a reduces GFP mRNA as fast as 4h post transfection of the in vitro synthesized crRNA and according to ribosomal data, no evidence of collateral activity is observed. Could it be that to trigger collateral activity a sustained expression of Cas13 and gRNA is needed? Indeed, the proliferation defects showed here and in other reports are clearly more evident at 48 h post transfection suggesting that an accumulation of the hyperactivity is needed to be physiologically relevant. Since the author have RNPs they could use them to test whether with the opposite kinetic of plasmid transfection the collateral effect is observed (high targeting after first hours post transfection and then degradation of the RNP vs higher activity along the accumulation of Cas13d and crRNA from plasmid expression).

-Fig 4Se-f, can the authors show qPCR or RNAseq associated to this panel? Since the collateral effect is on the RNA, this reviewer would recommend showing RNA targeting when possible.

-Fig 5. This is clearly the novelty of this manuscript, but it would be more convincing if the authors could demonstrate the molecular mechanism downstream the collateral activity in other mammalian cell line (beyond 293T) at least to see how general this response is.

-Statistical analysis should be done. There is only a section in methods where authors claimed to have done t-test to compare 2 groups, but it is not indicated in any figure. Moreover, when comparing more than 2 groups an Anova test is required at least. Figure legends should include this information.

**Minor points:**

-Methods should be better explained in general. It is not clear, for example, what Cas13 versions are used during the whole manuscript, how GFP crRNA in vitro synthesized is generated (in vitro transcription?), how plasmids were constructed (just mentioning standard molecular biology techniques is not sufficient, especially when the plasmids used in the manuscript are not properly detailed). Please, review the whole section to make the manuscript more transparent.

-Discussion: pag 17 (345-47), authors could speculate a bit how to reduce collateral activity. A couple of preprints have been recently deposited about it.

-Some statements are too overweight. For example, in results: These results demonstrated 275 that RNA structure and RBPs were involved in protecting RNA. Suggest looks more accurate. Or This explained why in Fig. 4a, changes in protein levels of RfxCas13d were more obvious than changes in RNA levels. This may/could explain would fit better.

-Authors stated: transfection of plasmids encoding LwaCas13a, PspCas13b or RfxCas13d into U87 cells, regardless of with targeting or NT crRNA, would affect cell viability, indicating that cell viability changes have nothing to do with collateral activity. I do not see this in the paper authors cited. For example, Fig 4e of this paper (Ozcan et al., 2021) shows that with NT crRNA no toxicity is observed using Cas13d. Please review this properly and change or remove the statement.

-Page 6 lines line 122 there is a wrong figure called

-Page 3 lines 59: Surprisingly, binding of the target RNA to Cas13-crRNA complex also activates a nonspecific RNase activity, which promiscuously cleaves bystander RNAs without… As far as this reviewer knows the collateral effect needs RNA cut to be triggered and not only Cas13 binding to the RNA. Please correct the sentence accordingly.

**Author response:**

Dear Dr. Pang

Thank you very much for your letter dated June 15, 2022. My co-authors join me in expressing our sincere appreciation to you for the time and efforts in handling the above referenced manuscript (GBIO-D-22-00216). We also thank the reviewers for their constructive comments to improve our manuscript. We have performed experiments and revised the manuscript to address those concerns raised by the reviewers. The point-by-point responses to these comments are listed below.

**Response to Reviewer #1:**

**Major comments:**

1. There is a disconnect between the In vivo toxicity results and the in vitro validation studies (and this is the case all throughout the manuscript). When targeting Sik3-S in mice, collateral targeting is observed, but when characterizing crRNAs in N2A cells, RNA-seq revealed specific knockdown, according to the authors in the text. One would expect observing the collateral targeting in the N2A background. Is this a product of the normalization to the non-targeted crRNA? Does the same result hold when compared to PBS treated cells? In addition, how abundant is Sik3-S mRNA in N2A cells and in the mouse brain, for that matter?

7. Similar point as in point 1 but for the RNA-seq results for Map2, Tau and NeuN as for Sik3-S. Why was RNA-seq showing specific knockdown but the collateral targeting (from non-target transcript knockdown) observed in vivo? Is this related to LSL used to express RfxCas13d in the mice? The disconnect here is concerning to me. The authors should conduct RNA-seq on the in vivo tissue and repeat RNA-seq in N2A cells using a promoter that matches the strength of expression from the in vivo studies. (In general, I wonder if the RNA-seq experiments in N2A cells are relevant, as the authors noted that NeuN is poorly expressed. Since there is disagreement with the in vivo studies, what is the predictive value in these measurements?).

**Response:** We apologize for the confusion caused by our unclear statements. The purpose of our RNA-seq analysis in N2a cells was to verify the knockdown efficiency and specificity of crRNAs. Our results showed that compared with NT crRNA group, these target genes were significantly knocked down in the targeting crRNA groups, while the expression levels of most other genes remained unchanged. This did not suggest that there were no other differentially expressed genes (DEGs) other than the target genes. We analyzed and found approximately 150-600 DEGs (P adj < 0.05 and change fold ≥2) per targeting crRNA compared to NT crRNA (Response Table 1/Table S1 of the final manuscript). These DEGs may result from the loss of target genes, collateral activities, off-target effects or other reasons. Because of the low expression levels of these target genes, they only triggered weak collateral activities (Response Fig. 1a). And previous studies reported that the collateral activity of Cas13 was undetected by RNA-seq upon targeting lowly-expressed endogenous genes(1-4). Moreover, a recent published study and multiple preprint studies showed that the collateral activity of RfxCas13d was positively corrected with the abundance of target RNAs, and the collateral activities were too weak to be detected upon targeting lowly-expressed genes(5-8). Therefore, although we observed untargeted DEGs, we could not directly conclude which DEGs were attributable to the collateral activities. Since the expression levels of these target genes are lower in N2a cells than in brain neurons (Response Fig. 1a), and the substantial intrinsic differences between brain neurons and N2a cells, we think that RNA-seq analysis in N2a is poorly predictive of or less-relevant with the *in vivo* toxicity.

In addition, how abundant is Sik3-S mRNA in N2A cells and in the mouse brain, for that matter?

**Response:** As shown in Response Figure 1a, the expression levels of these target genes are higher in mouse brains (from bulk RNA-seq analysis of brains) than in N2a cells. Since Map2, Tau and NeuN are well-characterized neuron marker genes and a previous study showed that Sik3 is highly expressed in brain neurons(9), these target genes are expressed at higher levels in brain neurons than values measured by bulk RNA-seq analysis.

Is this a product of the normalization to the non-targeted crRNA? Does the same result hold when compared to PBS treated cells?

**Response:** These DEGs were obtained by comparing the targeting crRNA groups with the NT crRNA group, which is consistent with our animal experiments.

Is this related to LSL used to express RfxCas13d in the mice?

**Response:** We think that this is not related to LSL-mediated RfxCas13d expression. Please refer to the answer to the sixth comment.

The authors should conduct RNA-seq on the in vivo tissue and repeat RNA-seq in N2A cells using a promoter that matches the strength of expression from the in vivo studies.

**Response:** We agree. Initially, we performed bulk RNA-seq analysis of whole brains to study the *in vivo* toxicity caused by RfxCas13d, but could not obtain useful information because various cell types (including cells that did not express RfxCas13d) were included (Response Table 2/Table S7 of the final manuscript). Due to the difficulty of dissociating adult mouse brain tissue and obtaining intact neuronal cells, we were unable to perform RNA-seq analysis of these AAV-PHP.eB-infected neurons alone.

The expression level of RfxCas13d in mouse brain is too low to be directly detected and the extremely weak band of RfxCas13d-HA could only be detected through enrichment using anti-HA magnetic beads (Response Fig. 1b). Therefore, the expression level of RfxCas13d is much lower in brain neurons than in N2a cells.

**Response Figure 1**. **a.** The table to show the expression levels (FPKM) of indicated genes in N2a cells and mouse brain (data from RNA-seq analysis of N2a cells and bulk RNA-seq analysis of brains). **b.** Western blot to show the expression level of RfxCas13d and GAPDH in whole brain lysate and immunoprecipitated proteins using anti-HA magnetic beads.

2. Did the authors observe any difficulties in establishing the LSL-RfxCas13d mouse line or in the viability of the colonies? Did these mice breed effectively? More detailed molecular genetics on the characterization of this mouse line are also required (how many copies, location, did it disrupt a gene, etc.). These are important points that can influence the conclusions from the results.

**Response:** Thanks for your suggestions. We illustrated the procedure of generating ^LSL^RfxCas13d^fl/fl^ mice in more detail. We inserted the CAG-loxP-STOP-loxP-RfxCas13d cassette into the intron region of the *Rosa26* locus by homologous recombination using CRISPR/Cas9 technology and ensured that they have the expected genotype by genotyping (Response Fig. 2a-b/Revised Manuscript Fig. S1c-d). The *Rosa26* locus is commonly used to knock-in cDNA constructs for ubiquitous or conditional gene expression in transgenic mice, which is considered as one of the most well-established safe loci(10, 11). And the loxP-STOP-loxP (LSL) system restricts the expression of RfxCas13d only in the presence of Cre recombinase. Therefore, insertion of the CAG-LSL-RfxCas13d cassette at the *Rosa26* locus theoretically did not affect the viability of mice. Indeed, we did twice microinjections of embryos and obtained positive founders on both occasions. And we directly obtained a homozygous ^LSL^RfxCas13d^fl/fl^ mouse in the second experiment (Response Fig. 2c). Furthermore, we counted the numbers of different genotypes in six litters of mice (^LSL^RfxCas13d^+/fl^ x ^LSL^RfxCas13d^+/fl^) and found that they obey Mendel's laws of inheritance (Response Fig. 2d). These results suggested that insertion of CAG-LSL-RfxCas13d into the *Rosa26* locus did not affect mouse viability. And we did not observe any difference in behavior and reproduction between ^LSL^RfxCas13d^fl/fl^ and wild-type (WT) mice.

**Response Figure 2**. **a.** Schematic illustration of the procedure of generating ^LSL^RfxCas13d mice. NLS: nuclear localization sequence; HA: hemagglutinin tag; CAG: CAG promoter; U6: U6 promoter; STOP: a tripartite transcriptional stop cassette. **b.** Genotyping of ^LSL^RfxCas13d^fl/fl^, ^LSL^RfxCas13d^+/fl^ and WT mice. **c.** Statistical table to show the results of twice embryo microinjections. **d.** Statistical analysis of the deviations from Mendelian genetics for six litters of mice (^LSL^RfxCas13d^+/fl^ x ^LSL^RfxCas13d^+/fl^). df: degree of freedom.

3. There does not appear to be any histological information on RfxCas13d expression in the brain or the extent of AAV-sgRNA delivery to the brain.

**Response:** We agree. Both previous study and our recent study have demonstrated that AAV-PHP.eB can efficiently cross the blood brain barrier and transduce the majority of neurons and astrocytes across the adult mouse brain(12, 13). Theoretically, crRNAs are expressed in all cells infected with AAV-PHP.eB, whereas the expression of RfxCas13d is dependent on Cre-mediated excision of the LSL cassette and is therefore only released in infected neurons. Since the expression level of RfxCas13d is too low to be detected, we could only use the histological information of Cre to indirectly indicate that of RfxCas13d. We collected the brains of ^LSL^RfxCas13d^+/fl^Ai14^+/fl^ mice 12 days after injection of AAV-PHP.eB carrying hSYN-driven Cre and U6-driven tdTomato crRNA 7 for immunohistochemistry (IHC). As shown by coimmunostaining of Cre and tdTomato below, Cre was expressed in the majority regions of brain (Response Fig. 3a), and the expression of tdTomato was knocked down, such as in the cortex, thalamus and hypothalamus (Response Fig. 3a-b).

**Response Figure 3**. **a.** Representative images showing coimmunostaining of Cre and tdTomato in the sagittal brain sections of ^LSL^RfxCas13d^+/fl^Ai14^+/fl^ mice 12 days after injection of AAV-PHP.eB carrying hSYN-driven Cre and U6-driven *tdTomato* crRNA 7. Scale bar: 1 mm. **b.** Representative images showing coimmunostaining of Cre and tdTomato in the cortex, thalamus and hypothalamus. White arrows indicate the cells expressing Cre but not tdTomato, indicating that the expression of tdTomato was knocked down in these cells. Scale bar: 20 μm.

4. In Fig. 1G, the authors report detecting knockdown only with crRNA 8, however, it appears this is an analysis of whole brain. Did the authors analyze knockdown in specific areas known to be more effectively transduced by AAV-PHP.eB?

**Response:** To the best of our knowledge, there is no available anti-SIK3-S antibody for immunohistochemistry (IHC). Therefore, IHC cannot be used to demonstrate the knockdown effects of RfxCas13d on SIK3-S in different brain regions. According to our recent study, the transduction efficiency of AAV-PHP.eB in the thalamus is higher than that of other brain regions we observed(12), and as shown in the figure above, the knockdown effect of RfxCas13d on tdTomato was also obvious in the thalamus (Response Fig. 3b). Therefore, we isolated the mouse thalamus from ^LSL^RfxCas13d^fl/fl^ mice 20 days after injection of AAV-PHP.eB carrying hSYN-driven Cre and U6-driven crRNA for western blot to validate the knockdown of SIK3-S. As shown below, SIK-S was obviously knocked down with Sik3-S crRNA 8 and only slightly knocked down with Sik3-S crRNA 1, compared with NT crRNA (Response Fig. 4a).

**Response Figure 4**. **a.** Western blot to measure the protein level of SIK3-S in the thalamus from ^LSL^RfxCas13d^fl/fl^ mice 20 days after injection of AAV-PHP.eB carrying hSYN-driven Cre and U6-driven indicated crRNAs.

5. On a related note, did the authors confirm knockdown in the brain by qPCR?

**Response:** We agree and examined the knockdown effect of RfxCas13d on *Sik3-S* *in vivo* by qPCR. As shown below, *Sik3-S* in mouse brain was significantly knocked down in *Sik3-S* crRNA 8 group, compared with NT crRNA group.

**Response Figure 5**. **a.** RT-qPCR to measure the mRNA level of *Sik3-S* in the brains of ^LSL^RfxCas13d^fl/fl^ mice 20 days after injection of AAV-PHP.eB carrying hSYN-driven Cre and U6-driven NT crRNA (n=3) or *Sik3-S* crRNAs (n=3). **a,** One-way ANOVA with Dunn’s multiple comparisons test. Significance levels are noted as **P < 0.01 or ns (P > 0.05). All values are presented as mean ± SEM.

6. How attributable is the observed toxicity to LSL overexpression? Is toxicity also observed when RfxCas13d targets the same transcripts but is delivered to WT mice via AAV? (Previously, this was reported to not cause toxicity). In addition, what is the toxic threshold of expression? Putting these results in context with the field suggest that RfxCas13d expression can also play a role in in vivo toxicity.

**Response:** We understand your concern about the potential toxicity of the overexpression of LSL-RfxCas13d, which should indeed be carefully considered. A recent preprint study showed that overexpression of RfxCas13d resulted in death in mice after birth, and resulted in more rapid death together with targeting crRNA, suggesting that both overexpression of RfxCas13d and its collateral activity are toxic *in vivo*(5). But it is worth noting that they inserted CAG-RfxCas13d cassette into the mouse genome using the PiggyBac transposon system, resulting in over 50 copies of transgenes per cell(5). Whereas we inserted CAG-LSL-RfxCas13d into the mouse genome by homologous recombination using CRISPR/Cas9 technology, resulting in only two copies of transgenes per cell. The expression levels of RfxCas13d vary widely between our mice and theirs, and the expression of RfxCas13d in our mice is only released in cells in the presence of Cre recombinase. We agree that overexpression of RfxCas13d at the extremely high level could lead to toxicity *in vivo*, but our experimental results suggested that the RfxCas13d-mediated mouse death we observed was dominantly caused by the collateral activity of RfxCas13d rather than the expression of RfxCas13d. The reasons are as follows:

# ①. We obtained the mouse line constitutively expressing RfxCas13d by mating ^LSL^RfxCas13d^fl/fl^ mice with the β-actin-Cre transgenic mice (FVB/N-*Tmem163*^Tg(ACTB-cre)2Mrt^/J; Strain #:003376; This mouse line express Cre recombinase directed by the human beta actin gene promoter). And these mice behaved as normally as WT mice.

# ②. In Fig. 1-3, we retro-orbitally injected ^LSL^RfxCas13d or ^LSL^RfxCas13d^+/fl^Ai14^+/fl^ mice with AAV-PHP.eB carrying hSYN-driven Cre and U6-driven crRNA, targeting crRNAs as the experimental groups, NT crRNA as the control group. In the control group, the expression of RfxCas13d was also released in neurons by hSYN-driven Cre recombinase, but no death of mice was observed. Furthermore, in Fig. 3 (after revision), we demonstrated that expression of any two of RfxCas13d, tdTomato and tdTomato crRNA 7 was not sufficient to cause death in mice, and only simultaneous expression of all three caused death in mice. These data suggested that mice may die from the collateral activity instead of the expression of RfxCas13d.

# ③. We measured the expression level of RfxCas13d in mouse brain and found that it was too low to be directly detected and the extremely weak band of RfxCas13d-HA could only be detected through enrichment using anti-HA magnetic beads (Response Fig. 1b). In addition, a recent study delivered CAG-driven RfxCas13d and U6-driven crRNA using AAV1/9 into spinal cord astrocytes or the striatum for knocking down target genes, and no mouse death caused by overexpression of RfxCas13d was observed(14). Theoretically, the expression level of RfxCas13d in ^LSL^RfxCas13d^fl/fl^ or ^LSL^RfxCas13d^+/fl^Ai14^+/fl^ mice injected with AAV-PHP.eB carrying hSYN-Cre in this study was lower than that in WT mice injected with AAV1/9 carrying CAG-RfxCas13d in the recent study(14), since each AAV-PHP.eB-infected cell had only one or two copies of CAG-RfxCas13d cassette in ^LSL^RfxCas13d^fl/fl^ (two copies) or ^LSL^RfxCas13d^+/fl^Ai14^+/fl^ (one copy) mice, while each AAV1/9-infected cell in WT mice had greater than or equal to one copy of CAG-RfxCas13d cassette. Therefore, RfxCas13d-mediated mouse death we observed was not due to the expression of LSL-RfxCas13d. We think that the reason why the death of mice was observed in our study but not in others is that the AAV subtype and injection method we used determines that RfxCas13d would be expressed more widely in the central nervous system in our study than in previous studies(14, 15), therefore more neurons were affected by the collateral activity of RfxCas13d, resulting in mouse death.

④. Following your suggestions, we packaged AAV-PHP.eB carrying CMV-driven RfxCas13d and U6-driven Sik3-S crRNA 8 or NT crRNA to inject WT mice. As shown below, mice with Sik-S crRNA 8 began to lose body weight at 10 dpi compared with mice with NT crRNA, demonstrating that the *in vivo* toxicity also observed when RfxCas13d was delivered into WT mice via AAV-PHP.eB (Response Fig. 6a).

**Response Figure 6**. **a.** Body weight change curve of WT mice after injection of AAV-PHP.eB carrying CMV-driven RfxCas13d and U6-driven Sik3-S crRNA 8 (n=3) or NT crRNA (n=3). Two-tail unpaired t test was used to compare body weight change between these two groups at indicated dpi. Significance levels are noted as *P < 0.05, **P < 0.01 or ns (P > 0.05). All values are presented as mean ± SEM.

6. Comparing the western blots from 1G to 2A, the knockdown with RfxCas13d/crRNA-8 is not nearly as strong as with Cre, which was also delivered by AAV-PHP.eB, which begs the question, was RfxCas13d engaging with another target potentially? Or was collateral targeting affecting the total amount of protein in the tissue, causing a massive underestimation of knockdown? Did the authors observe any differences in total RNA or total protein between the RfxCas13d-injected mice and the negative control mice?

**Response:** Although both Fig. 1g and Fig. 2a used AAV-PHP.eB to deliver hSYN-driven Cre, they served different purposes. In Fig. 1g, Cre recombinase mediated excision of a tripartite transcriptional stop cassette (STOP) flanked by loxP to release the expression of RfxCas13d. Then, under the guidance of *Sik3-S* crRNAs, RfxCas13d specifically recognized and cleaved *Sik3-S* transcripts to knock down the expression of SIK3-S. In Fig. 2a, Cre recombinase mediated excision of the exon 5 of *Sik3* flanked by loxP to knock out the expression of SIK3 (including SIK3-L and SIK3-S). Generally, knockout can completely block the expression of the target gene, while knockdown can only reduce it, just as the expression of the target gene is still detectable in the knockdown cell line, albeit weakly, but not in the knockout cell line. Therefore, RfxCas13d-mediated knockdown of SIK3-S was not as strong as Cre-mediated knockout.

8. I am confused by the conclusions from the section "RfxCas13d mediated lethality was not caused by off-target effects." When delivering a tdTomato-targeting crRNA, the authors observed toxicity in mice bred to express RfxCas13d and tdTomato, suggesting collateral activity was activated after targeting tdTomato. The fact that no toxicity was observed in mice LSL-RfxCas13d mice injected with the tdTomato-targeting crRNA is expected, because there was no target to activate collateral cleavage. This experiment does not disprove the role of RfxCas13d-mediated off-target effects and in fact supports that toxicity emerges only in the presence of the target transcript, which is still due to RfxCas13d and its "off-target effects."

**Response:** We apologize for the confusion caused by the lack of specific explanations. In our opinion, both the collateral activity and off-target effects of RfxCas13d can cleave untargeted RNAs, but differ in the mechanism. Off-target effects can be defined as unintended cleavage at untargeted DNA/RNA sites showing a similar but not an identical sequence compared to the target site(16, 17). Whereas the collateral activity of Cas13 is triggered by the binding of the target RNA to promiscuously cleave nearby RNAs(18, 19). To make it clear, we have improved this section (Page 9-11 and Fig. 3).

9. When characterizing the role that transcript abundance has on collateral targeting in Fig. 4, the authors observe reduced RfxCas13d protein for some crRNA, but notably not all despite strong knockdown for all targets, indicating a crRNA dependence for this effect. I would appreciate if the authors could speculate on this. In particular, I wonder if the toxicity they observe in vivo can be attenuated by thorough crRNA screening, begging the question on what the result would be if they screened for Sik3-S crRNAs that did not reduce RfxCas13d expression.

**Response:** We speculate that this phenomenon may be due to the following two reasons:

①. Different crRNAs can guide RfxCas13d proteins to bind to different regions of the target gene transcript. RNA binding proteins (RBPs) in different regions may affect the accessibility of RfxCas13d mRNA to the RNase active site on RfxCas13d proteins. Consequently, different crRNAs caused different degrees of reduction in the expression level of RfxCas13d.

②. Besides, different spacer sequences of crRNAs may affect the stability of the activated RfxCas13d-crRNA-target RNA complex, which in turn leads to different strengths of collateral activity.

In our opinion, the *in vivo* toxicity may be attenuated by crRNA screening if the effects of the collateral activity trigged by each crRNA on endogenous RNAs can be fully understood. The protein or mRNA level of RfxCas13d can be used to indicate the collateral activity of RfxCas13d, but is not appropriate (insufficient at least) to indicate its effects on endogenous RNAs. And we believe that the collateral activity of RfxCas13d could cleave RNAs other than 28s rRNA. Therefore, it is necessary to develop a highly-sensitive high-throughput sequencing technology to study all the cleavage substrates and sites of the collateral activity, which will help screen out crRNAs with high knockdown efficiency and little impact on endogenous RNAs, as well as the optimization of Cas13. In addition, different tissues or organs are of different importance to animal viability and may have different degrees of tolerance to collateral activity, which should be carefully considered when using CRISPR/Cas13 *in vivo*.

10. The authors measured collateral targeting in 293 cells as detected by decreases in RfxCas13d protein, but how does this translate across the entire transcriptome? RNA-seq measurements would be very useful for this study.

**Response:** Thanks for your suggestions. Indeed, we performed RNA-seq analysis to study the effects of the collateral activity of RfxCas13d on endogenous RNAs in Fig. 5. In Fig. 4, we showed that the collateral activity of RfxCas13d is positively correlated with the abundance of target genes. And previous studies reported that no collateral activity of Cas13 was detected by RNA-seq upon targeting lowly expressed endogenous genes(1-4). Therefore, to improve the collateral activity of RfxCas13d to investigate its effects on endogenous RNAs, we performed the experiments in Fig. S5a. Through RNA-seq analysis, we obtained 509 common DEGs from four sets of comparisons (Fig. 5e). Interestingly, these were all up-regulated genes compared with NT crRNA (Supplementary Table 1/Table S2 of the final manuscript). These DEGs were thought to be caused by the collateral activity of RfxCas13d as they were independent of the sequence of crRNAs and target genes. Among these genes, we noticed that multiple genes with obvious difference belong to immediate early genes (IEGs) (Fig. 5f). And EGR1 and FOS are the most two significantly different genes in these 509 DEGs (Supplementary Table 1/Table S2 of the final manuscript). Therefore, we first paid attention to these IEGs and found that they were upregulated by ZAKα-JNK/p38-IEG pathway triggered by 28s rRNA breakage. But the reasons for the up-regulation of DEGs other than IEGs are still unclear and further research is needed.

11. I have concerns on the experimental design of the study beginning on Pg. 12. The authors stably express RfxCas13d but transfect the gene target. As the authors show, expression strength can influence the collateral targeting. I am concerned that the overexpression effects from this assay influenced the findings. Were similar effects observed when targeting endogenously expressed transcripts (low, medium and high thresholds).

**Response:** We understand your concerns. Indeed, we observed the breakage of 28s rRNA as well when targeting endogenously highly-expressed genes (Fig. S5c). Furthermore, we measured and found that the expression levels of EGR1 and FOS were upregulated upon targeting endogenously highly-expressed genes (Response Fig. 7a-b). These data demonstrated that the collateral activity of RfxCas13d also caused the breakage of 28s rRNA and activation of EGR1 and FOS upon targeting endogenously highly-expressed genes.

In addition, we selected two endogenously lowly-expressed genes (B4GALNT1 and ANXA4) as the targets and found that no breakage of 28r RNA and activation of EGR1 and FOS was observed (Response Fig. 7c-h), which is consistent with previous study (The sequences of crRNAs we used are the same as these they used)(3). These results did not imply that RfxCas13d did not exhibit collateral activity when targeting endogenously low-expressed genes, but rather that its collateral activities triggered by lowly-expressed genes were too weak to be detected by the methods we used. The expression levels of these endogenous genes are showed in Response Fig. 7i. (from RNA-seq analysis).

**Response Figure 7**. **a-b.** RT-qPCR to measure the RNA level of EGR1 (**a**) and FOS (**b**) in WT HEK293T cells 24 h after transfection of plasmids encoding RfxCas13d and NT crRNA (n=3) or indicated crRNAs (n=3). **c-e.** WT HEK293T cells were collected for total RNA integrity analysis and RT-qPCR 24 h after transfection of plasmids encoding RfxCas13d and NT crRNA (n=3) or B4GALNT1 crRNAs (n=3). **c.** Quality control of total RNA of each sample was quantified by Agilent 2200 Bioanalyzer. **d-e.** RT-qPCR to measure the RNA level of EGR1 (**a**) and FOS (**b**) in each sample. **f-h.** WT HEK293T cells were collected for total RNA integrity analysis and RT-qPCR 24 h after transfection of plasmids encoding RfxCas13d and NT crRNA (n=3) or ANXA4 crRNAs (n=3). **f.** Quality control of total RNA of each sample was quantified by Agilent 2200 Bioanalyzer. **g-h.** RT-qPCR to measure the RNA level of EGR1 (**a**) and FOS (**b**) in each sample. **i.** The table to show the expression levels (FPKM) of indicated genes in HEK293T cells (data from RNA-seq). **a-b, d-e, g-h,** One-way ANOVA with Dunn’s multiple comparisons test. Significance levels are noted as **P < 0.01, ****P < 0.0001 or ns (P > 0.05). All values are presented as mean ± SEM.

12. Did the observe rRNA decreases in their in vivo experiments? There is currently no evidence suggesting a role for ZAKalpha or 28s rRNA for the in vivo toxicity. What is the mechanism for toxicity then?

**Response:** We agree. In this study, we found that knocking down gene expression using RfxCas13d in the adult mouse brain neurons caused death of mice and proved that RfxCas13d-mediated lethality might be caused by the collateral activity of RfxCas13d instead of the loss of target gene function or off-target effects. Furthermore, we showed that RfxCas13d exhibited collateral activity in mammalian cells, which is positively correlated with the abundance of the target RNA. The collateral activity of RfxCas13d cleaved 28s rRNA into two fragments, leading to translation attenuation and activation of the ZAKα-JNK/p38-IEG pathway. However, since it is extremely difficult to dissociate adult mouse brain tissue and obtain intact neuron cells, we did not obtain direct evidence suggesting a role for 28s rRNA breakage or activation of ZAKα-JNK/p38-IEG pathway for the *in vivo* toxicity. Therefore, we have decided to change the title to “The collateral activity of RfxCas13d causes death in mice”. We believe that the collateral activity of RfxCas13d also cleaved RNAs other than 28s rRNA, although we have not yet identified them. Consequently, the death of mice may be due to the collateral cleavage of multiple RNAs by RfxCas13d. Considering the high complexity *in vivo*, we are trying to develop a highly-sensitive high-throughput sequencing method to first detect all cleavage substrates and sites in cell lines, and then investigate the *in vivo* toxicity mechanism of collateral activity.

**Minor comments:**

1. The authors cite Zhuo et al. Cell 2020;181(3):590-603 e16. However, it should be noted that this has emerged as very controversial work. Beyond now several groups not being able to replicate its central reprogramming findings, one group was specifically unable to replicate its knockdown findings in vivo.

**Response:** Thanks for your information and we also believe that the conclusions of this literature are indeed controversial. We cited this literature only to demonstrate the generality and safety issue of RfxCas13d’s application, not to support its conclusions. Considering its controversial nature, we have decided not to cite this literature.

2. For additional evidence on the ability for RfxCas13d to target transcripts in the CNS, the authors should cite Powell et al. Sci Adv 2022 8, eabk2485 (2022). No severe adverse in vivo effects were observed in this work, though the authors did report that RfxCas13d silenced more non-target transcripts than previously suggested from the literature.

**Response:** Thanks for your suggestions and we have cited this literature in multiple places.

3. The authors should discuss the results by the Wilusz laboratory, which also observed that Cas13 collateral targeting may be due to transcript threshold (Nucleic Acids Res. 2022: https://doi.org/10.1093/nar/gkac159).

**Response:** We are glad that they came to the same conclusions as ours. They also found that the collateral activity of Cas13 is positively correlated with the abundance of target genes in mammalian cells, which is consistent with our study and multiple recent preprint studies(5-8). We discussed these results in Page 21-22 lines 457-468.

**Response to Reviewer #2:**

**Major points:**

-	The authors need to cite during all the manuscript the literature related with their findings. This reviewer can understand that preprints could not be cited (although I would strongly recommend it, especially when they support their results) but the recent paper published in NAR (PMID: 35244715) should be acknowledged and cited in all the statements regarding the collateral activity in mammalian cell culture including the findings about the more expression of the target the clearer the collateral. For example, in page 11: These data indicated that the collateral activity of RfxCas13d was triggered and positively correlated with the abundance of target RNA in mammalian cells, when targeting exogenous genes. There are multiples examples where this reference should be included. Thus, a complete revision of the manuscript is needed.

**Response:** Thanks for your suggestions. We have revised the manuscript and cited this recently published study and multiple preprint studies (supporting our findings) in multiple places.

-	Figure 2 g-i. It is great to see the transcriptomic data in these experiments but this reviewer wonders whether the collateral activity is detected in these experiments. Have the authors checked this? Similarly, authors showed RNAseq of Rbfox3 in cells with very low expression. Could it be more informative to show RNAseq data associated to panel 2f (western blot). And, is the collateral activity detected in Fig 2f conditions?

**Response:** We checked and found approximately 150-600 differentially expressed genes (DEGs) per targeting crRNA compared to NT crRNA (Response Table 2/Table S1 of the final manuscript). These DEGs may resulted from target genes loss of function, collateral activities, off-target effects or other reasons. Because of the low expression levels of these target genes, they only triggered weak collateral activities (Response Fig. 1a). And previous studies reported that no collateral activity of Cas13 was detected by RNA-seq upon targeting lowly-expressed endogenous genes(1-4). Moreover, a recent published study and several preprint studies showed that the collateral activity of RfxCas13d was positively corrected with the RNA abundance of target genes, and the collateral activities were too weak to be detected upon targeting lowly-expressed genes(5-8). Therefore, although we observed untargeted DEGs, we could not directly conclude which DEGs were attributable to the collateral activities.

As you suggested, we performed RNA-seq analysis (Using Rbfox3 crRNA 3 and 5) associated to Fig. 2f. Firstly, total RNA integrity analysis showed that 28s rRNA breakage was observed in cells transfected with Rbfox3 crRNA 3/5 but not NT crRNA (Response Fig. 8a). And then RNA-seq analysis (Response Table 3/Table S8 of the final manuscript) showed that multiple IEGs were upregulated in Rbfox3 crRNA 3/5 groups compared to NT crRNA group (Response Fig. 8b). Furthermore, we measured the expression level of RfxCas13d in Fig. 2f and found that it also decreased when the expression level of NeuN was knocked down (Response Fig. 8c). These data demonstrated that RfxCas13d also exhibited collateral activity upon targeting NeuN, resulting in 28s rRNA breakage and activation of IEGs.

**Response Figure 8**. **a-b.** HEK293T cells were collected for total RNA integrity analysis and RNA-seq analysis 24 h after transfection of plasmids encoding RfxCas13d, NeuN and Rbfox3 crRNAs or NT crRNA. **a.** Quality control of total RNA of samples by Agilent 2200 Bioanalyzer. **b.** Heatmap representing the log2 fold-change values of IEGs in targeting crRNA-transfected groups vs. NT crRNA-transfected group. **c.** Western blot to measure the protein level of RfxCas13d, NeuN and ACTB in HEK293T cells 24 h after transfection of plasmids encoding RfxCas13d, NeuN and Rbfox3 crRNAs or NT crRNA.

-	Then, in the in vivo experiments (fig 2 j-l) it came to my attention that only 1 out the 2 gRNAs used triggers toxicity. Can the authors explain whether this can be due to the use of different gRNAs or little but still significant differences in RNA depletion can explain this. Is there a minimal threshold of the depletion to induce toxicity? For example, it is very intriguing in Fig 3a-d to see how with very similar level of depletion (by qPCR and RNA-seq) of 2 independent gRNAs one triggers toxicity in vivo and the other not. Authors should discuss these results.

**Response:** In our experience, the lethal crRNAs were only slightly more efficient for knockdown than the non-lethal crRNAs in HEK293T or N2a cells. However, the expression levels of target genes were significantly decreased in the lethal crRNA groups, but not in the non-lethal crRNA groups *in vivo*. To determine whether the non-lethal crRNAs were low-efficient or completely ineffective, we measured the expression level of tdTomato in surviving mice from tdTomato crRNA 4 and NT crRNA groups at 74 dpi before, and found that the expression level of tdTomato was significantly reduced in tdTomato crRNA 4 group, compared to NT crRNA group (Response Fig. 9a) (The expression level of tdTomato was measured at 12 dpi in Fig. 3e). This suggested that tdTomato crRNA 4 can also mediate the knockdown of tdTomato *in vivo*, but not as efficiently as tdTomato crRNA 7. Hence, we think that both crRNAs of each target gene can mediate the knockdown of the target gene *in vivo*, but differ in the efficiency. The lethal crRNAs mediated high knockdown efficiency and elicited strong collateral activities, which exceeded the tolerance threshold of mice, resulting in mouse death. Whereas, the non-lethal crRNAs mediated low knockdown efficiency and elicited weak collateral activities below the tolerance threshold of mice, so no mouse death was observed. These discussions have been added on Page 21.

**Response Figure 9**. **a.** Western blot to measure the protein level of tdTomato in the brains from ^LSL^RfxCas13d^fl/fl^ mice 74 days after injection of AAV-PHP.eB carrying hSYN-driven Cre and U6-driven indicated crRNAs. NT: NT crRNA; td-cr4: tdTomato crRNA 4.

-	Fig S4b is confusing. This is aiming to show that LwaCas13a induces collateral activity. However, it only shows that LwaCas13a reduces GFP mRNA as fast as 4h post transfection of the in vitro synthesized crRNA and according to ribosomal data, no evidence of collateral activity is observed. Could it be that to trigger collateral activity a sustained expression of Cas13 and gRNA is needed? Indeed, the proliferation defects showed here and in other reports are clearly more evident at 48 h post transfection suggesting that an accumulation of the hyperactivity is needed to be physiologically relevant. Since the author have RNPs they could use them to test whether with the opposite kinetic of plasmid transfection the collateral effect is observed (high targeting after first hours post transfection and then degradation of the RNP vs higher activity along the accumulation of Cas13d and crRNA from plasmid expression).

**Response:** Initially, our animal experiments (Fig. 1-3) suggested that the death of mice might be caused by the collateral activity of RfxCas13d. Therefore, we reviewed previous studies about the collateral activity of Cas13 and found that one study reported that LwaCas13a exhibited significant collateral activity in U87 cells and resulted in cell death(20). To validate their findings, we transfected *in vitro*-synthesized EGFP crRNA or NT crRNA into U87 cells stably expressing LwaCas13a and EGFP as described by them. The protein sequence of LwaCas13a and the sequence of crRNAs we used were identical to theirs. RT-qPCR results showed that EGFP mRNA was significantly knocked down at 4 and 8 hours after transfection with EGFP crRNA compared to NT crRNA (Response Fig. 10a), which is consistent with their results. However, RNA denaturing gel electrophoresis of total RNA showed that 28s and 18s rRNAs were intact, and not cleaved into multiple bands as they reported (Response Fig. 10a). Although we later demonstrated in Fig. S4c (after revision) that LwaCas13a did exhibit collateral activity in HEK293T cells. we still have not figured out why we cannot observe the same phenomenon as they observed in U87 cells. Following your suggestions, we co-transfected plasmids encoding LwaCas13a, EGFP and crRNA into 293T cells and harvested cells 48 hours after transfection for RT-qPCR and total RNA integrity analysis. As shown below, LwaCas13a exhibited collateral activity and cleaved rRNAs, but the cleavage pattern and extent of disruption were different from what they reported (Response Fig. 10c-d). Given the controversial nature of these results, we have decided to remove this section from the manuscript. And we totally agree with your opinion about the opposite kinetic between plasmid transfection and RNP transfection.

**Response Figure 10**. **a.** Upper: RT-qPCR to measure the expression level of EGFP in U87 cells stably expressing LwaCas13a and EGFP after transfection of *in-vitro* synthesized NT crRNA (n=3) or EGFP crRNA (n=3) at the indicated time. Bottom: RNA denaturing gel to measure the integrity of total RNA in Upper. **b-d.** RT-qPCR and total RNA integrity analysis were used to measure the expression levels of indicated genes, RNA integrity respectively in HEK293T cells 48 h after transfection of plasmids encoding LwaCas13a, EGFP and NT crRNA or *EGFP* crRNA. **b-c.** RT-qPCR to measure the RNA level of EGFP and LwaCas13a. **d.** Quality control of total RNA of samples by Agilent 2200 Bioanalyzer. **a,** One-way ANOVA with Dunn’s multiple comparisons test. **b-c,** Two-tailed unpaired t-test. Significance levels are noted as ***P < 0.001, ****P < 0.0001 or ns (P > 0.05). All values are presented as mean ± SEM.

-	Fig 4Se-f, can the authors show qPCR or RNAseq associated to this panel? Since the collateral effect is on the RNA, this reviewer would recommend showing RNA targeting when possible.

**Response:** As you suggested, we measured the expression level of tdTomato and LwaCas13a or PspCas13b by qPCR. As shown below (Response Fig. 11a-b/Revised Manuscript Fig. S4c-d), when LwaCas13a and PspCas13b bound and cleaved the target RNA under guidance of targeting crRNAs, their own mRNAs were also significantly reduced, indicating that LwaCas13a and PspCas13b also exhibited collateral activity in mammalian cells.

**Response Figure 11**. **a-b.** RT-qPCR to measure the expression level of tdTomato and LwaCas13a/PspCas13b in HEK293T 24 h after transfection of plasmids encoding LwaCas13a/PspCas13b, tdTomato and NT crRNA (n=3) or crRNAs targeting tdTomato (n=3). td crRNA 1/2: tdTomato crRNA 1/2. **a-b**, One-way ANOVA with Dunn’s multiple comparisons test. Significance levels are noted as **P < 0.01, ***P < 0.001, ****P < 0.0001 or ns (P > 0.05). All values are presented as mean ± SEM.

-	Fig 5. This is clearly the novelty of this manuscript, but it would be more convincing if the authors could demonstrate the molecular mechanism downstream the collateral activity in other mammalian cell line (beyond 293T) at least to see how general this response is.

**Response:** Thanks for your suggestions. We measured RNA integrity, protein synthesis rate and the expression levels of tdTomato, RfxCas13d, EGR1 and FOS in Hela, MDA-MB-231 and HCT116 cells after co-transfection of plasmids encoding RfxCas13d, tdTomato, and NT crRNA or tdTomato crRNA 7. As shown below (Response Fig. 12/Revised Manuscript Fig. S7), compared with cells transfected with NT crRNA, the mRNA level of RfxCas13d was decreased when tdTomato was knocked down in cells transfected with tdTomato crRNA 7, which is accompanied by 28s rRNA breakage(m-o), translation attenuation (p-r) and activation of EGR1 and FOS (g-l). These results demonstrated that the collateral activity of RfxCas13d can also cleave 28s rRNA in these three cell lines, leading to translation attenuation and activation of ZAKα-JNK/p38-IEG pathway.

**Response Figure 12**. RT-qPCR, total RNA integrity analysis and SUnSET were used to measure the expression levels of indicated genes, RNA integrity and protein synthesis respectively in Hela, MDA-MB-231and HCT116 cells 24 h after transfection of plasmids encoding RfxCas13d, tdTomato and NT crRNA or tdTomato crRNA 7. **a-l.** RT-qPCR to measure the mRNA levels of tdTomato (**a-c**), RfxCas13d (**d-f**), EGR1 (**g-i**) and FOS (**j-l**). **m-o.** Quality control of total RNA of samples by Agilent 2200 Bioanalyzer. **p-r.** SUnSET essay to measure the protein translation rate. **a-l,** two-tailed unpaired t-test (n=3), Significance levels are noted as **P < 0.01, ***P < 0.001, ****P < 0.0001 or ns (P > 0.05). All values are presented as mean ± SEM.

-	Statistical analysis should be done. There is only a section in methods where authors claimed to have done t-test to compare 2 groups, but it is not indicated in any figure. Moreover, when comparing more than 2 groups an Anova test is required at least. Figure legends should include this information.

**Response:** Thanks for your suggestions. We have performed statistical analysis and described the statistical tests used for each figure in the corresponding figure legends.

**Minor points:**

-	Methods should be better explained in general. It is not clear, for example, what Cas13 versions are used during the whole manuscript, how GFP crRNA in vitro synthesized is generated (in vitro transcription?), how plasmids were constructed (just mentioning standard molecular biology techniques is not sufficient, especially when the plasmids used in the manuscript are not properly detailed). Please, review the whole section to make the manuscript more transparent.

**Response:** Thanks for your suggestions. We have improved the Methods section to make it more detailed.

-	Discussion: pag 17 (345-47), authors could speculate a bit how to reduce collateral activity. A couple of preprints have been recently deposited about it.

**Response:** Thanks for your suggestions and we have added a speculation of how to reduce collateral activity in the Discussion section (Page 22 Line 468-472).

-	Some statements are too overweight. For example, in results: These results demonstrated 275 that RNA structure and RBPs were involved in protecting RNA. Suggest looks more accurate. Or This explained why in Fig. 4a, changes in protein levels of RfxCas13d were more obvious than changes in RNA levels. This may/could explain would fit better.

**Response:** Thanks for your suggestions and we have made changes.

-	Authors stated: transfection of plasmids encoding LwaCas13a, PspCas13b or RfxCas13d into U87 cells, regardless of with targeting or NT crRNA, would affect cell viability, indicating that cell viability changes have nothing to do with collateral activity. I do not see this in the paper authors cited. For example, Fig 4e of this paper (Ozcan et al., 2021) shows that with NT crRNA no toxicity is observed using Cas13d. Please review this properly and change or remove the statement.

**Response:** Thanks for your suggestions and we have removed the statement.

-	Page 6 lines line 122 there is a wrong figure called.

**Response:** Thanks for the reminder and we have revised it.

-	Page 3 lines 59: Surprisingly, binding of the target RNA to Cas13-crRNA complex also activates a nonspecific RNase activity, which promiscuously cleaves bystander RNAs without… As far as this reviewer knows the collateral effect needs RNA cut to be triggered and not only Cas13 binding to the RNA. Please correct the sentence accordingly.

**Response:** Thanks for your suggestions and we have revised this sentence (Page 3-4 lines 61-68).

**Reference:**

1.	Cox DBT, Gootenberg JS, Abudayyeh OO, Franklin B, Kellner MJ, Joung J, et al. RNA editing with CRISPR-Cas13. Science. 2017;358(6366):1019-27.

2.	Xu C, Zhou Y, Xiao Q, He B, Geng G, Wang Z, et al. Programmable RNA editing with compact CRISPR-Cas13 systems from uncultivated microbes. Nat Methods. 2021;18(5):499-506.

3.	Konermann S, Lotfy P, Brideau NJ, Oki J, Shokhirev MN, Hsu PD. Transcriptome Engineering with RNA-Targeting Type VI-D CRISPR Effectors. Cell. 2018;173(3):665-76 e14.

4.	Abudayyeh OO, Gootenberg JS, Essletzbichler P, Han S, Joung J, Belanto JJ, et al. RNA targeting with CRISPR-Cas13. Nature. 2017;550(7675):280-4.

5.	Tong H, Huang J, Xiao Q, He B, Dong X, Liu Y, et al. High-fidelity Cas13 variants for targeted RNA degradation with minimal collateral effect. bioRxiv. 2021.

6.	Kelley CP, Haerle MC, Wang ET. Negative autoregulation mitigates collateral RNase activity of repeat-targeting CRISPR-Cas13d in mammalian cells. bioRxiv. 2021.

7.	Ai Y, Liang D, Wilusz JE. CRISPR/Cas13 effectors have differing extents of off-target effects that limit their utility in eukaryotic cells. Nucleic Acids Res. 2022;50(11):e65.

8.	Shi P, Murphy MR, Aparicio AO, Kesner JS, Fang Z, Chen Z, et al. RNA-guided cell targeting with CRISPR/RfxCas13d collateral activity in human cells. bioRxiv. 2021.

9.	Funato H, Miyoshi C, Fujiyama T, Kanda T, Sato M, Wang Z, et al. Forward-genetics analysis of sleep in randomly mutagenized mice. Nature. 2016;539(7629):378-83.

10.	Hohenstein P, Slight J, Ozdemir DD, Burn SF, Berry R, Hastie ND. High-efficiency Rosa26 knock-in vector construction for Cre-regulated overexpression and RNAi. Pathogenetics. 2008;1(1):3.

11.	Chu VT, Weber T, Graf R, Sommermann T, Petsch K, Sack U, et al. Efficient generation of Rosa26 knock-in mice using CRISPR/Cas9 in C57BL/6 zygotes. BMC Biotechnol. 2016;16:4.

12.	Wang G, Li Q, Xu J, Zhao S, Zhou R, Chen Z, et al. Somatic genetics analysis of sleep in adult mice. J Neurosci. 2022.

13.	Chan KY, Jang MJ, Yoo BB, Greenbaum A, Ravi N, Wu WL, et al. Engineered AAVs for efficient noninvasive gene delivery to the central and peripheral nervous systems. Nat Neurosci. 2017;20(8):1172-9.

14.	Powell JE, Lim CKW, Krishnan R, McCallister TX, Saporito-Magrina C, Zeballos MA, et al. Targeted gene silencing in the nervous system with CRISPR-Cas13. Sci Adv. 2022;8(3):eabk2485.

15.	Zhou H, Su J, Hu X, Zhou C, Li H, Chen Z, et al. Glia-to-Neuron Conversion by CRISPR-CasRx Alleviates Symptoms of Neurological Disease in Mice. Cell. 2020;181(3):590-603 e16.

16.	Modrzejewski D, Hartung F, Lehnert H, Sprink T, Kohl C, Keilwagen J, et al. Which Factors Affect the Occurrence of Off-Target Effects Caused by the Use of CRISPR/Cas: A Systematic Review in Plants. Front Plant Sci. 2020;11:574959.

17.	Manghwar H, Li B, Ding X, Hussain A, Lindsey K, Zhang X, et al. CRISPR/Cas Systems in Genome Editing: Methodologies and Tools for sgRNA Design, Off-Target Evaluation, and Strategies to Mitigate Off-Target Effects. Adv Sci (Weinh). 2020;7(6):1902312.

18.	Zhang C, Konermann S, Brideau NJ, Lotfy P, Wu X, Novick SJ, et al. Structural Basis for the RNA-Guided Ribonuclease Activity of CRISPR-Cas13d. Cell. 2018;175(1):212-23 e17.

19.	Slaymaker IM, Mesa P, Kellner MJ, Kannan S, Brignole E, Koob J, et al. High-Resolution Structure of Cas13b and Biochemical Characterization of RNA Targeting and Cleavage. Cell Rep. 2019;26(13):3741-51 e5.

20.	Wang Q, Liu X, Zhou J, Yang C, Wang G, Tan Y, et al. The CRISPR-Cas13a Gene-Editing System Induces Collateral Cleavage of RNA in Glioma Cells. Adv Sci (Weinh). 2019;6(20):1901299.

**Second round of review**

**Reviewer reports:**

**Reviewer #2:** The authors have conveniently answered to some of my points and the new data using different cell lines is appreciated, but this reviewer has still some concerns and there are several important points to be addressed:

1)	Fig 4c, in the inducible experiment. While lack of Cas13d is greater when higher level of doxycycline is used, this does not correlate at all with lower level of tomato (the target of the crRNAs). One possibility is that tomato protein is very stable, and the depletion of the mRNA is not translated in lower protein in these conditions. To see this, a qPCR test should be done. If the ultimate goal is to demonstrate that increased levels of mRNA triggers more collateral activity, tomato mRNA downregulation should be shown. Moreover, this could be the problem with the lack of correlation observed between gRNA efficiency and collateral activity (see next point)

2)	The authors argued that the lack of collateral activity for some gRNAs targeting highly abundant mRNAs is due to lower efficiency, but this is not really correlating well in all experiments. For example, in Figure 4d-I, there are gRNAs inducing similar levels of depletion of the same mRNA that in some cases reduce Cas13d protein and in other cases not. And it is particularly relevant crRNA 3 ywhae where with then lowest depletion the lowest Cas13d are observed. My advice is again to measure mRNA levels by qPCR or RNAseq and if possible, use other markers of collateral activity different from Cas13d levels (which is the trigger of the collateral activity). Bioanalyzer data that shows the extra band from 28S fragmentation

could help to show whether there is a clear collateral in these experiments.

3)	Indeed, 28S fragmentation looks like a very good marker for the collateral activity. Authors argue that RfxCas13d targeting low abundant mRNAs can even trigger collateral although current technologies can not detect any sign of that. Since CRISPR-RfxCas13d targeting low-medium abundant mRNAs do no trigger significant toxicity and there is no any molecular marker that can detect this unregulated RNAse activity from Cas13d, this reviewer would strongly suggest to rephrase the speculation about this in the manuscript.

4)	From the rebuttal letter, authors stated:

"We checked and found approximately 150-600 differentially expressed genes (DEGs) per targeting crRNA compared to NT crRNA (Response Table 2). These DEGs may resulted from target genes loss of function, collateral activities, off-target effects or other reasons…. Therefore, although we observed untargeted DEGs, we could not directly conclude which DEGs were attributable to the collateral activities." This reviewer is not convinced about the consistency of this data. A good control to see whether these differentially expressed genes (what is the cut-off and adjusted pValue to call them DEG?) are somehow related with RfxCas13d activity is to perform some RNAseq using dCas13d and comparing dCas13d with NT crRNA vs dCas13d T crRNA (Targets from this statment) and see what is the background of gene expression in these conditions where no collateral and no off-targets and no loss-of-function occurs.

5)	Authors have used RNP in their in vitro experiments, but the source of the protein is missing in their manuscript. Is commercially available? Is it purified in the lab? If this last is the case, authors should describe in methods the purification protocol. In this regard, this reviewer suggested to test whether opposite kinetics of RNP (fast and short-term targeting) vs plasmids (sustained targeting) could explained whether the accumulation of the reagents (Cas13 and gRNAs) with time could increase the collateral effects. However, the authors have not performed any experimental approach to test this hypothesis. This reviewer strongly encourages the authors to show some data on this. This will further characterize and add new data on the collateral effects in mammalian cells which is one of the main goals of the manuscript

6)	The new discussion is too long and sometimes too repetitive (repeating same concept or something described in results section) and speculative. For example, "We think it would be useful if a "cap" domain could be designed and fused to Cas13 to cover its RNase active site, thereby reducing the accessibility of bystander RNAs to the RNase site without affecting Cas13 binding and cleavage of target RNAs". Rather than this speculation, the authors have the possibility here to comment on the recent published papers and preprints where solutions to the collateral activity of RfxCas13d have been proposed (PMID: 35953673, PMID: 35977479, https://www.biorxiv.org/content/10.1101/2021.09.14.460134v2)

Please review all the discussion and compare it with what is described in results to avoid unnecessary redundant information

**Reviewer #1:** I appreciate the authors detailed responses to my comments and the new data to address my previous concerns. The majority of their points were clear and resolved outstanding concerns, however, their disclosure related to their inability to detect RfxCas13d in the brain concerns me and potentially casts doubt on the methods behind the manuscript’s central claim.

1. The authors are using a CAG-loxP-STOP-loxP cassette to express RfxCas13d conditionally. This is unquestionably a strong promoter that, when used to control Cas9 expression, should lead to its unambitious detection (https://doi.org/10.1016/j.cell.2014.09.014 among many studies).

It is concerning to me that, for a study claiming that in vivo collateral targeting in the brain leads to lethality, the authors do not show convincing data confirmed RfxCas13d expression in the brain (it is beyond odd to me that a magnetic bead pulldown assay is required to detect the protein from lysate). Further, the authors show widespread Cre expression throughout the brain, which one would expect would translate with widespread RfxCas13d expression, even if restricted to neurons. This is concerning because, without stronger data demonstrating RfxCas13d expression, it is conceivable the lethality could be resulting from a different mechanism. Perhaps a RfxCas13d variant?

The bottom line is the authors unequivocally must demonstrate that RfxCas13d is expressed from their engineered mouse line. Not just for their conclusions, but also for the point of thoroughly characterizing their new mouse line.

2. Related to the new data on AAV-delivered RfxCas13d (shown in Response Figure 6). Numerous important details are missing that key for interpreting the data. One, how old were the animals when injected? It is odd that the non-targeted group is, over the course of the 20 days, not losing or not gaining weight. Two, the study should be balanced with an equal number of mice, preferably equal numbers of males and females. As males weigh more than females, this can heavily skew a weight analysis.

3. On Pg. 21, the authors suggest several potential mechanisms about why they, but not other groups, observed lethality. I believe there speculative list is incomplete. Other potential explanations could be: differences in target transcript threshold that could affect the extent of collateral targeting (analogous to the in vitro effect); differences in the strength of RfxCas13d expression from differences in promoter strength, differences in copy number per cell due to differences in vector dosing or serotype and finally the relative ratio of Cas13 to target transcript, which can be affected by delivery.

4, Since originally reviewed, a high-fidelity version of RfxCas13d has been reported in the literature. This variant should be highlighted in the Discussion.

Reviewer #2: The authors have conveniently answered to some of my points and the new data using different cell lines is appreciated, but this reviewer has still some concerns and there are several important points to be addressed:

--------------------

**Evaluation.** Has the author satisfactorily responded to your previous review?

**Reviewer #1:** No

**Reviewer #2:** No

**Author response:**

Dear Dr. Pang

We apologize for not fully addressing all the concerns raised by the reviewers during the first revision. Thank you for giving us another opportunity to revise our manuscript “The collateral activity of RfxCas13d causes death of mice” (GBIO-D-22-00216R1). We deeply appreciate the time and effort that you and the reviewers dedicated to providing feedback on our manuscript and are very grateful for the insightful comments on and valuable improvements to our paper. We have performed experiments and revised the manuscript to address the concerns of the reviewers. Those changes to the manuscript are indicated in the manuscript using track changes. The point-by-point responses for the concerns of the reviewers are attached below. All page and line numbers refer to the revised manuscript file.

**Response to Reviewer #2:**
Reviewer #2: The authors have conveniently answered to some of my points and the new data using different cell lines is appreciated, but this reviewer has still some concerns and there are several important points to be addressed:

1) Fig 4c, in the inducible experiment. While lack of Cas13d is greater when higher level of doxycycline is used, this does not correlate at all with lower level of tomato (the target of the crRNAs). One possibility is that tomato protein is very stable, and the depletion of the mRNA is not translated in lower protein in these conditions. To see this, a qPCR test should be done. If the ultimate goal is to demonstrate that increased levels of mRNA triggers more collateral activity, tomato mRNA downregulation should be shown. Moreover, this could be the problem with the lack of correlation observed between gRNA efficiency and collateral activity (see next point).

**Response:** We agree. Since the cells were pretreated with doxycycline to induce the expression of tdTomato, it is possible that the reduction of tdTomato at mRNA level was not reflected at protein level due to its high stability(1). As suggested by the reviewer, we measured the mRNA levels of RfxCas13d and tdTomato by RT-qPCR under the same experimental condition. The mRNA level of tdTomato increased after treatment with doxycycline in a dose dependent manner (Response Fig. 1a/Revised Manuscript Fig. 4c). Compared with NT crRNA, the mRNA level of tdTomato was significantly knocked down by RfxCas13d with targeting crRNAs, and the mRNA level of RfxCas13d was also down-regulated at the same time (Response Fig. 1a-b/Revised Manuscript Fig. 4c-d). RfxCas13d was negatively correlated with tdTomato at the expression level under co-transfection of RfxCas13d with targeting crRNAs instead of NT crRNA (Response Fig. 1a-b). These data indicated that the collateral activity of RfxCas13d was positively correlated with the abundance of target RNA in mammalian cells. Besides, tdTomato crRNA 7 exhibited slightly higher knockdown efficiency and collateral activity than tdTomato crRNA 4.

**Response Figure 1.** **a-b.** RT-qPCR to measure the mRNA levels of tdTomato (**a**) and RfxCas13d (**b**) 24 hours after transfection of the plasmids respectively encoding RfxCas13d and crRNA into the inducible-expressing tdTomato HEK293T cells. The cells were pretreated with different concentrations of doxycycline for 16 hours. td crRNA 4/7: tdTomato crRNA 4/7. **a-b,** One-way ANOVA with Dunn’s multiple comparisons test. Significance levels are noted as ***P < 0.001, ****P < 0.0001 or ns (P > 0.05). All values are presented as mean ± SEM.

2) The authors argued that the lack of collateral activity for some gRNAs targeting highly abundant mRNAs is due to lower efficiency, but this is not really correlating well in all experiments. For example, in Figure 4d-I, there are gRNAs inducing similar levels of depletion of the same mRNA that in some cases reduce Cas13d protein and in other cases not. And it is particularly relevant crRNA 3 ywhae where with then lowest depletion the lowest Cas13d are observed. My advice is again to measure mRNA levels by qPCR or RNAseq and if possible, use other markers of collateral activity different from Cas13d levels (which is the trigger of the collateral activity). Bioanalyzer data that shows the extra band from 28S fragmentation could help to show whether there is a clear collateral in these experiments.

**Response:** Thanks for your suggestions. To measure the collateral activity triggered by each crRNA targeting endogenous genes, we co-transfected the plasmids respectively encoding RfxCas13d, crRNA and mCherry into HEK293T cells, and measured the mRNA levels of RfxCas13d and mCherry by RT-qPCR. Changes in the expression level of fluorescent protein, as another indicator of the collateral activity of RfxCas13d, have been widely used in recent publications(2-6) and are easier to be quantified than 28s rRNA fragmentation. Since RfxCas13d is followed by T2A-EGFP in the plasmid, EGFP could co-express with RfxCas13d. Compared with NT crRNA, the fluorescence intensities of EGFP and mCherry were down-regulated under co-transfection of RfxCas13d with targeting crRNAs (Response Fig. 2a-g). Consistently, the mRNA levels of RfxCas13d and mCherry were also down-regulated (Response Fig. 2h-u/Revised Manuscript Fig. 4h-m and S4e-p). And the mRNA changes of RfxCas13d and mCherry were synchronized (Response Fig. 2h-u). Response Fig. 2v-B (Revised Manuscript Fig. 4e-g and S4e-h) showed the knockdown efficiency of each crRNA for different genes. Comparing with the collateral activities showed by RfxCas13d and mCherry, the crRNAs with high knockdown efficiency did not always cause high collateral activity (Response Fig. 2h-B). Thus, there is indeed a poor correlation between knockdown effects and collateral activities of RfxCas13d upon targeting these endogenous genes.

However, the expression changes of RfxCas13d and the target genes were obviously consistent when we examined crRNA knockdown efficiency by co-transfecting the plasmids encoding the target gene, RfxCas13d and crRNA into HEK293T cells (Response Fig. 3a-c). The lethal crRNAs *in vivo*, such as tdTomato crRNA 7, Sik3-S crRNA 8 and Rbfox3 crRNA 5, exhibited slightly higher knockdown efficiency and collateral activity than the non-lethal crRNAs, such as tdTomato crRNA 4, Sik3-S crRNA 1 and Rbfox3 crRNA 3 (Response Fig. 3a-c). The expression levels of the target genes and RfxCas13d were quantified by gray scale analysis to indicate knockdown effects and collateral activities, respectively, and a significantly positive correlation between them was observed (Response Fig. 3d-f). Consistently, this correlation has also been reported in the recent published paper(3). Kelley et al. speculated that both on-target and collateral RNase activities may be driven primarily by the intracellular *K*_D_ of the Cas13d:gRNA:target ternary complex and thus may be positively correlated, and reported that the on-target and collateral activities are strongly linked upon targeting overexpressed CUG_n_ RNA (Fig. 3C in PMID: 35977479)(3). We noticed that these target genes are all exogenous genes. Taken together, these data indicated that the correlation between collateral activities and knockdown effects of RfxCas13d may differ between targeting exogenous and endogenous genes. We guess that there may be other factors that influence the correlation between knockdown effects and collateral activities of RfxCas13d upon targeting endogenous genes, such as RNA binding proteins (RBPs).

Therefore, our previous speculation about why only 1 out the 2 crRNAs used triggered toxicity *in vivo* may apply only to the crRNAs targeting exogenously expressed tdTomato, but cannot be extended to the crRNAs targeting endogenous genes. To make it clear, we have rephrased this discussion to “In this study, we chosen two crRNAs for each gene to perform *in vivo* knockdown, but only 1 of 2 crRNAs caused death in mice. This phenomenon may be caused by different collateral activities triggered by different crRNAs *in vivo*. The lethal crRNAs may elicit strong collateral activity, which exceeded the tolerance threshold of mice, resulting in mouse death. Whereas, the non-lethal crRNAs may elicit collateral activity below the tolerance threshold of mice, so no mouse death was observed.” (Revised Manuscript page 20 line 421-425).

**Response Figure 2. a-g.** Representative images showing the fluorescence intensities of EGFP and mCherry in the HEK293T cells transfected with plasmids respectively encoding RfxCas13d, mCherry and crRNA. WF means wild field. Scale bar: 100 µm. **h-u.** RT-qPCR to measure the mRNA levels of RfxCas13d (**h-n**) and mCherry (**o-u**) in the HEK293T cells 24 hours after transfection of the plasmids respectively encoding RfxCas13d, mCherry, crRNA (ACTB(**h, o**), LDHB(**i, p**), YWHAE(**j, q**), GAS5(**k, r**), HNRNPAB(**l, s**), NCL(**m, t**) and TPT1(**n, u**)). **v-B.** RT-qPCR to measure the knockdown efficiency of ACTB(**v**), LDHB(**w**), YWHAE(**x**), GAS5(**y**), HNRNPAB(**z**), NCL(**A**) and TPT1(**B**) crRNAs in HEK293T cells (n=3). **h-B,** One-way ANOVA with Dunn’s multiple comparisons test. Significance levels are noted as *P < 0.05, **P < 0.01, ***P < 0.001, ****P < 0.0001 or ns (P > 0.05). All values are presented as mean ± SEM.

**Response Figure 3. a-c.** Western blot to detect the expression levels of RfxCas13d (**a-c**), NeuN (**a**), SIK3-S (**b**), tdTomato (**c**) and ACTB (**a-c**) in HEK293T cells 24 hours after transfection of the plasmids respectively encoding RfxCas13d, target genes and crRNA. **d-f.** Scatter pots showing the correlation between the relative expression levels of RfxCas13d and target genes. The relative expression levels were obtained by gray scale analysis of **a-c** panels and normalization by the values in NT crRNA. Black lines indicate ordinary least squares linear regression.

3) Indeed, 28S fragmentation looks like a very good marker for the collateral activity. Authors argue that RfxCas13d targeting low abundant mRNAs can even trigger collateral although current technologies can not detect any sign of that. Since CRISPR-RfxCas13d targeting low-medium abundant mRNAs do no trigger significant toxicity and there is no any molecular marker that can detect this unregulated RNAse activity from Cas13d, this reviewer would strongly suggest to rephrase the speculation about this in the manuscript.
**Response:** Thanks for your suggestions and we have changed the speculation to “It remains to be further investigated whether RfxCas13d exhibits detectable collateral activity upon targeting lowly-expressed genes.” (Page 21 lines 449-450)

4)From the rebuttal letter, authors stated:
"We checked and found approximately 150-600 differentially expressed genes (DEGs) per targeting crRNA compared to NT crRNA (Response Table 2). These DEGs may resulted from target genes loss of function, collateral activities, off-target effects or other reasons…. Therefore, although we observed untargeted DEGs, we could not directly conclude which DEGs were attributable to the collateral activities." This reviewer is not convinced about the consistency of this data. A good control to see whether these differentially expressed genes (what is the cut-off and adjusted pValue to call them DEG?) are somehow related with RfxCas13d activity is to perform some RNAseq using dCas13d and comparing dCas13d with NT crRNA vs dCas13d T crRNA (Targets from this statment) and see what is the background of gene expression in these conditions where no collateral and no off-targets and no loss-of-function occurs.

**Response:** We think this is an excellent suggestion. We performed RNA-seq using RfxCas13d/dRfxCas13d with or without crRNA in N2A cells and identified differentially expressed genes (DEGs) (P-adj < 0.05 and absolute fold change ≥2) by comparing their transcriptome profiles (Response Fig. 4a). 6 DEGs were found in comparison between dRfxCas13d and RfxCas13d alone, and 194~1179 DEGs were identified in comparisons between dRfxCas13d with crRNA and RfxCas13d with crRNA (Response Fig. 4a and Response Table 1/Table S3 of the final manuscript). This indicated that RfxCas13d alone only had little effect on the transcriptome, whereas RfxCas13d equipped with crRNA did.

Since only the HEPN domains were mutated in dRfxCas13d, these DEGs were associated with the HEPN domains, the executor of on-target, off-target and collateral activity. In our opinion, the on-target and off-target effects of RfxCas13d are spacer sequence-dependent, while the substrate selection by collateral activity, to a certain extent, is spacer sequence-independent. Thus, through analysis, we identified 185 common DEGs from seven sets of comparisons between dRfxCas13d with targeting crRNA and RfxCas13d with targeting crRNA (Group 3-9 in Response Fig. 4a, Response Table 1/Table S2 of the final manuscript). These DEGs were spacer sequence-independent and therefore were thought to be associated with collateral activity.

To investigate the function of these DEGs, we performed GO term enrichment analysis and found that these genes were significantly enriched in nucleosome assembly (cluster 1) and ribosome (cluster 2) (Response Fig. 4b). And these nucleosome and ribosome-related genes were up-regulated in all seven sets of comparisons (Response Fig. 4c). Consistently, Tong et al. recently reported that up-regulated genes induced by RfxCas13d targeting RPL4 and PPIA can be enriched in nucleosome assembly(4). Moreover, multiple histone genes and a few ribosome-related genes were found in the DEGs caused by RfxCas13d in HEK293T and U87 cells(7).

As suggested by the reviewer, we also compared the transcriptomes between dRfxCas13d with NT crRNA and dRfxCas13d with targeting crRNA, and no or few (3 and 17) DEGs were found, indicating that crRNA alone had little effect on the transcriptome (Response Fig. 4d and Response Table 1/Table S2 of the final manuscript).

Taken together, these data indicated that these nucleosome and ribosome-related genes were associated with collateral activity, but how collateral activity affected the expression of these genes remains to be further investigated.

**Response Figure 4. a.** The table to show the RNA-seq samples we did in N2a cells, the group comparisons made, and the number of identified DEGs. n=2. The analysis method is the same as in the manuscript. **b.** GO term enrichment analysis (David online) of g185common DEGs from seven sets of comparisons (Group 3-9). BP, CC and MF represents biological process, cellular component and molecular function respectively. **c.** Heatmap showing the log2 fold-change (log2FC) values of nucleosome and ribosome-related DEGs from seven sets of comparisons in N2a cells (Group 3-9). **d.** The table to show the group comparisons made, and the number of DEGs.

5) Authors have used RNP in their in vitro experiments, but the source of the protein is missing in their manuscript. Is commercially available? Is it purified in the lab? If this last is the case, authors should describe in methods the purification protocol. In this regard, this reviewer suggested to test whether opposite kinetics of RNP (fast and short-term targeting) vs plasmids (sustained targeting) could explained whether the accumulation of the reagents (Cas13 and gRNAs) with time could increase the collateral effects. However, the authors have not performed any experimental approach to test this hypothesis. This reviewer strongly encourages the authors to show some data on this. This will further characterize and add new data on the collateral effects in mammalian cells which is one of the main goals of the manuscript.

**Response:** Thanks for your suggestions. The RfxCas13d protein was purified in our lab, and the purification protocol has been added in the Materials and Methods (Revised Manuscript pages 25-26 lines 533-547). As suggested by the reviewer, we transfected RNP or plasmids encoding RfxCas13d and tdTomato crRNA 7 into the HEK293T cells stably expressing tdTomato and then measured the mRNA level of tdTomato by RT-qPCR and total RNA integrity by bioanalyzer in a time-course manner. For plasmids transfection, downregulation of tdTomato and fragmentation of 28s rRNA were observed 12 hours after transfection and became more pronounced with the full and sustained expression of RfxCas13d and crRNA (Response Fig. 5a-b). For RNP transfection, downregulation of tdTomato and fragmentation of 28s rRNA were observed 6 hours after transfection and then recovered with time, probably due to degradation of RNP (Response Fig. 5c-d). These data suggested that the knockdown effect and collateral activity of RfxCas13d exhibited opposite kinetics between plasmids transfection and RNP transfection. Although RfxCas13d also exhibited collateral activity delivered through RNP, the duration was limited, which is determined by the rate of RNP degradation. Therefore, similar to Cas9(8), delivery of RfxCas13d via RNP may be safer than via plasmid. However, if the target gene needs to be maintained at a low expression level for a long time, the persist expression of Cas13 and crRNA is required.

**Response Figure 5. a, c.** RT-qPCR to measure the RNA level of tdTomato at indicated time points after transfection of RNP (**c**) or plasmids (**a**) encoding RfxCas13d and crRNA into the HEK293T cells stably expressing tdTomato. 2.5 ug RfxCas13d protein and 250 ng crRNA were premixed and transfected into one well of a 24-well plate using lipofectamine CRISPRMAX (CMAX00001, Invitrogen). The plasmids respectively encoding RfxCas13d (300 ng) and crRNA (100 ng) were transfected into one well of a 24-well plate using jetPRIME (114-15, Polyplus). Mock means untransfected control. **b, d.** Quality control of total RNA of the samples from **a,** **c** by Agilent 2200 Bioanalyzer. **a, c.** One-way ANOVA with Dunn’s multiple comparisons test. Significance levels are noted as ***P < 0.001, ****P < 0.0001 or ns (P > 0.05). All values are presented as mean ± SEM.

6) The new discussion is too long and sometimes too repetitive (repeating same concept or something described in results section) and speculative. For example, "We think it would be useful if a "cap" domain could be designed and fused to Cas13 to cover its RNase active site, thereby reducing the accessibility of bystander RNAs to the RNase site without affecting Cas13 binding and cleavage of target RNAs". Rather than this speculation, the authors have the possibility here to comment on the recent published papers and preprints where solutions to the collateral activity of RfxCas13d have been proposed (PMID: 35953673, PMID: 35977479, https://www.biorxiv.org/content/10.1101/2021.09.14.460134v2)
Please review all the discussion and compare it with what is described in results to avoid unnecessary redundant information
**Response:** Thanks for your suggestions and we have improved the Discussion section. We have highlighted these recent published papers and preprints about solutions to the collateral activity of RfxCas13d in Revised Manuscript page 22 lines 465-476.

**Response to Reviewer #1:**

Reviewer #1: I appreciate the authors detailed responses to my comments and the new data to address my previous concerns. The majority of their points were clear and resolved outstanding concerns, however, their disclosure related to their inability to detect RfxCas13d in the brain concerns me and potentially casts doubt on the methods behind the manuscript’s central claim.

1. The authors are using a CAG-loxP-STOP-loxP cassette to express RfxCas13d conditionally. This is unquestionably a strong promoter that, when used to control Cas9 expression, should lead to its unambitious detection (https://doi.org/10.1016/j.cell.2014.09.014 among many studies).

It is concerning to me that, for a study claiming that in vivo collateral targeting in the brain leads to lethality, the authors do not show convincing data confirmed RfxCas13d expression in the brain (it is beyond odd to me that a magnetic bead pulldown assay is required to detect the protein from lysate). Further, the authors show widespread Cre expression throughout the brain, which one would expect would translate with widespread RfxCas13d expression, even if restricted to neurons. This is concerning because, without stronger data demonstrating RfxCas13d expression, it is conceivable the lethality could be resulting from a different mechanism. Perhaps a RfxCas13d variant? 

The bottom line is the authors unequivocally must demonstrate that RfxCas13d is expressed from their engineered mouse line. Not just for their conclusions, but also for the point of thoroughly characterizing their new mouse line.

**Response:** Thanks for your suggestions. First, we measured the mRNA levels of tdTomato, RfxCas13d and Cre in the brain tissues of the ^LSL^RfxCas13d^+/fl^Ai14^+/fl^ mice two weeks after injection of AAV-PHP.eB carrying hSYN-driven Cre. We found that although both tdTomato and RfxCas13d were driven by the same CAG promoter, the mRNA level of RfxCas13d was lower than that of tdTomato (Response Fig. 6a). Second, we strongly agree with that “the authors do not show convincing data confirmed RfxCas13d expression in the brain”. As we used self-made anti-HA antibody in our previous manuscript, we reasoned that the antibody might be the key issue. We thus ordered the commercial anti-HA antibody (11867423001, Roche) to re-detect the expression of RfxCas13d in the brain tissue. The band of RfxCas13d was detected (Response Fig. 6b). Moreover, we further performed immunohistochemical experiments using this antibody and found that RfxCas13d was expressed in most of the Cre-positive cells, which is consistent with our experimental design (Response Fig. 6c). Besides, we designed four pairs of primers to determine whether there were other possible variants of RfxCas13d (Response Fig. 6d). Results showed that no band other than the target bands was amplified from the brain tissue, indicating no other variants of RfxCas13d in our model (Response Fig. 6e). And according to our results in Fig 3, the expression of RfxCas13d is necessary for the lethality of mouse. In conclusion, the expression of RfxCas13d can be detected in the brain tissue and is required for the lethality.

**Response Figure 6**. **a.** RT-qPCR to measure the RNA level of *Actb*, *Cre*, *tdTomato* and *RfxCas13d* in the brain tissue from ^LSL^RfxCas13d^+/fl^Ai14^+/fl^ mice two weeks after injection of AAV-PHP.eB carrying hSYN-driven Cre (n=3). The numbers above the histogram represent the average. **b.** Western blot to measure the protein level of RfxCas13d in the brain tissue from ^LSL^RfxCas13d^+/fl^Ai14^+/fl^ mice two weeks after injection of AAV-PHP.eB carrying hSYN-driven Cre. **c.** Representative images showing coimmunostaining of Cre (1:1000, 15036T, CST) and RfxCas13d-HA (1:500, 11867423001, Roche) in the cortex, thalamus and hypothalamus (sagittal section) of ^LSL^RfxCas13d^fl/fl^ mice two weeks after injection of AAV-PHP.eB carrying hSYN-driven Cre (Flag tag). Scale bar: 20 μm. **d.** Schematic illustration of the design of primers for identifying the transcript variants of RfxCas13d in the brain tissue ^LSL^RfxCas13d^+/fl^Ai14^+/fl^ mice two weeks after injection of AAV-PHP.eB carrying hSYN-driven Cre. **e.** Gel picture of PCR products using primers in **d**.

2. Related to the new data on AAV-delivered RfxCas13d (shown in Response Figure 6). Numerous important details are missing that key for interpreting the data. One, how old were the animals when injected? It is odd that the non-targeted group is, over the course of the 20 days, not losing or not gaining weight. Two, the study should be balanced with an equal number of mice, preferably equal numbers of males and females. As males weigh more than females, this can heavily skew a weight analysis.

**Response:** We apologize for the lack of details. Six 12-week-old littermates were used for this experiment. Each group contained two males and one female. Due to the deadline for revision, we only recorded the weight of mice up to day 18 after the virus injection. The figure below showed the result of our continuous recording up to day 49. We have made a mistake at labeling of the Y axis in the body weight figures in previous manuscript. We corrected this in the revised manuscript. In the NT crRNA group, mice experienced slight body weight fluctuations (decline-rise-decline-rise) after the virus injection. We speculate that this may be related to the side effects of the overexpression of RfxCas13d, although more experiments are still needed to prove this.

**Response Figure 7**. **a.** Body weight change curve of WT mice after injection of AAV-PHP.eB carrying CMV-driven RfxCas13d and U6-driven Sik3-S crRNA 8 (n=3) or NT crRNA (n=3). Two-tail unpaired t test was used to compare body weight change between these two groups at indicated dpi. Significance levels are noted as *P < 0.05, **P < 0.01 or ns (P > 0.05). All values are presented as mean ± SEM.

3. On Pg. 21, the authors suggest several potential mechanisms about why they, but not other groups, observed lethality. I believe there speculative list is incomplete. Other potential explanations could be: differences in target transcript threshold that could affect the extent of collateral targeting (analogous to the in vitro effect); differences in the strength of RfxCas13d expression from differences in promoter strength, differences in copy number per cell due to differences in vector dosing or serotype and finally the relative ratio of Cas13 to target transcript, which can be affected by delivery.
**Response:** Thanks for your additions to make our speculation more detailed, and we have added these in the manuscript: “3. Differences in the expression levels of target transcripts may affect the extent of collateral activity (analogous to the *in vitro* effect). 4. Differences in promoter strength, vector dosing and serotype may cause the different strength of RfxCas13d expression.” (Revised Manuscript page 18 lines 392-395)

4, Since originally reviewed, a high-fidelity version of RfxCas13d has been reported in the literature. This variant should be highlighted in the Discussion.

**Response:** Thanks for your suggestions and we have highlighted these recent published papers and preprints about solutions to the collateral activity of RfxCas13d in the Discussion: “More recently, Tong et al. developed high-fidelity Cas13 variants that showed similar RNA knockdown activity to wild-type Cas13 but no detectable collateral damage in transgenic mice or adeno-associated-virus-mediated somatic cell targeting (37). Kelley et al. introduced gRNA excision for negative-autoregulatory optimization (GENO) to attenuate Cas13d expression, which mitigated collateral effects in human cells while retaining modest on-target knockdown of CUGn RNA (47). With deep learning model interpretation, Wei et al. revealed specific sequence motifs at spacer position 15-24 along with favored secondary features for highly efficient guides. Furthermore, they identified DjCas13d, a highly efficient ortholog with minimal detectable cellular toxicity when targeting highly-expressed transcripts in mammalian cells (48). Those high-fidelity Cas13 variants or optimization with minimal collateral effects would be better choices for targeted degradation of RNAs in basic research and therapeutic applications. Moreover, the newly discovered Cas7-11 is also a promising candidate for transcriptome engineering (21).” (Revised Manuscript page 22 lines 465-476).

**Reference:**

1.	Muzumdar MD, Tasic B, Miyamichi K, Li L, Luo L. A global double-fluorescent Cre reporter mouse. Genesis. 2007;45(9):593-605.

2.	Ai Y, Liang D, Wilusz JE. CRISPR/Cas13 effectors have differing extents of off-target effects that limit their utility in eukaryotic cells. Nucleic Acids Res. 2022;50(11):e65.

3.	Kelley CP, Haerle MC, Wang ET. Negative autoregulation mitigates collateral RNase activity of repeat-targeting CRISPR-Cas13d in mammalian cells. Cell Rep. 2022;40(7):111226.

4.	Tong H, Huang J, Xiao Q, He B, Dong X, Liu Y, et al. High-fidelity Cas13 variants for targeted RNA degradation with minimal collateral effect. bioRxiv. 2021.

5.	Shi P, Murphy MR, Aparicio AO, Kesner JS, Fang Z, Chen Z, et al. RNA-guided cell targeting with CRISPR/RfxCas13d collateral activity in human cells. bioRxiv. 2021.

6.	Xu C, Zhou Y, Xiao Q, He B, Geng G, Wang Z, et al. Programmable RNA editing with compact CRISPR-Cas13 systems from uncultivated microbes. Nat Methods. 2021;18(5):499-506.

7.	Ozcan A, Krajeski R, Ioannidi E, Lee B, Gardner A, Makarova KS, et al. Programmable RNA targeting with the single-protein CRISPR effector Cas7-11. Nature. 2021;597(7878):720-5.

8.	Yip BH. Recent Advances in CRISPR/Cas9 Delivery Strategies. Biomolecules. 2020;10(6).

**Third round of review**

**Reviewer 1**

The authors have thoroughly addressed my comments. The major conclusions of the manuscript, that CasRx-induced collateral targeting can trigger adverse effects in transgenic rodents, is supported by the data, though I continue to find it curious that RNA-seq measurements in transformed cells against endogenously expressed targets revealed few significant DEGs (Fig. 1C).

I do have two final critiques. One is related to the title of the study. As it is currently constructed, the title may be a tad controversial. As the authors observed that only select guides triggered lethality, and as other groups have observed no lethality in studies involving the delivery of CasRx with a guide, I suggest the authors use a more conservative title that eludes to the fact that their studies were performed in a CasRx transgenic line and that lethality was only sometimes observed and likely dependent on the crRNA. I recommend the authors consider something more along the lines of, The collateral activity of RfxCas13d can induce lethality in a RfxCas13d knock-in mouse model.

And two, it does not appear to be that the new data provided in the Response Letter is part of the revised manuscript (for example, Response Figure 6, which should be part of SI Fig. 2). All of these data, which were generated in response to the Reviewer critiques, should be included at the very least in the SI of the manuscript.

**Reviewer 2**

The authors have addressed my concerns, but I still have some suggestions before this paper is published.

Introduction:
Lines 86-91. This paragraph should be updated. Collateral activity in mammalian cells has been already demonstrated and published in several independent reports (PMID: 35244715, PMID: 35977479, including Tong et al 2022 (Nat Biotechnology, PMID: 35953673). This manuscript has confirmed what has been shown and published previously. On the other hand, this manuscript extends our knowledge about collateral activity (28S fragmentation etc). Please rephrase the last paragraphs of the introduction according to this.

Discussion:
Line 370 rephrase: our results confirmed that RfxCas13d exhibited collateral activity in mammalian cells
Please update Tong et al 2022 preprint as (Nat Biotechnology, PMID: 35953673). There are several paragraphs in the discussion where it is cited as preprint.
I would also suggest adding response figure 4 to the manuscript as Supp. Fig. This is a great control showing that Cas13d alone does not trigger collateral activity or other intrinsic toxic effects.

**Response to Reviewer #1:**

Reviewer #1: The authors have thoroughly addressed my comments. The major conclusions of the manuscript, that CasRx-induced collateral targeting can trigger adverse effects in transgenic rodents, is supported by the data, though I continue to find it curious that RNA-seq measurements in transformed cells against endogenously expressed targets revealed few significant DEGs (Fig. 1C).

**Response:** To figure out whether RfxCas13d exhibited collateral activity in N2a cells when targeting endogenously lowly-expressed genes (*Sik3-S*, *Map2*, *Mapt* and *Rbfox3*), we, as suggested by Reviewer 2#, performed RNA-seq using RfxCas13d/dRfxCas13d with or without crRNA in N2A cells and identified DEGs by comparing their transcriptome profiles, although these genes were expressed at low levels in N2a and lower than in brain (Additional file 1: Fig. S12a). 6 DEGs were found in comparison between dRfxCas13d and RfxCas13d alone, and 194~1179 DEGs were identified in comparisons between dRfxCas13d with crRNA and RfxCas13d with crRNA (Additional file 1: Fig. S12b and Table S3). This indicated that RfxCas13d alone only had little effect on the transcriptome, whereas RfxCas13d equipped with crRNA did.

Since only the HEPN domains were mutated in dRfxCas13d, these DEGs were associated with the HEPN domains, the executor of on-target, off-target and collateral activity. In our opinion, the on-target and off-target effects of RfxCas13d are spacer sequence-dependent, while the substrate selection by collateral activity, to a certain extent, is spacer sequence-independent. Thus, through analysis, we identified 185 common DEGs from seven sets of comparisons (Group 3-9 in Additional file 1: Fig. S12b) between dRfxCas13d with targeting crRNA and RfxCas13d with targeting crRNA (Table S3). These DEGs were spacer sequence-independent and therefore were thought to be associated with collateral activity.

To investigate the function of these DEGs, we performed GO term enrichment analysis and found that these genes were significantly enriched in nucleosome assembly (cluster 1) and ribosome (cluster 2) (Additional file 1: Fig. S12c). And these nucleosome and ribosome-related genes were up-regulated in all seven sets of comparisons (Additional file 1: Fig. S12d). Consistently, Tong et al. recently reported that up-regulated genes induced by RfxCas13d targeting RPL4 and PPIA can be enriched in nucleosome assembly(1). Moreover, multiple histone genes and a few ribosome-related genes were found in the DEGs caused by RfxCas13d in HEK293T and U87 cells(2).

As suggested by the reviewer 2#, we also compared the transcriptomes between dRfxCas13d with NT crRNA and dRfxCas13d with targeting crRNA, and no or few (3 and 17) DEGs were found, indicating that crRNA alone had little effect on the transcriptome (Additional file 1: Fig. S12d).

These data suggested that these nucleosome and ribosome-related genes were associated with collateral activity, but how collateral activity affected the expression of these genes remained to be further investigated.

We have added these results (Additional file 1: Fig. S12) and related descriptions in the Discussion section to explain the question you raised.

I do have two final critiques. One is related to the title of the study. As it is currently constructed, the title may be a tad controversial. As the authors observed that only select guides triggered lethality, and as other groups have observed no lethality in studies involving the delivery of CasRx with a guide, I suggest the authors use a more conservative title that eludes to the fact that their studies were performed in a CasRx transgenic line and that lethality was only sometimes observed and likely dependent on the crRNA. I recommend the authors consider something more along the lines of, The collateral activity of RfxCas13d can induce lethality in a RfxCas13d knock-in mouse model.

**Response:** Thanks for your suggestions and we have changed the title as you suggested.

And two, it does not appear to be that the new data provided in the Response Letter is part of the revised manuscript (for example, Response Figure 6, which should be part of SI Fig. 2). All of these data, which were generated in response to the Reviewer critiques, should be included at the very least in the SI of the manuscript.

**Response:** Thanks, we have complied all content of the two rounds of revision as Additional file 3 for the convenience of readers. Meanwhile, we have added Response figures to the main and supplementary figures as much as possible, while keeping the logic flowing. As shown below:

Response Fig. 1 (First revision) was added as Additional file 1: Fig. S2.

Response Fig. 5 (First revision) was added as Fig. 1h.

Response Fig. 7 (First revision) was added as Additional file 1: Fig. S10.

Response Fig. 11 (First revision) was added as Additional file 1: Fig. S7c-d.

Response Fig. 12 (First revision) was added as Additional file 1: Fig. S11.

Response Fig. 1 (Second revision) was added as Fig. 4c-d.

Response Fig. 2 (Second revision) was added as Fig. 4h-m and Additional file 1: Fig. S7i-p.

Response Fig. 4 (Second revision) was added as Additional file 1: Fig. S12.

Response Fig. 6 (Second revision) was added as Additional file 1: Fig. S3.

**Response to Reviewer #2:**

Reviewer #2: The authors have addressed my concerns, but I still have some suggestions before this paper is published.

Introduction:

Lines 86-91. This paragraph should be updated. Collateral activity in mammalian cells has been already demonstrated and published in several independent reports (PMID: 35244715, PMID: 35977479, including Tong et al 2022 (Nat Biotechnology, PMID: 35953673). This manuscript has confirmed what has been shown and published previously. On the other hand, this manuscript extends our knowledge about collateral activity (28S fragmentation etc). Please rephrase the last paragraphs of the introduction according to this.

**Response:** Thanks, we have rephrased the according paragraph of the introduction as suggested by the reviewer. As shown below:

More recently, several studies reported that Cas13 exhibited collateral effects in mammalian cells, including one that further reported the lethality of Cas13 in mice. In line with those recent studies, we found that mice died when using RfxCas13d to knock down genes in brain neurons. RfxCas13d-mediated lethality may result from the collateral activity of RfxCas13d instead of the loss of target gene function or off-target effects. And we confirmed that RfxCas13d exhibited collateral activity in mammalian cells, which is positively correlated with the abundance of target RNA. Furthermore, we found that the collateral activity of RfxCas13d caused cleavage of 28s rRNA into two fragments, leading to translation attenuation and activation of the ZAKα-JNK/p38-IEG pathway.

Discussion:

Line 370 rephrase: our results confirmed that RfxCas13d exhibited collateral activity in mammalian cells

**Response:** Thanks, we have made changes as you suggested.

Please update Tong et al 2022 preprint as (Nat Biotechnology, PMID: 35953673). There are several paragraphs in the discussion where it is cited as preprint.

**Response:** Thanks, we have updated this reference and another reference (Cell Reports, PMID: 35977479, previously cited as a preprint)

I would also suggest adding response figure 4 to the manuscript as Supp. Fig. This is a great control showing that Cas13d alone does not trigger collateral activity or other intrinsic toxic effects.

**Response:** Thanks, we have added Response Fig 4 to Additional file 1 as Fig. S12. Since adding all the response figures generated during the two rounds of revision to the main figure and the supplementary figure may affect the logic of the paper, we have complied all content of the two rounds of revision as additional file 3 for the convenience of readers.

1.	Tong H, Huang J, Xiao Q, He B, Dong X, Liu Y, et al. High-fidelity Cas13 variants for targeted RNA degradation with minimal collateral effects. Nat Biotechnol. 2022.

2.	Ozcan A, Krajeski R, Ioannidi E, Lee B, Gardner A, Makarova KS, et al. Programmable RNA targeting with the single-protein CRISPR effector Cas7-11. Nature. 2021;597(7878):720-5.
